# Supplementary material for: Gene autoregulation by 3’ UTR-derived bacterial small RNAs
Source: eLife. 2020 Aug 3;9:e58836. doi: 10.7554/eLife.58836 (PMC7398697; doi:10.7554/eLife.58836)
Supplement: Figure 1—source data 1. [file elife-58836-fig1-data1.docx]

# Source data for Figure 1 Figure 1A


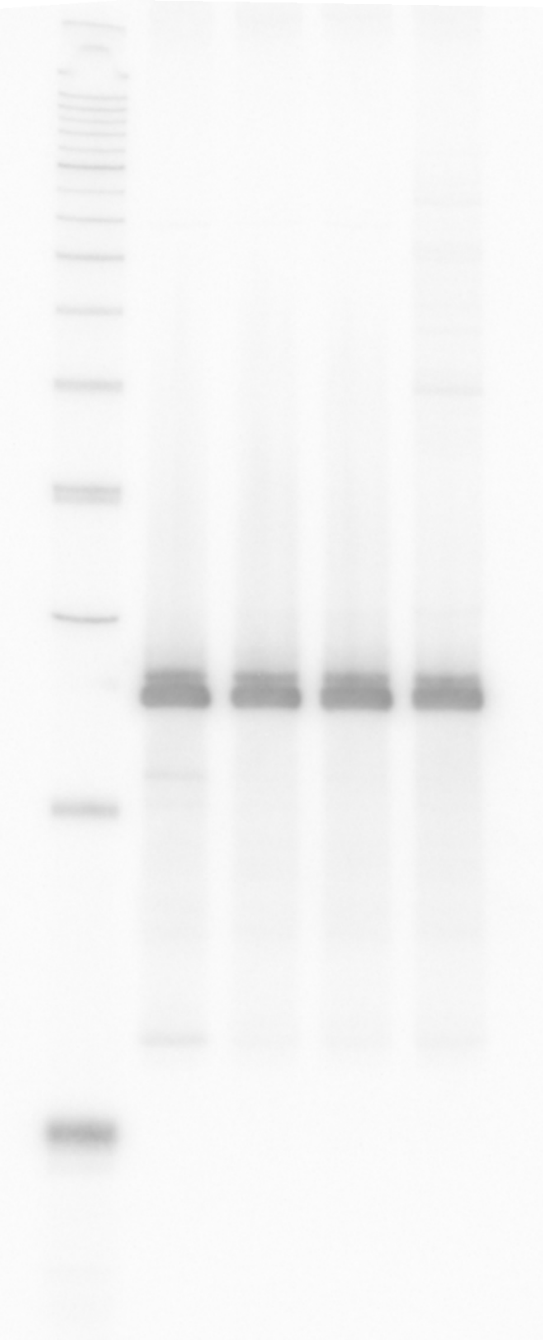


1 2 3 4 [lane]


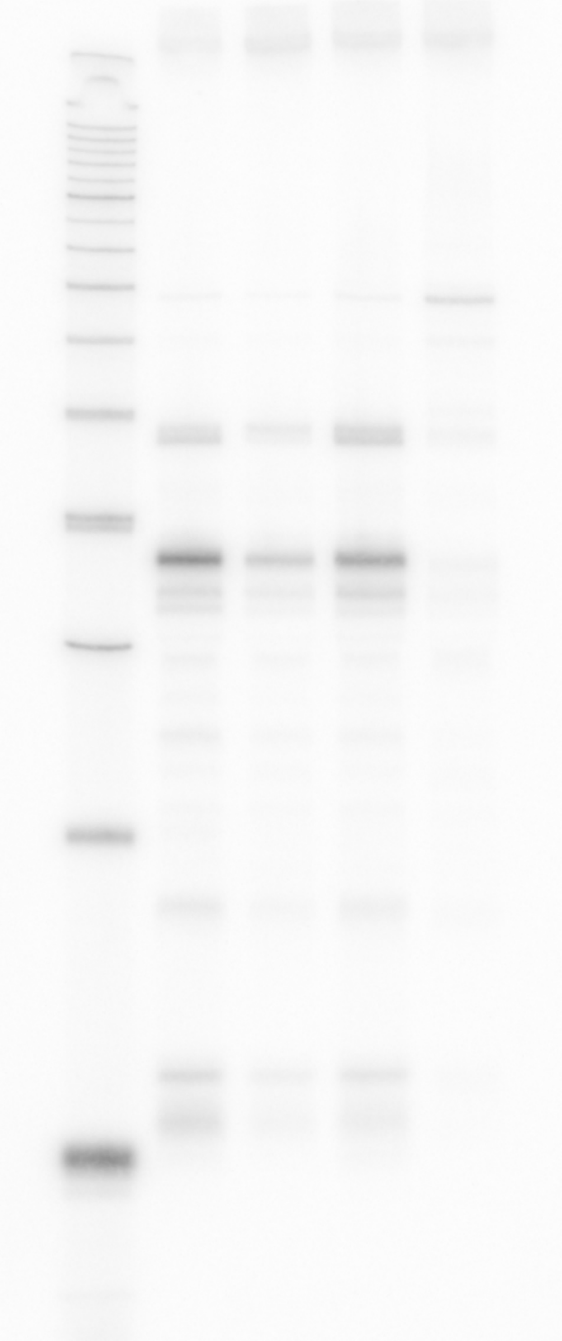


1 2 3 4 [lane]

5S (KPO-0243) MicX (KPO-0145)

# Figure 1B

Shown in Fig. 1B: 0-40 sites per gene (shaded in grey)

| **sites per gene** | **number of genes** |  | **sites per gene** | **number of genes** |  | **sites per gene** | **number of genes** |
| --- | --- | --- | --- | --- | --- | --- | --- |
| 0 | 1161 |  | 21 | 19 |  | 42 | 3 |
| 1 | 504 |  | 22 | 16 |  | 43 | 3 |
| 2 | 391 |  | 23 | 14 |  | 44 | 1 |
| 3 | 273 |  | 24 | 9 |  | 45 | 2 |
| 4 | 210 |  | 25 | 11 |  | 47 | 1 |
| 5 | 188 |  | 26 | 13 |  | 48 | 1 |
| 6 | 134 |  | 27 | 8 |  | 49 | 2 |
| 7 | 127 |  | 28 | 11 |  | 53 | 1 |
| 8 | 119 |  | 29 | 10 |  | 54 | 1 |
| 9 | 81 |  | 30 | 4 |  | 56 | 1 |
| 10 | 76 |  | 31 | 7 |  | 57 | 2 |
| 11 | 65 |  | 32 | 5 |  | 59 | 2 |
| 12 | 43 |  | 33 | 5 |  | 62 | 1 |
| 13 | 51 |  | 34 | 7 |  | 65 | 1 |
| 14 | 40 |  | 35 | 7 |  | 69 | 1 |
| 15 | 30 |  | 36 | 4 |  | 76 | 1 |
| 16 | 43 |  | 37 | 2 |  | 78 | 1 |
| 17 | 31 |  | 38 | 2 |  | 87 | 1 |
| 18 | 38 |  | 39 | 1 |  | 91 | 1 |
| 19 | 21 |  | 40 | 4 |  | 103 | 1 |
| 20 | 19 |  | 41 | 1 |  |  |  |

# Figure 1C

| **Feature** | **Sites** |
| --- | --- |
| CDS | 19330 |
| asRNA | 1975 |
| sRNA | 157 |
| 5‘ UTR | 2348 |
| 3‘ UTR | 1475 |
| IGR | 1109 |
