# Supplementary material for: Gene autoregulation by 3’ UTR-derived bacterial small RNAs
Source: eLife. 2020 Aug 3;9:e58836. doi: 10.7554/eLife.58836 (PMC7398697; doi:10.7554/eLife.58836)

Source data for Figure 1 – figure supplement 2

Figure 1 – figure supplement 2A

|  | **Reads uniquely aligned** | **Reads multiple aligned** | **Reads not aligned** |
| --- | --- | --- | --- |
| **WT 30° C Rep 1** | 8118175 | 5149909 | 2183202 |
| **WT 30° C Rep 2** | 7978348 | 4443912 | 1790452 |
| **WT 30° C Rep 3** | 6421157 | 7139591 | 3455617 |
| **WT 44° C Rep 1** | 7899645 | 5657897 | 1751161 |
| **WT 44° C Rep 2** | 6720733 | 7403221 | 1544384 |
| **WT 44° C Rep 3** | 7494113 | 5211431 | 1389189 |
| **rne 30° C Rep 1** | 9072901 | 4684223 | 2391032 |
| **rne 30° C Rep 2** | 6297723 | 4261754 | 3994005 |
| **rne 30° C Rep 3** | 9590527 | 577424 | 5076313 |
| **rne 44° C Rep 1** | 8604294 | 2616127 | 941700 |
| **rne 44° C Rep 2** | 8687439 | 3851302 | 1511141 |
| **rne 44° C Rep 3** | 1124259 | 4689093 | 2009175 |

Figure 1 – figure supplement 2B


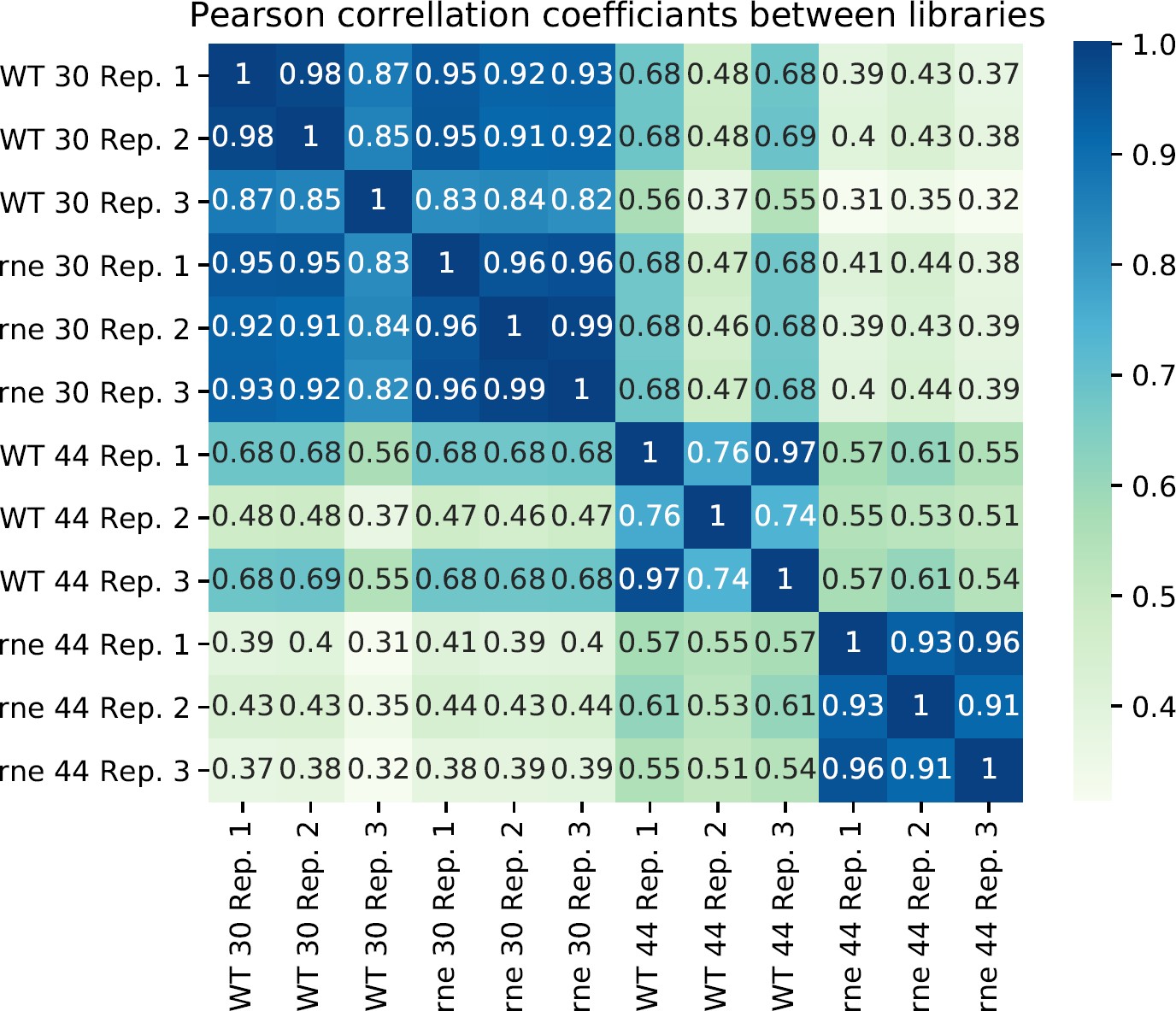

Supplement: Figure 1—figure supplement 2—source data 1. [file elife-58836-fig1-figsupp2-data1.docx]
