# Supplementary material for: Gene autoregulation by 3’ UTR-derived bacterial small RNAs
Source: eLife. 2020 Aug 3;9:e58836. doi: 10.7554/eLife.58836 (PMC7398697; doi:10.7554/eLife.58836)
Supplement: Figure 1—figure supplement 4—source data 1. [file elife-58836-fig1-figsupp4-data1.docx]

Source data for Figure 1 – figure supplement 4 Figure 1 – figure supplement 4


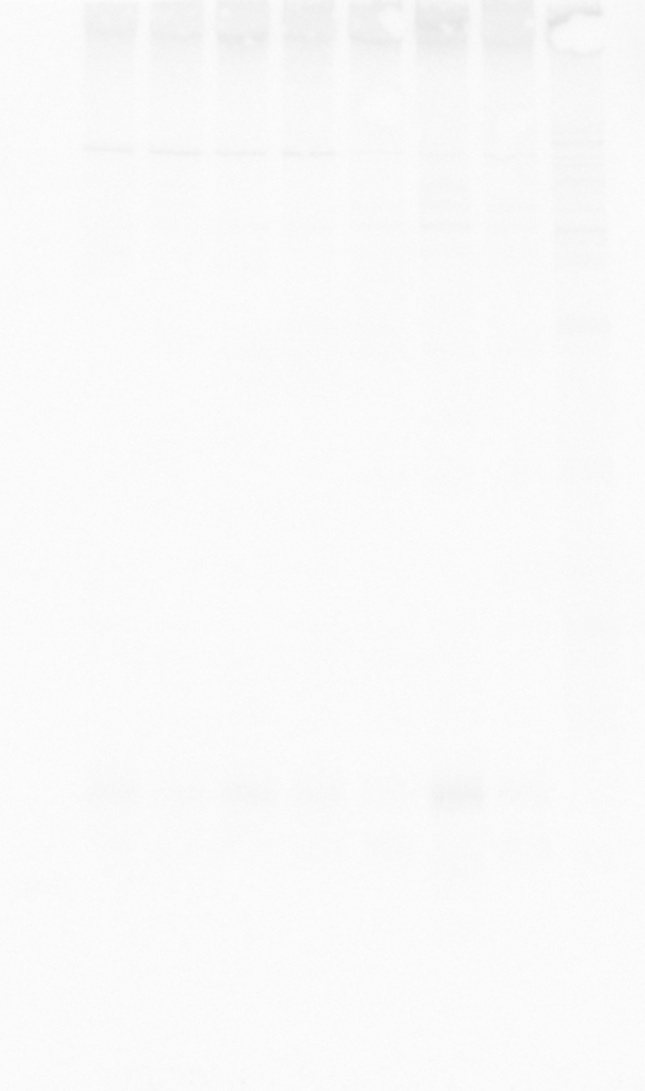


1 2 3 4

Vcr016

| **Northern blot** | **sRNA** | **probe** |
| --- | --- | --- |
| 1 | Vcr016 | riboprobe |


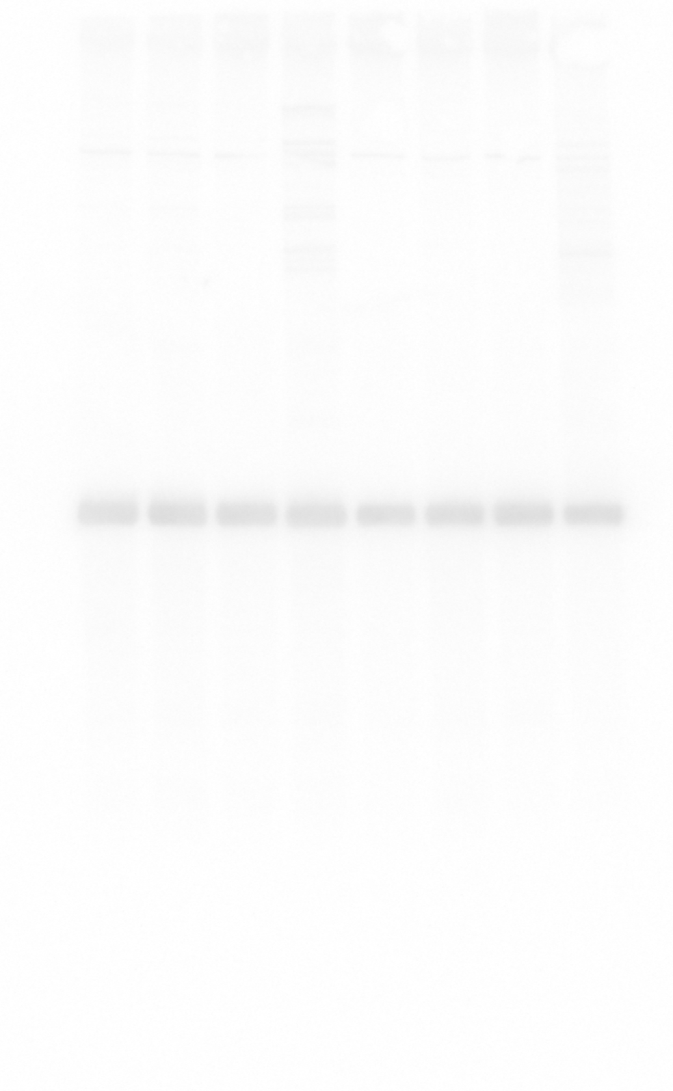


[lane]

1 2 3 4 [lane]

5S

| **Northern blot** | **sRNA** | **probe** |
| --- | --- | --- |
| 2 | Vcr041 | KPO-2025 |


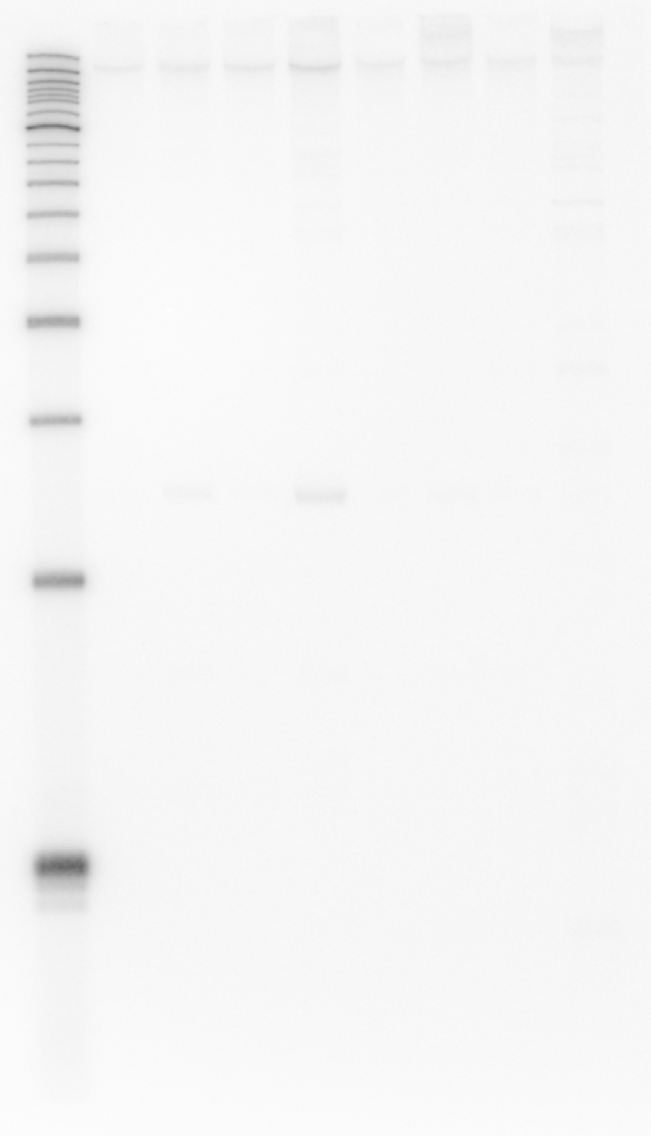


1 2 3 4

Vcr041


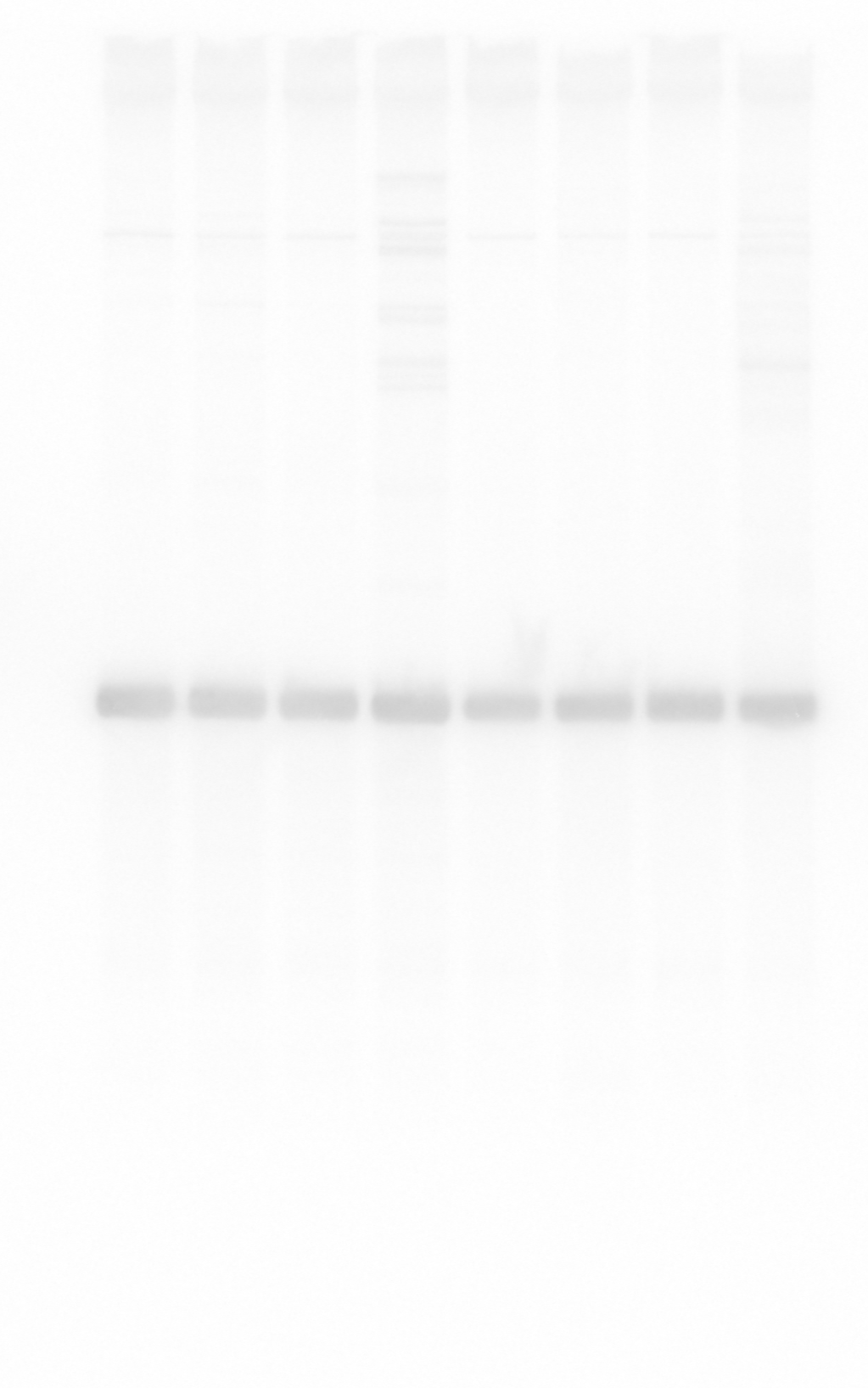


[lane]

1 2 3 4 [lane]

5S

| **Northern blot** | **sRNA** | **probe** |
| --- | --- | --- |
| 3 | Vcr044 | KPO-2026 |
| 3 | Vcr045 | KPO-0845 |
| 3 | Vcr053 | KPO-0852 |

[lane]


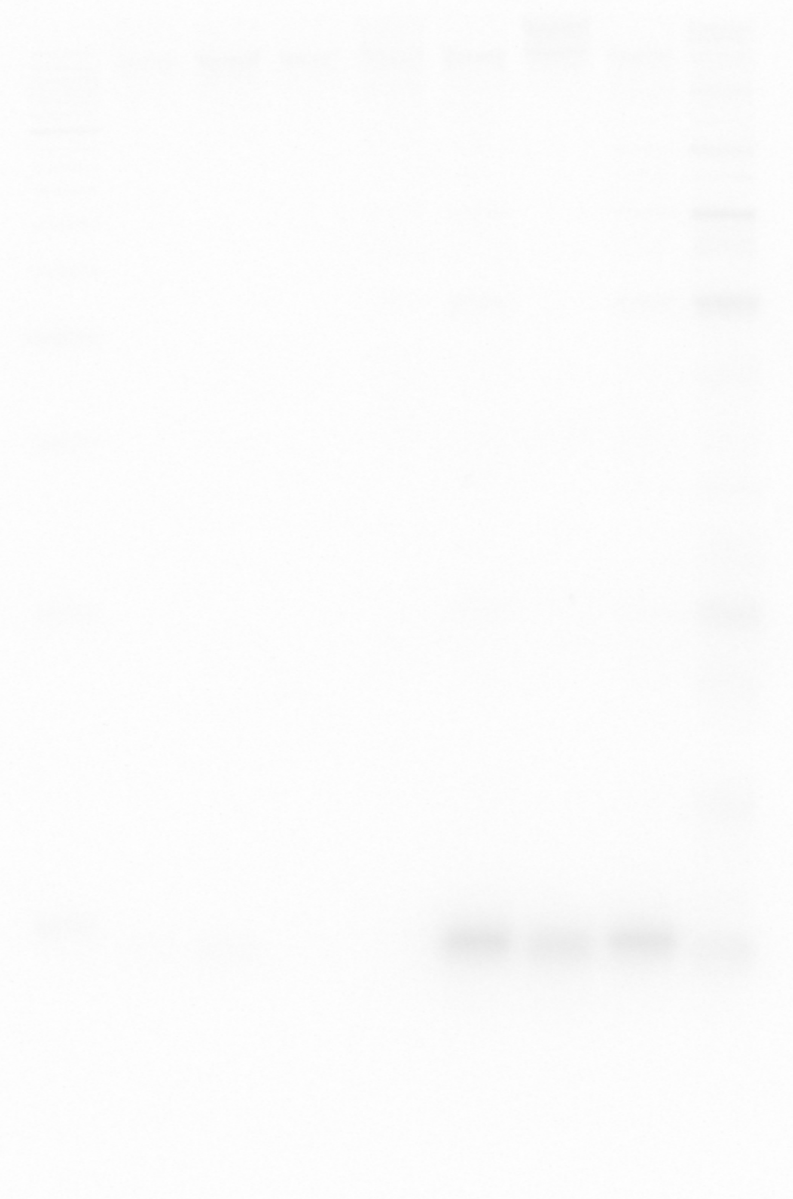

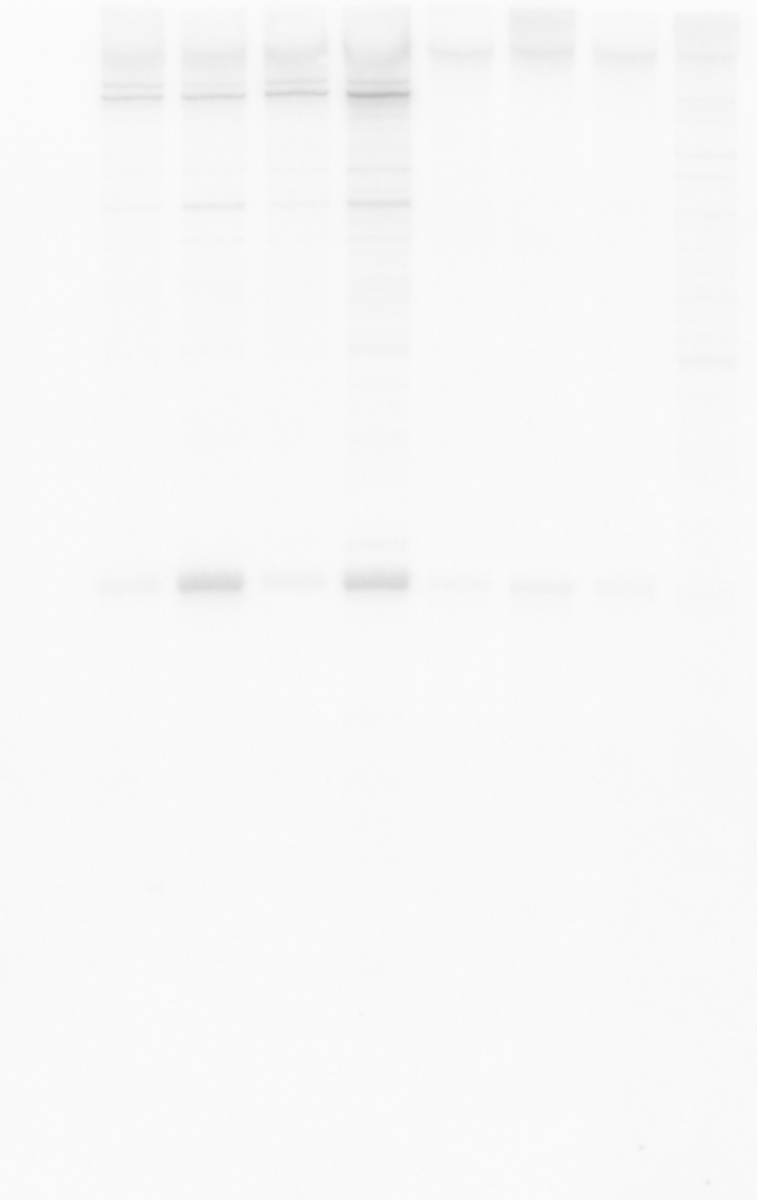


1 2 3 4

Vcr044

1 2 3 4 [lane]

Vcr045

1 2 3 4 [lane]


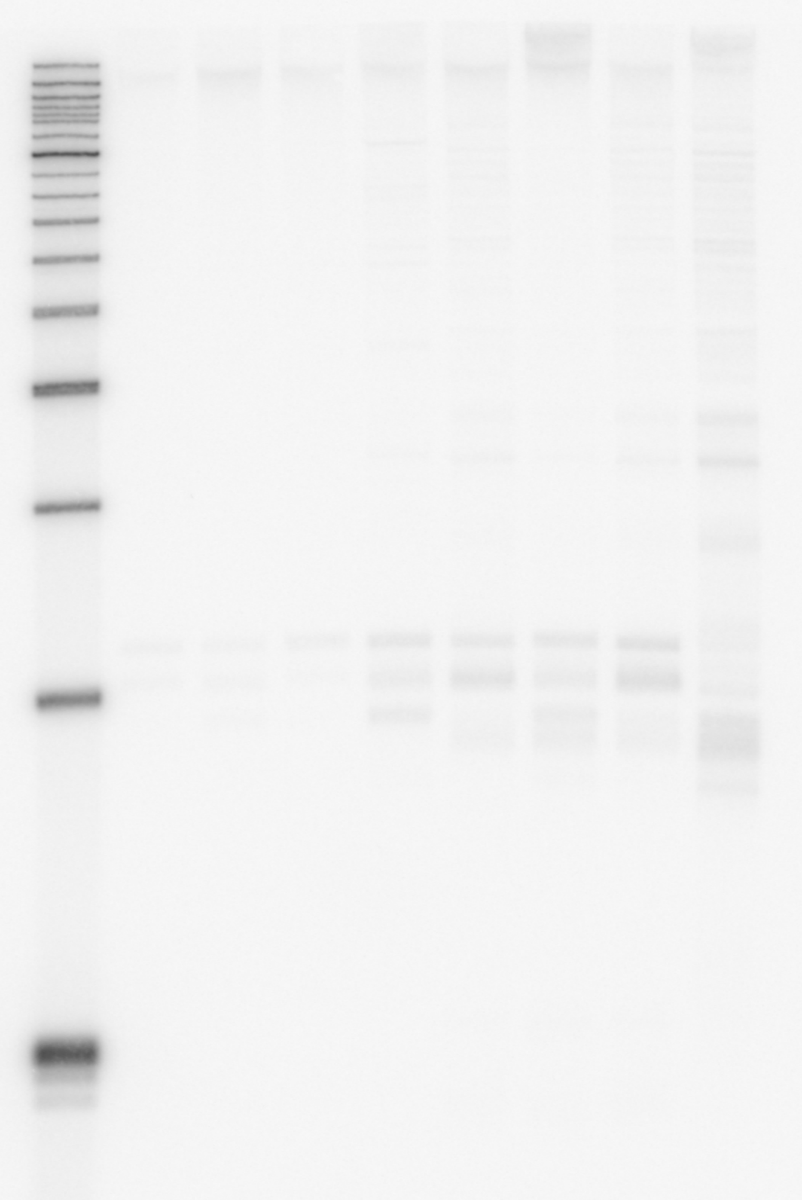


1 2 3 4 [lane]

Vcr053

5S


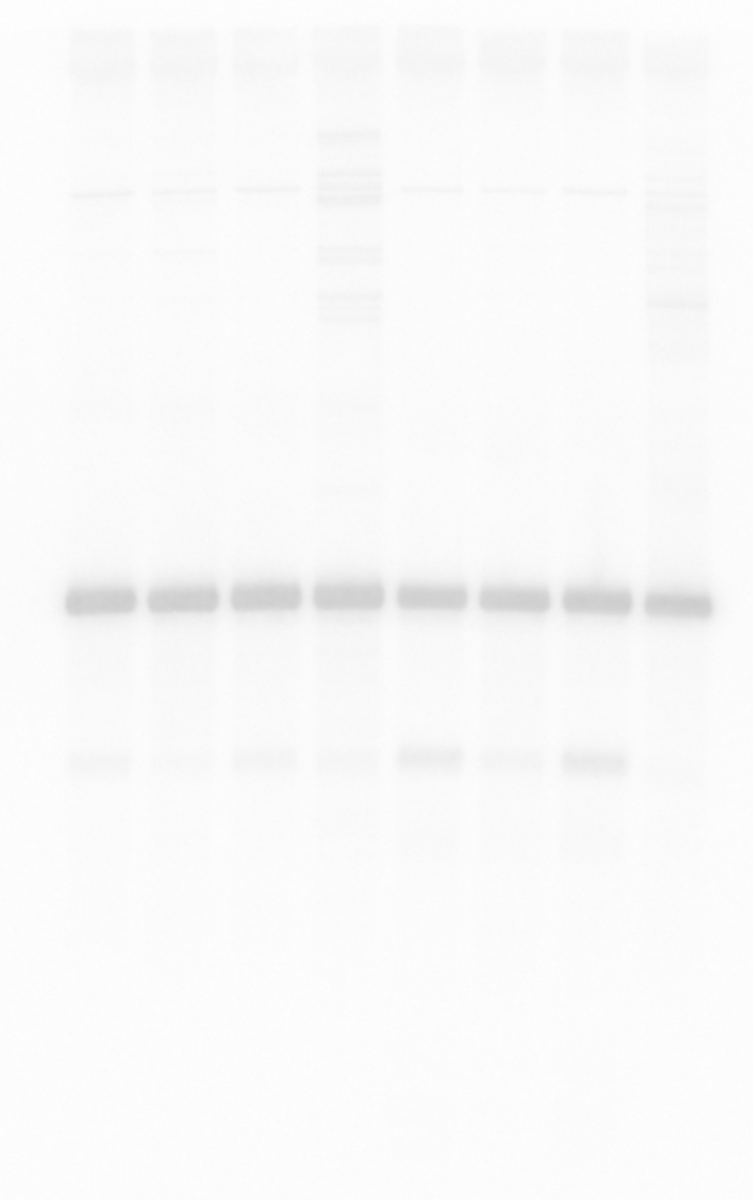


| **Northern blot** | **sRNA** | **probe** |
| --- | --- | --- |
| 4 | Vcr064 | KPO-0860 |
| 4 | FarS (Vcr076) | KPO-0873 |


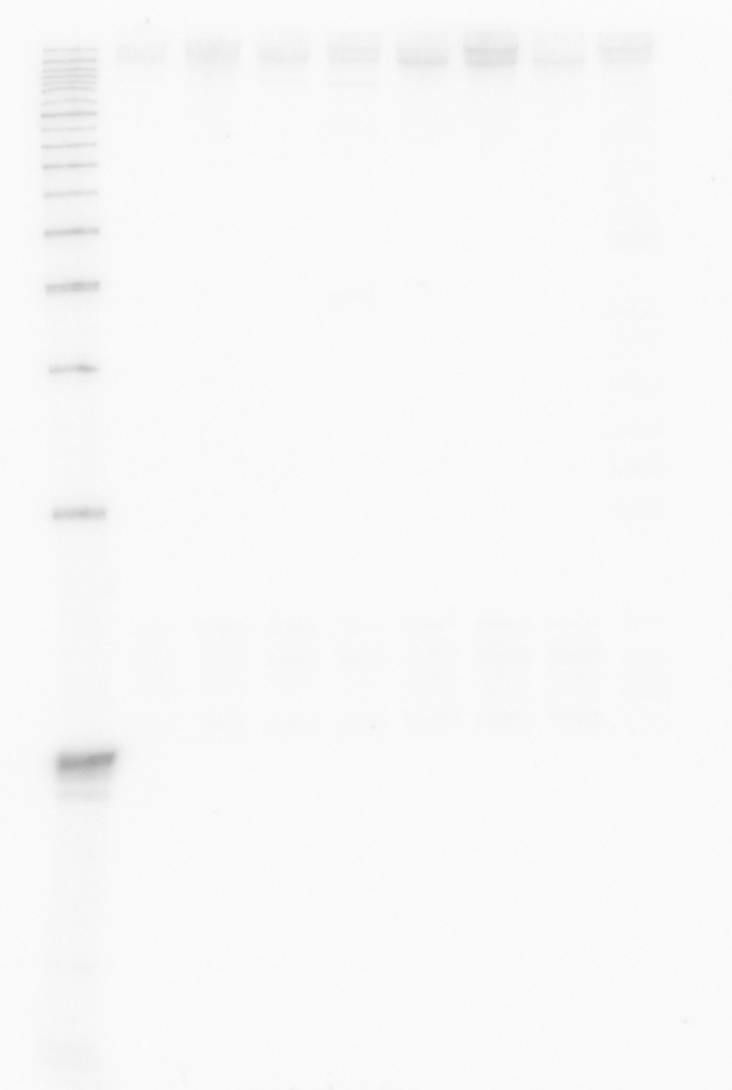


1 2 3 4 [lane]

Vcr064


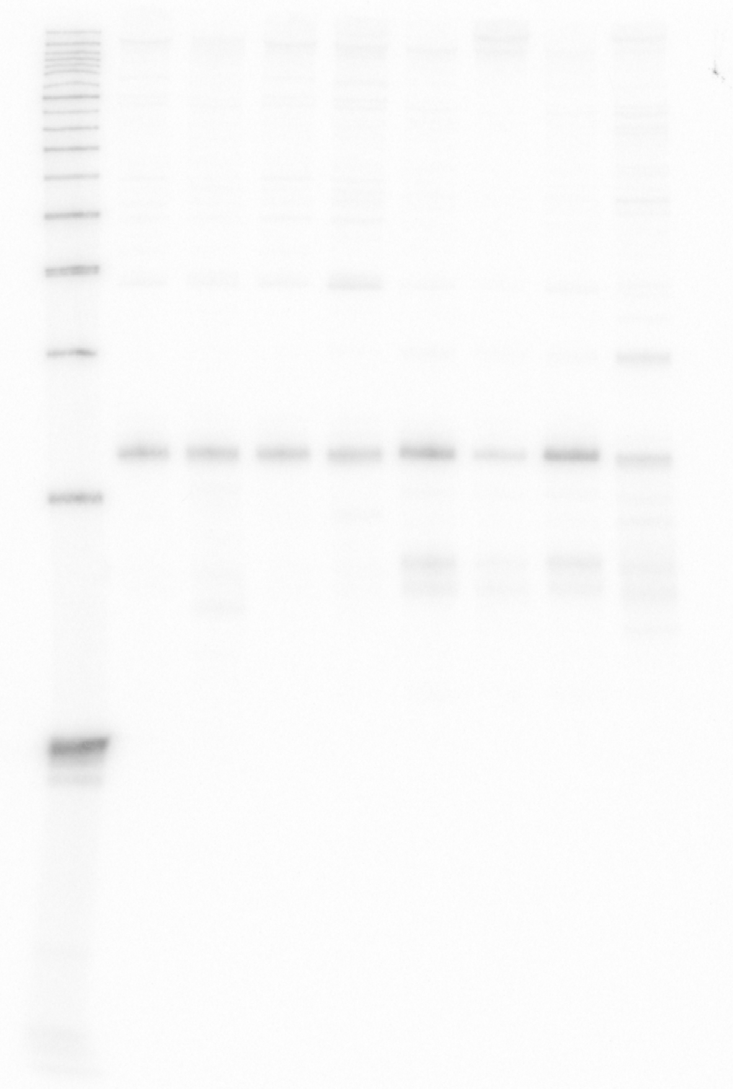


1 2 3 4 [lane]

Vcr076 (FarS)


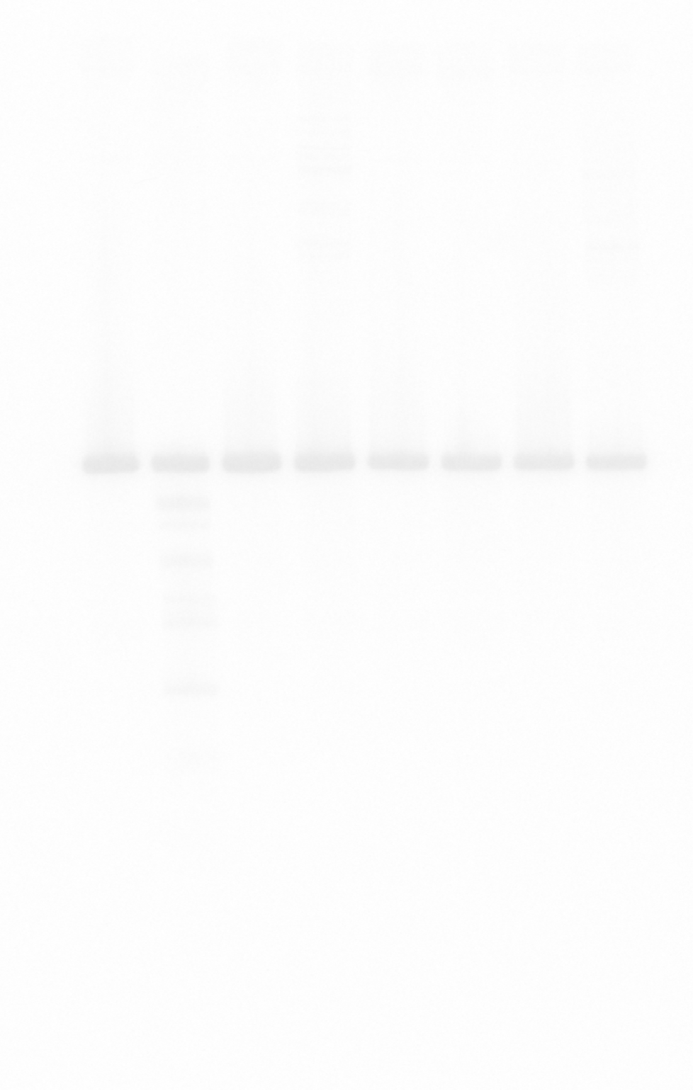


1 2 3 4 [lane]

5S

| **Northern blot** | **sRNA** | **probe** |
| --- | --- | --- |
| 5 | Vcr079 | KPO-0875 |
| 5 | Vcr084 | KPO-0821 |

1 2 3 4 [lane]


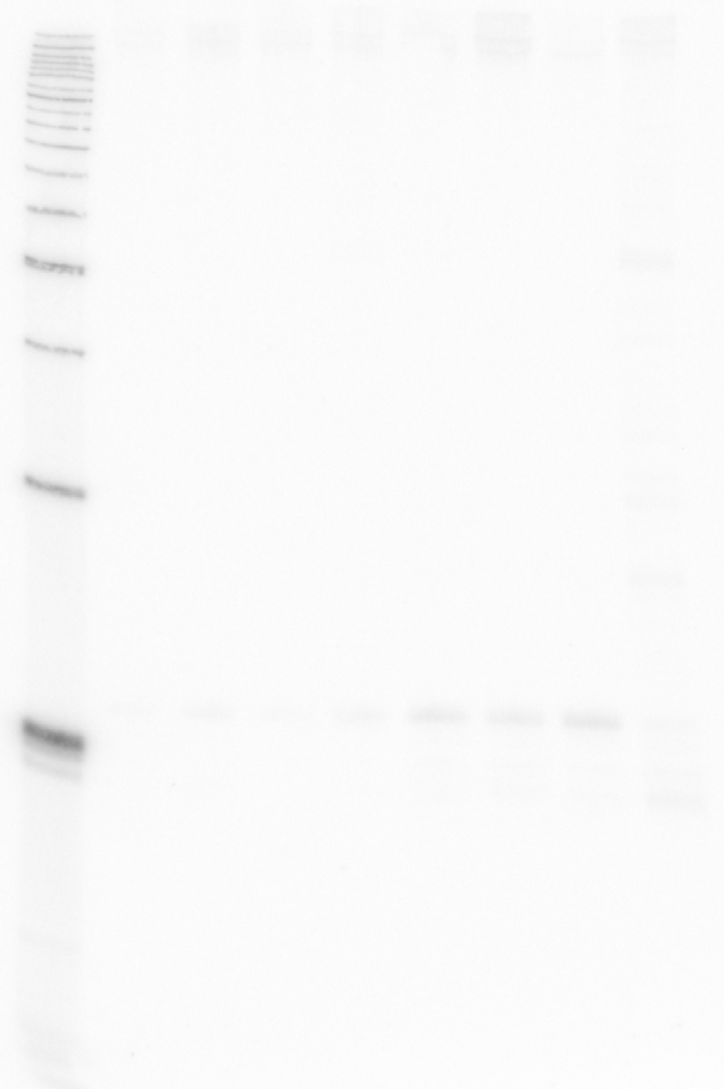

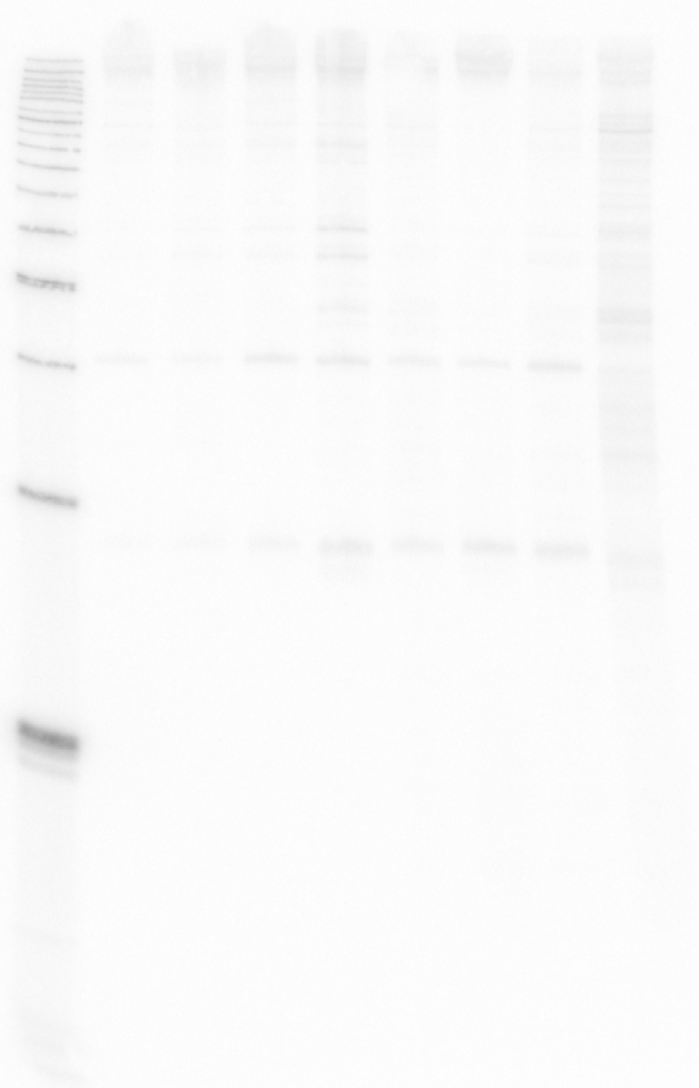


1 2 3 4 [lane]

Vcr079

Vcr084


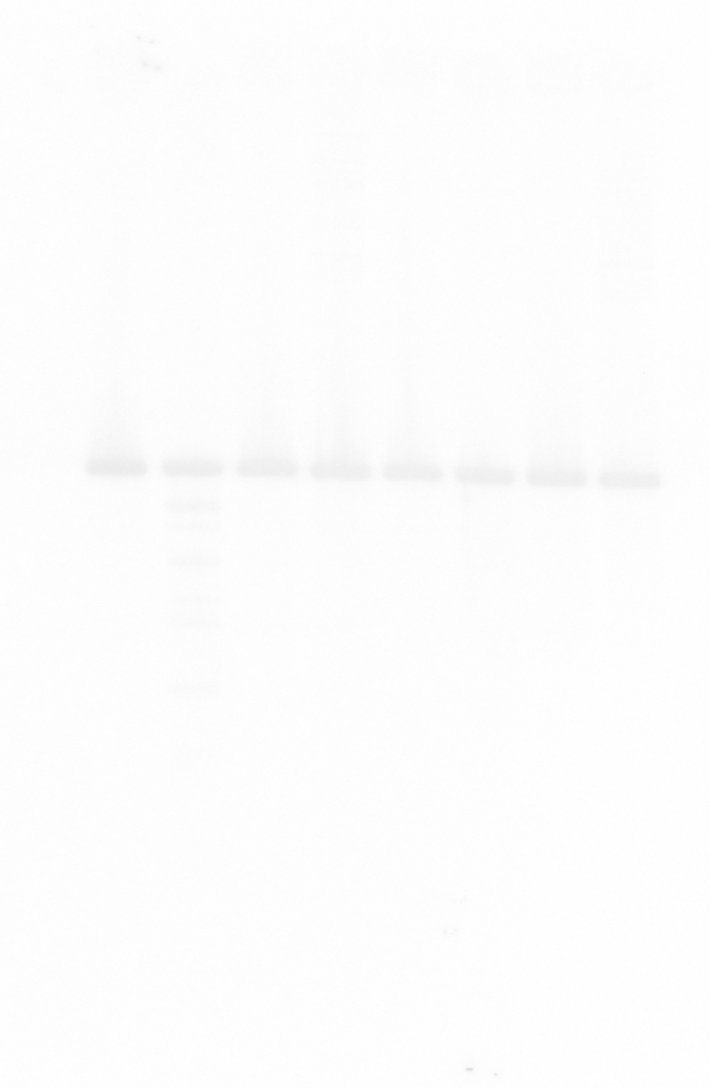


1 2 3 4 [lane]

5S
