# Supplementary material for: Gene autoregulation by 3’ UTR-derived bacterial small RNAs
Source: eLife. 2020 Aug 3;9:e58836. doi: 10.7554/eLife.58836 (PMC7398697; doi:10.7554/eLife.58836)

Source data for Figure 1 – figure supplement 5 Figure 1 – figure supplement 5

| **Northern blot** | **sRNA** | **probe** |
| --- | --- | --- |
| 6 | Vcr043 | KPO-0842 |

1 2 3 4 [lane]


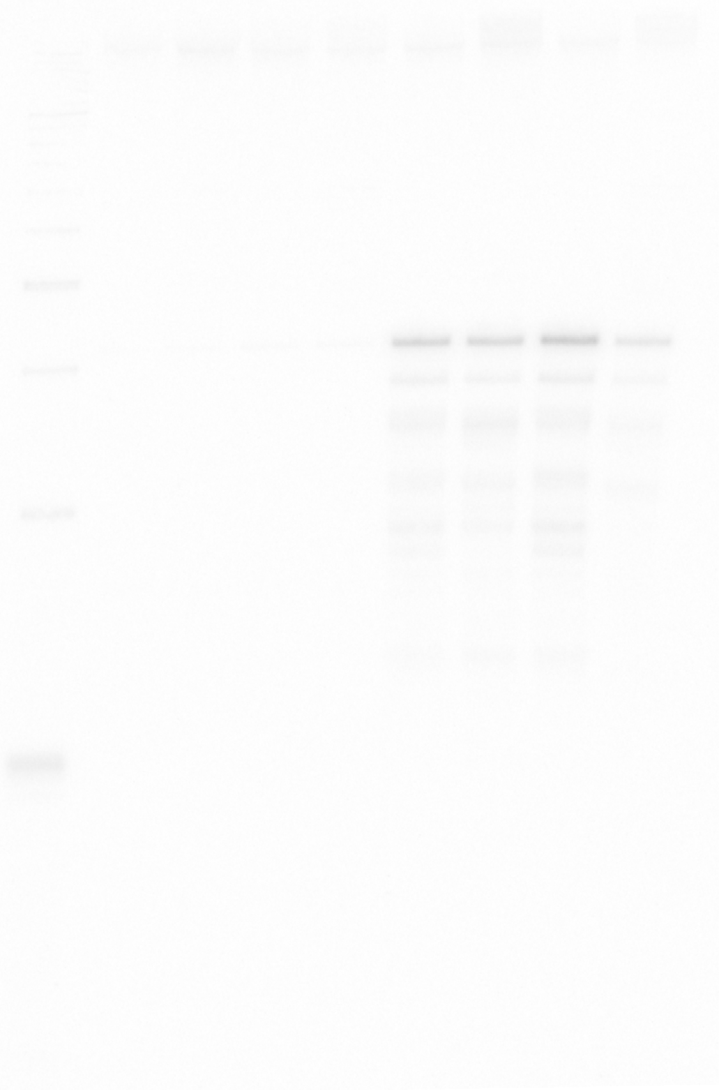

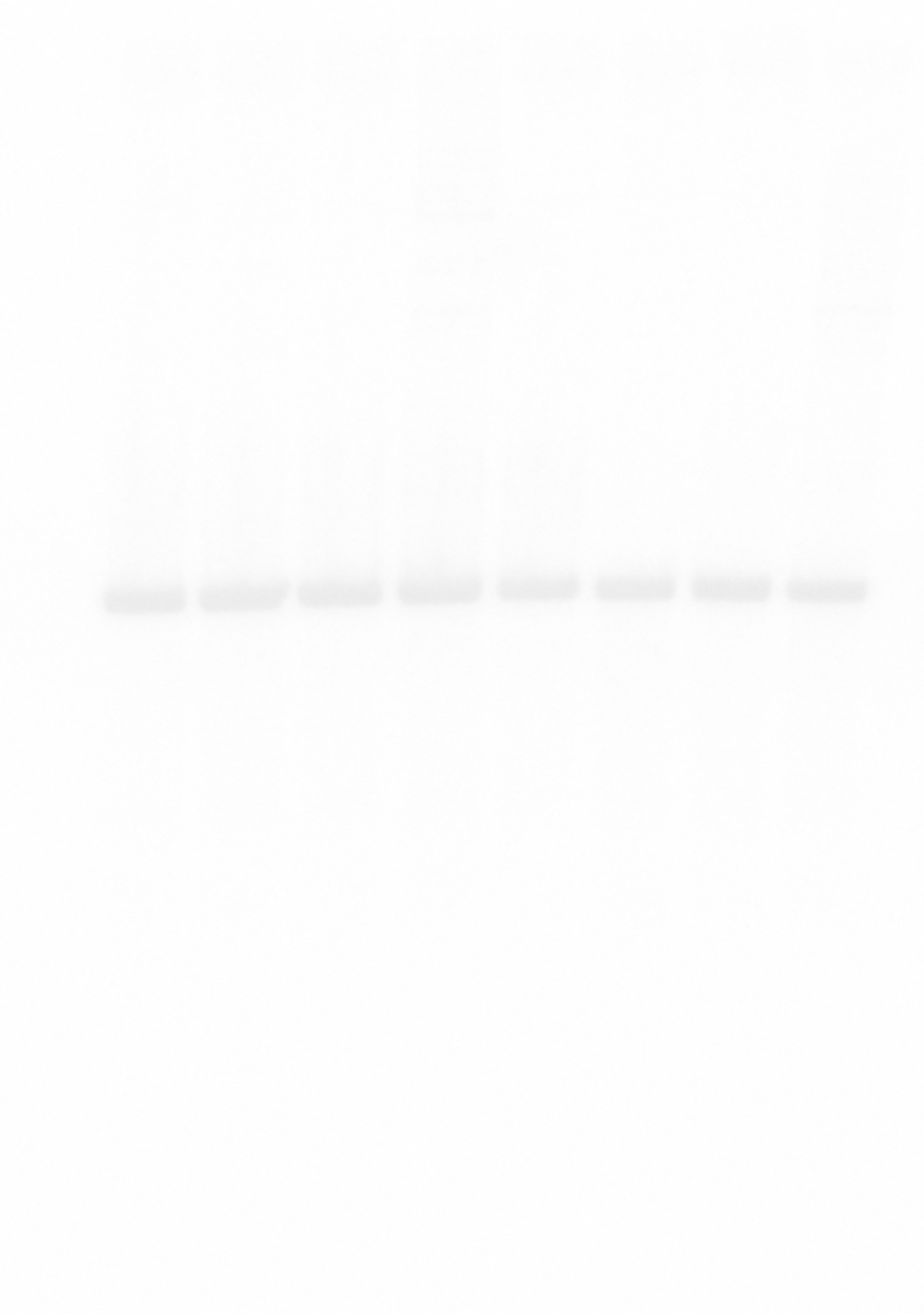


1 2 3 4 [lane]

5S

Vcr043


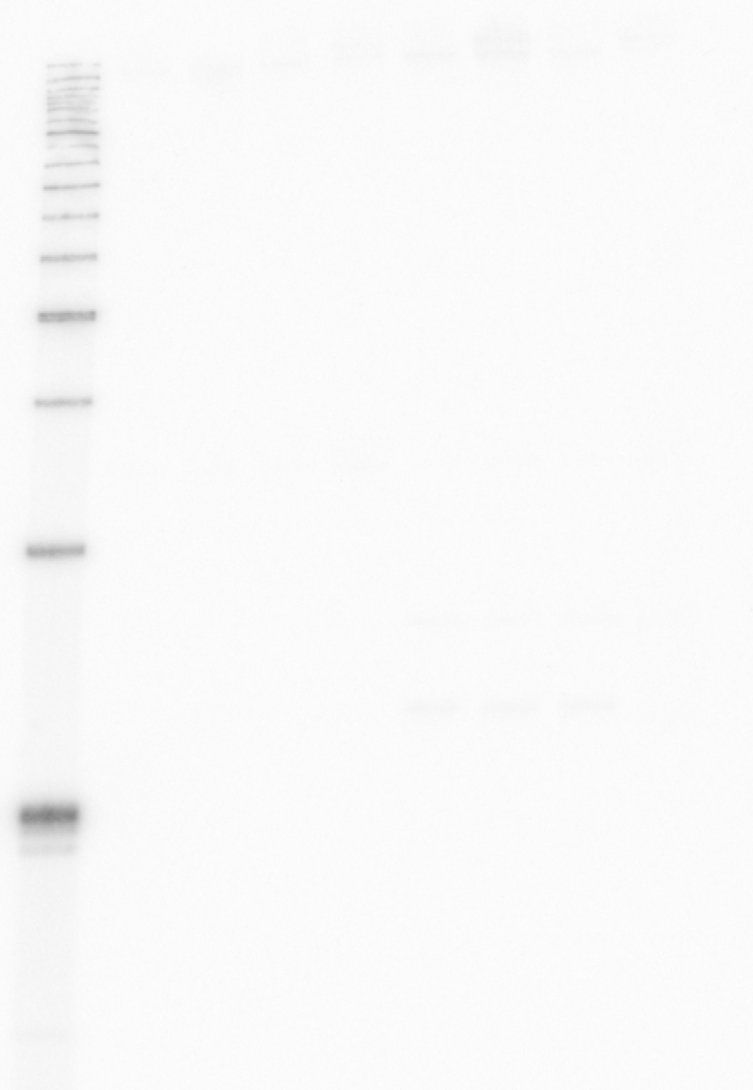


1 2 3 4 [lane]

Vcr065


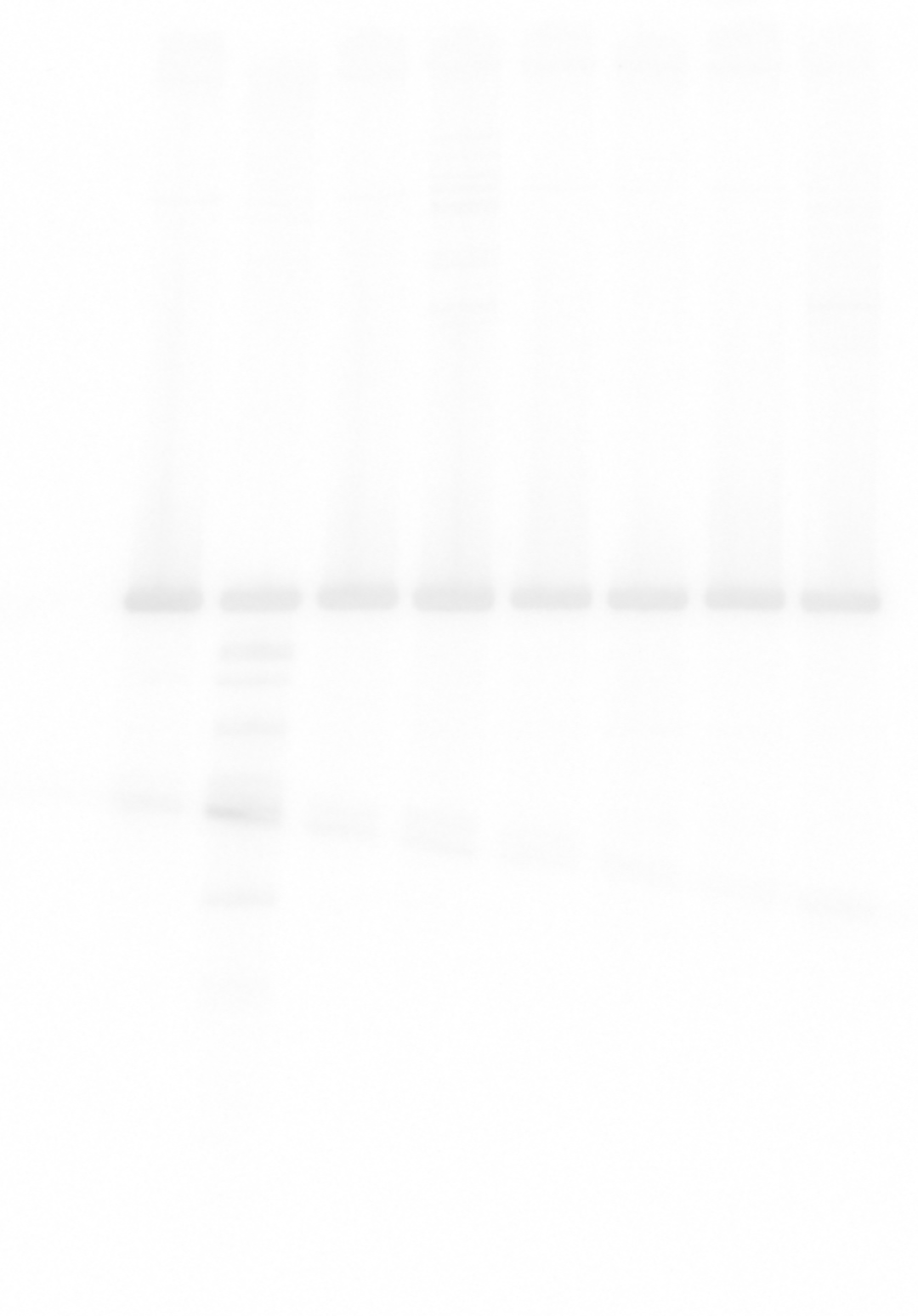


1 2 3 4

5S

| **Northern blot** | **sRNA** | **probe** |
| --- | --- | --- |
| 7 | Vcr065 | KPO-0861 |

[lane]

Figure 1 – figure supplement 5


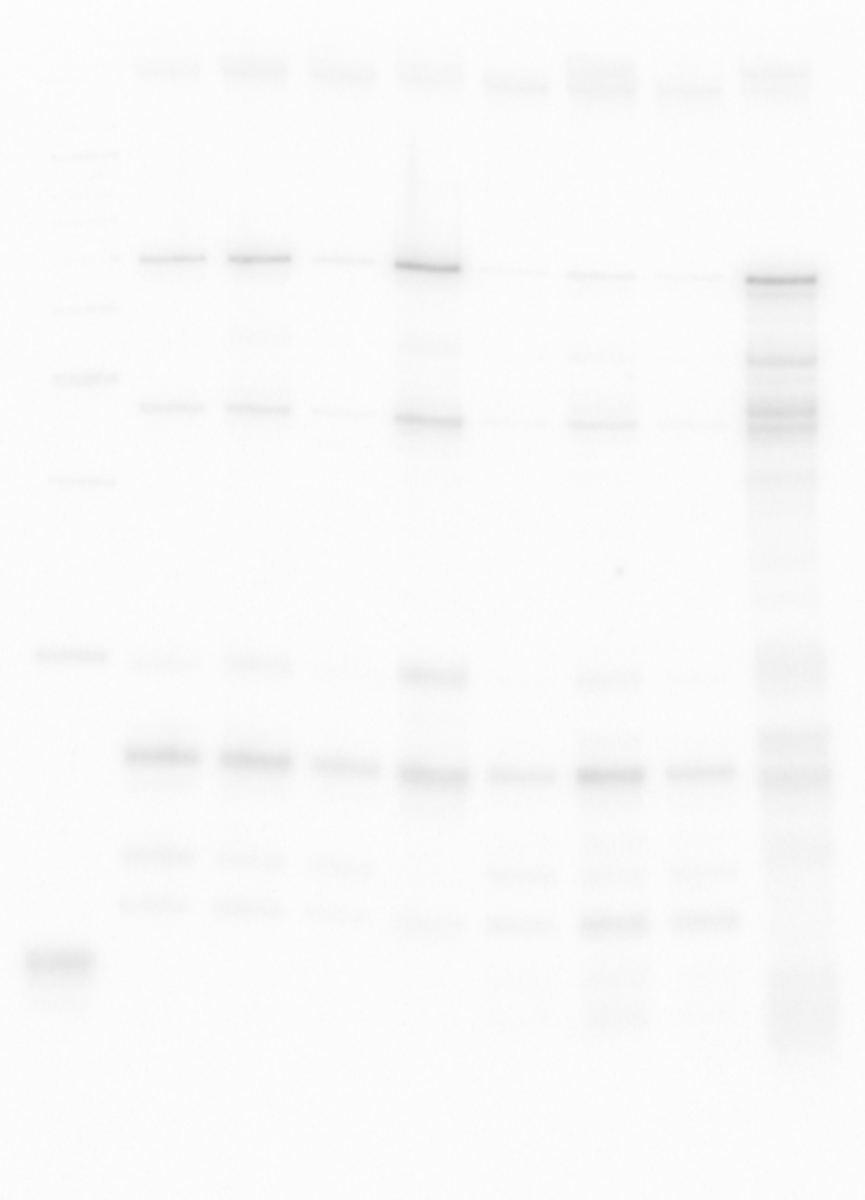


1 2 3 4 [lane]

Vcr082

5S

| **Northern blot** | **sRNA** | **probe** |
| --- | --- | --- |
| 8 | Vcr082 | KPO-2040 |

1 2 3 4 [lane]


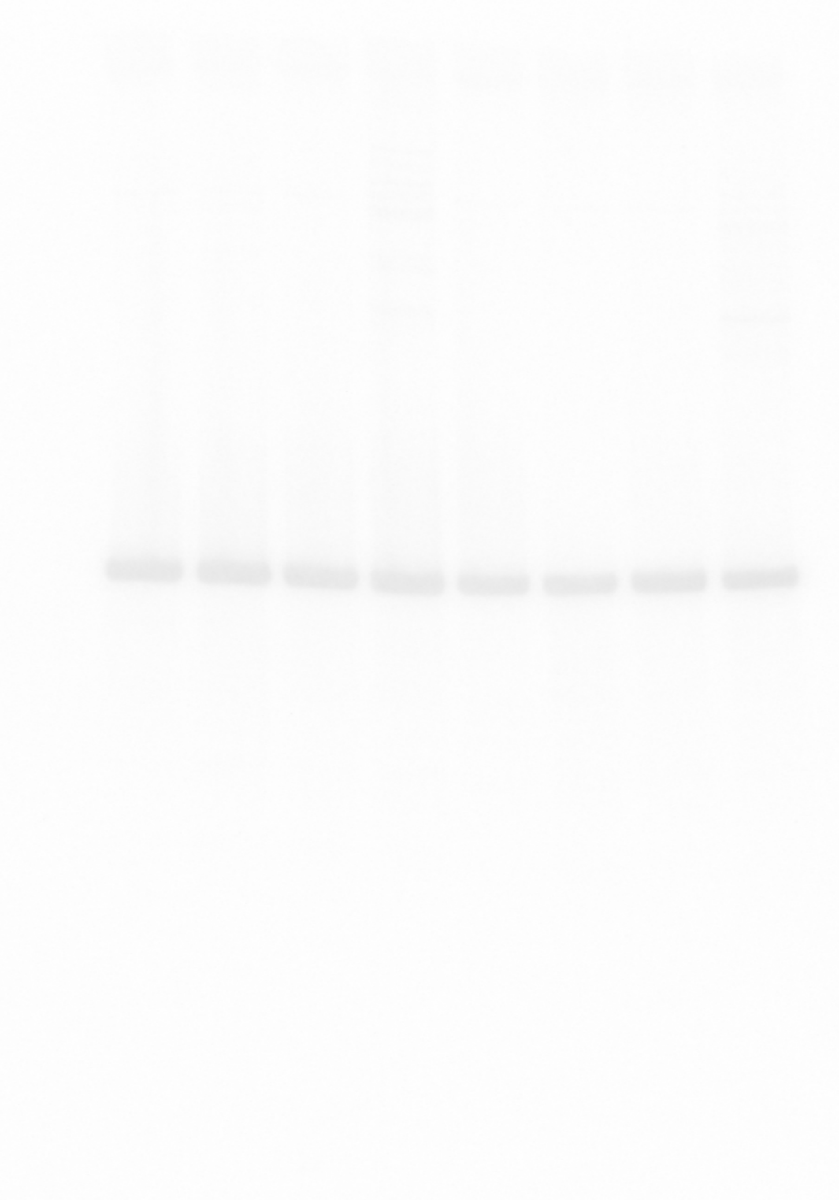

Supplement: Figure 1—figure supplement 5—source data 1. [file elife-58836-fig1-figsupp5-data1.docx]
