# Supplementary material for: Gene autoregulation by 3’ UTR-derived bacterial small RNAs
Source: eLife. 2020 Aug 3;9:e58836. doi: 10.7554/eLife.58836 (PMC7398697; doi:10.7554/eLife.58836)
Supplement: Figure 1—figure supplement 6—source data 1. [file elife-58836-fig1-figsupp6-data1.docx]

Source data for Figure 1 – figure supplement 6 Figure 1 – figure supplement 6


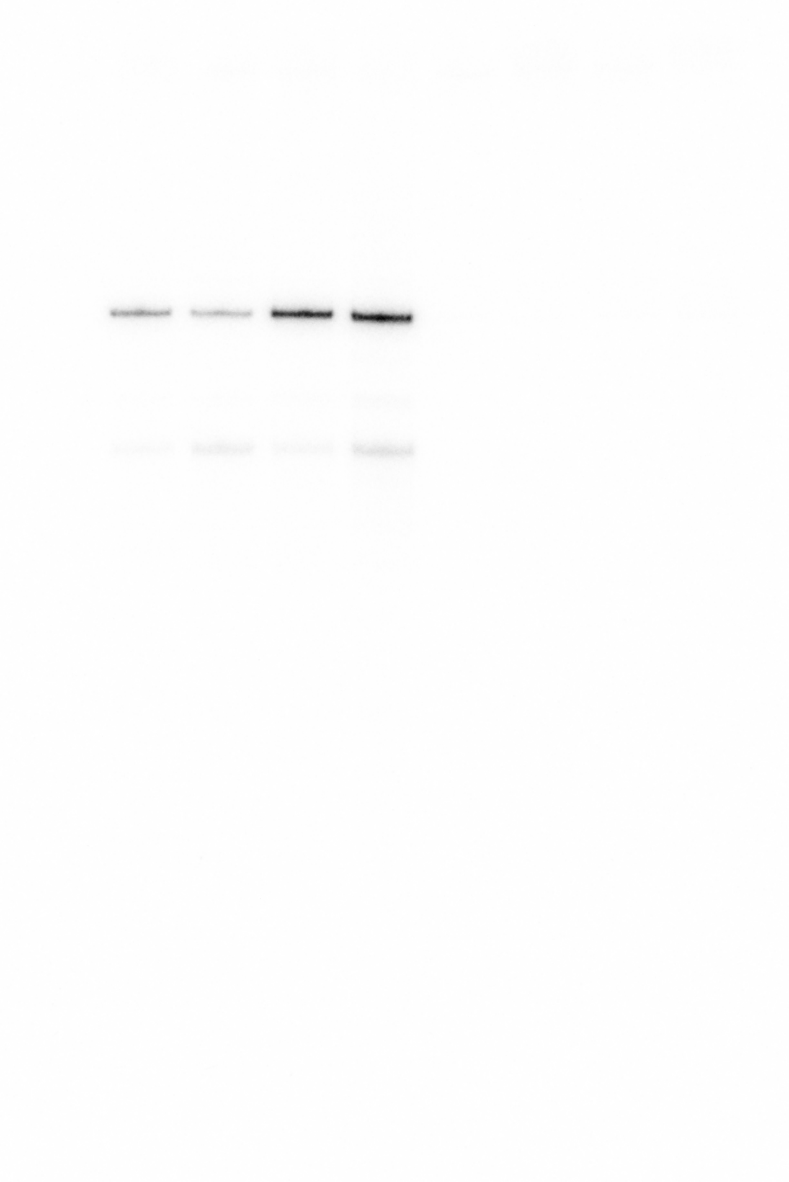


1 2 3 4 [lane]

RyhB


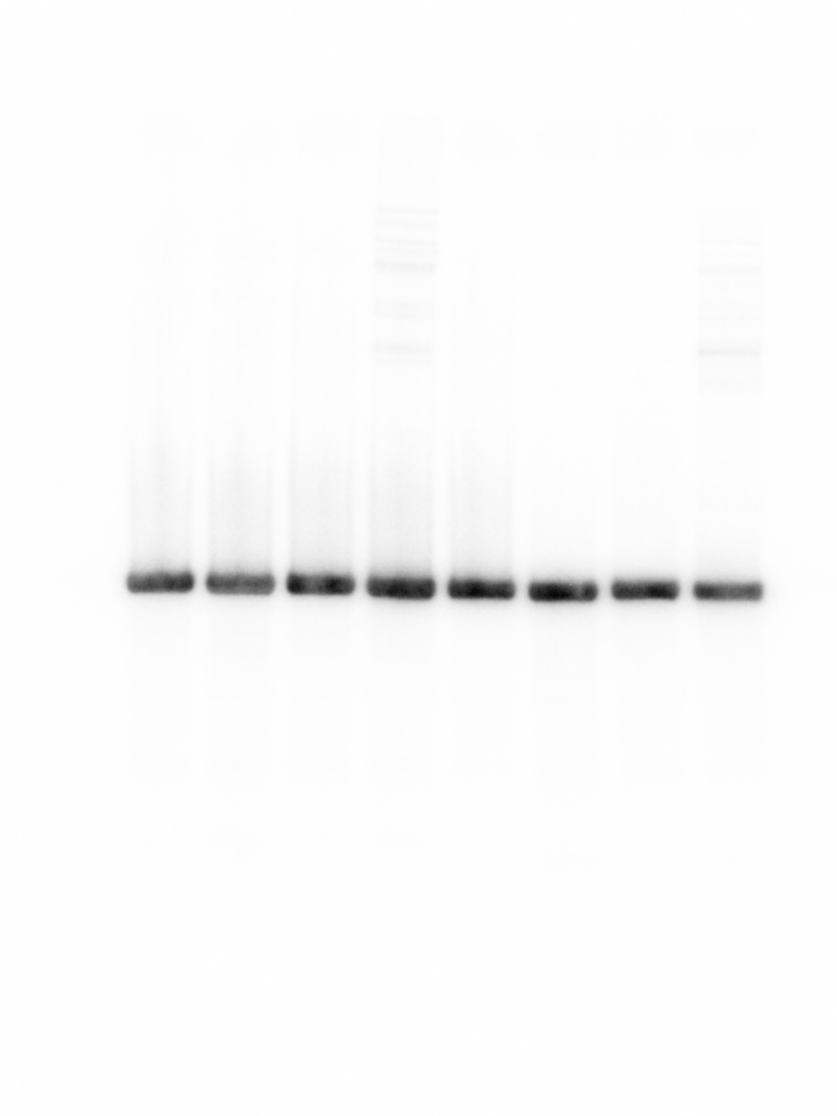


1 2 3 4 [lane]

| **Northern blot** | **sRNA** | **probe** |
| --- | --- | --- |
| 9 | RyhB | KPO-0597 |

5S

| **Northern blot** | **sRNA** | **probe** |
| --- | --- | --- |
| 10 | Spot 42 | KPO-3726 |


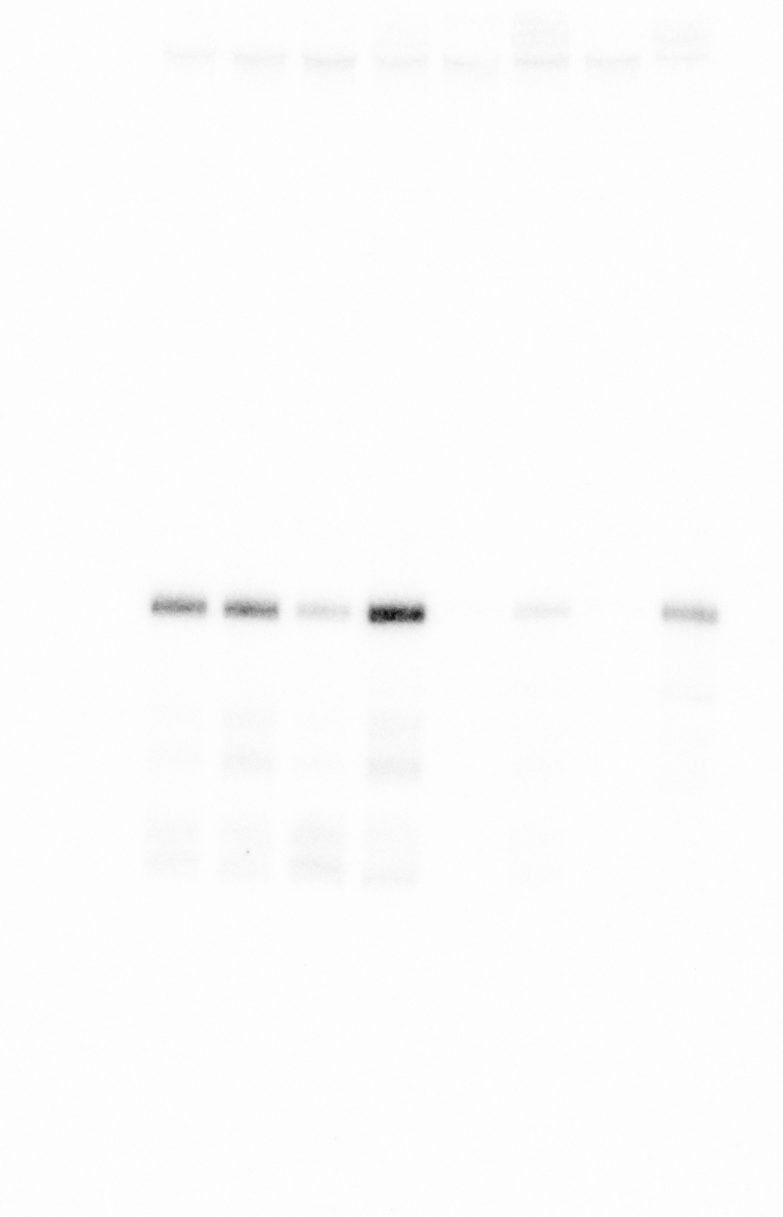


1 2 3 4 [lane]

Spot42


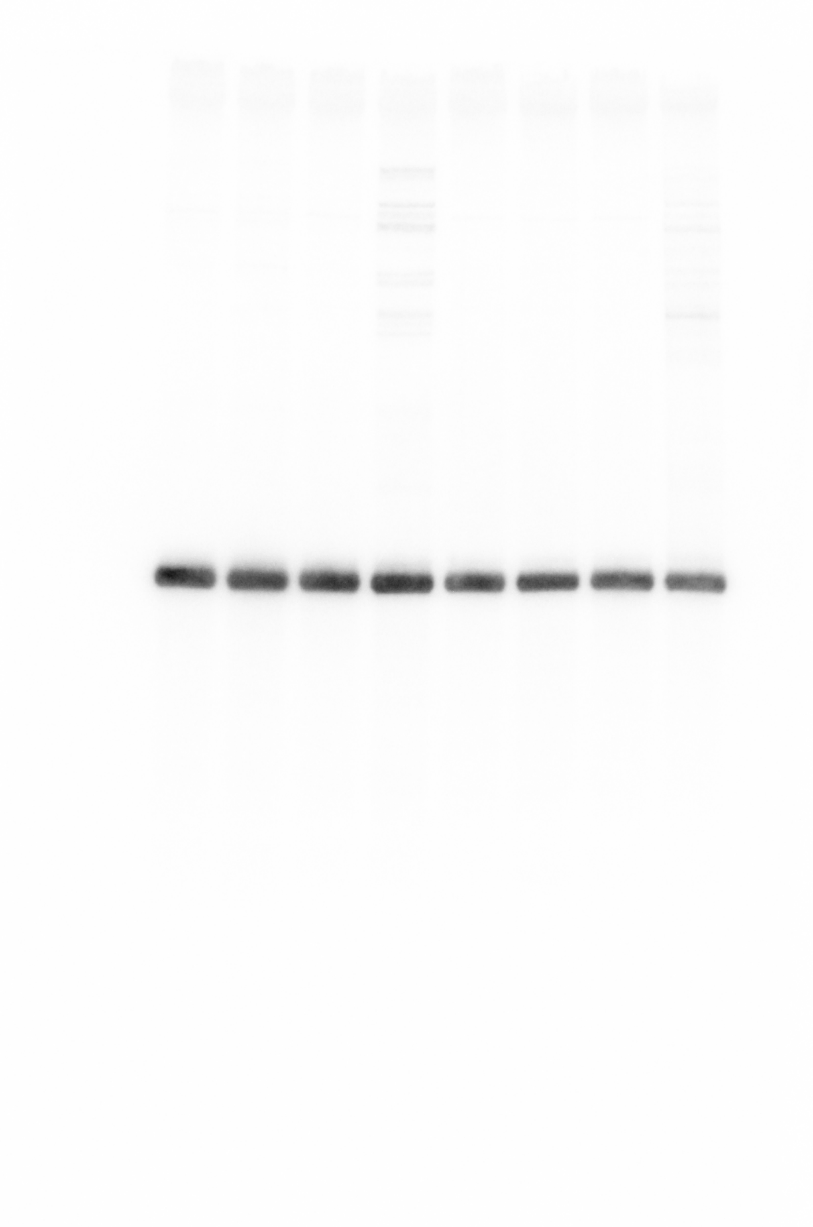


1 2 3 4 [lane]

5S

Figure 1 – figure supplement 6

| **Northern blot** | **sRNA** | **probe** |
| --- | --- | --- |
| 8 | VqmR | KPO-0452 |

1 2 3 4 [lane]


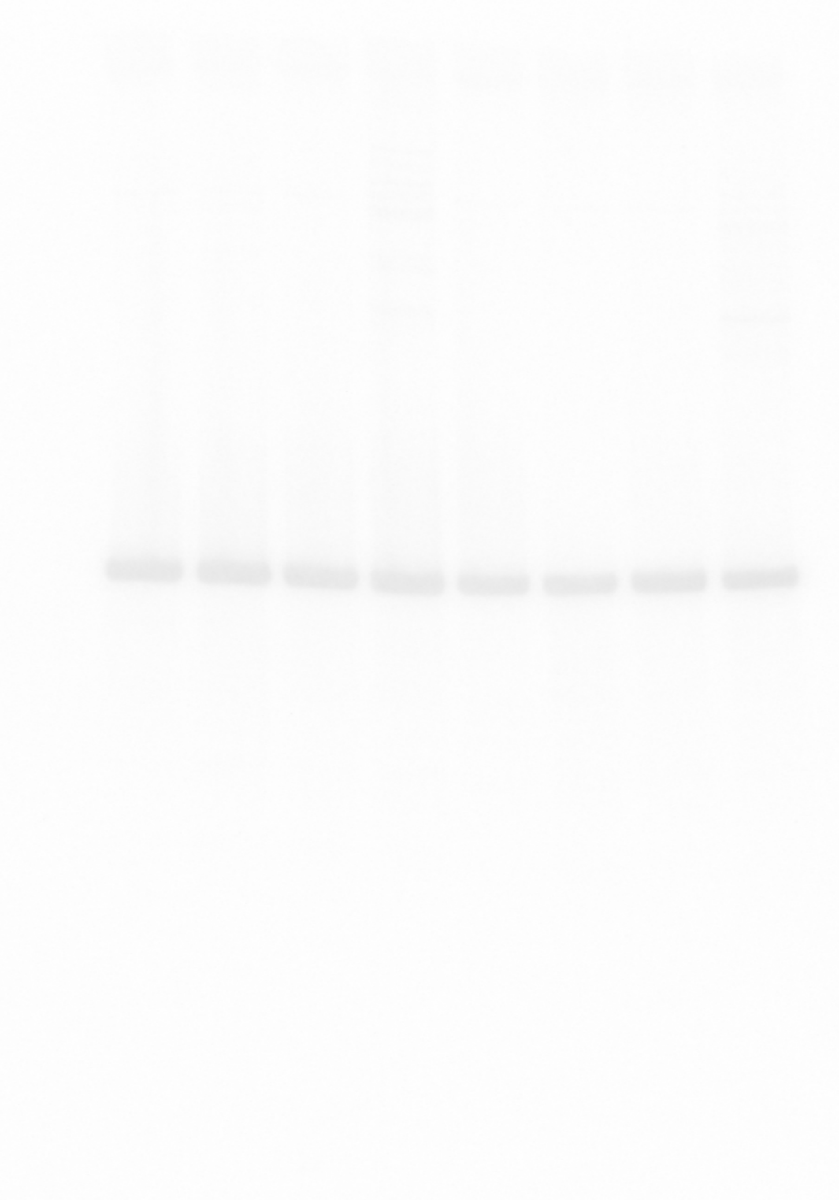


5S


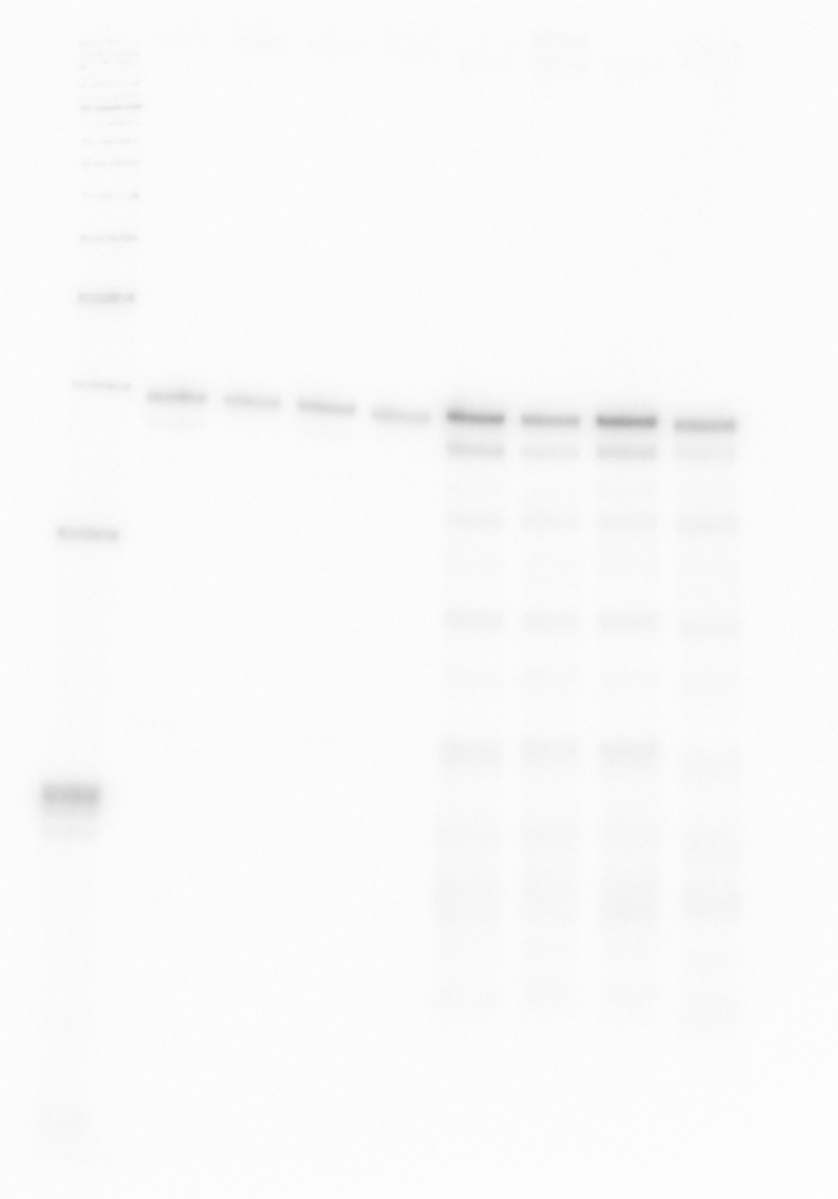


1 2 3 4 [lane]

VqmR
