# Supplementary material for: Gene autoregulation by 3’ UTR-derived bacterial small RNAs
Source: eLife. 2020 Aug 3;9:e58836. doi: 10.7554/eLife.58836 (PMC7398697; doi:10.7554/eLife.58836)
Supplement: Figure 2—source data 1. [file elife-58836-fig2-data1.docx]

# Figure 2B

1 2 3 4 [lane]


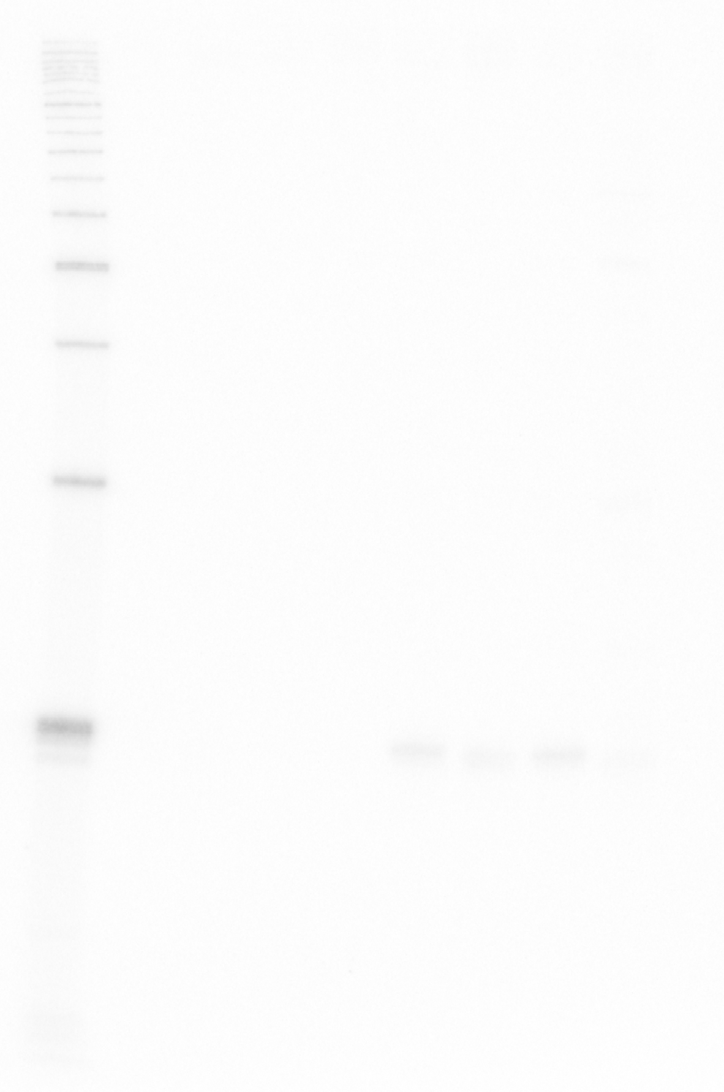

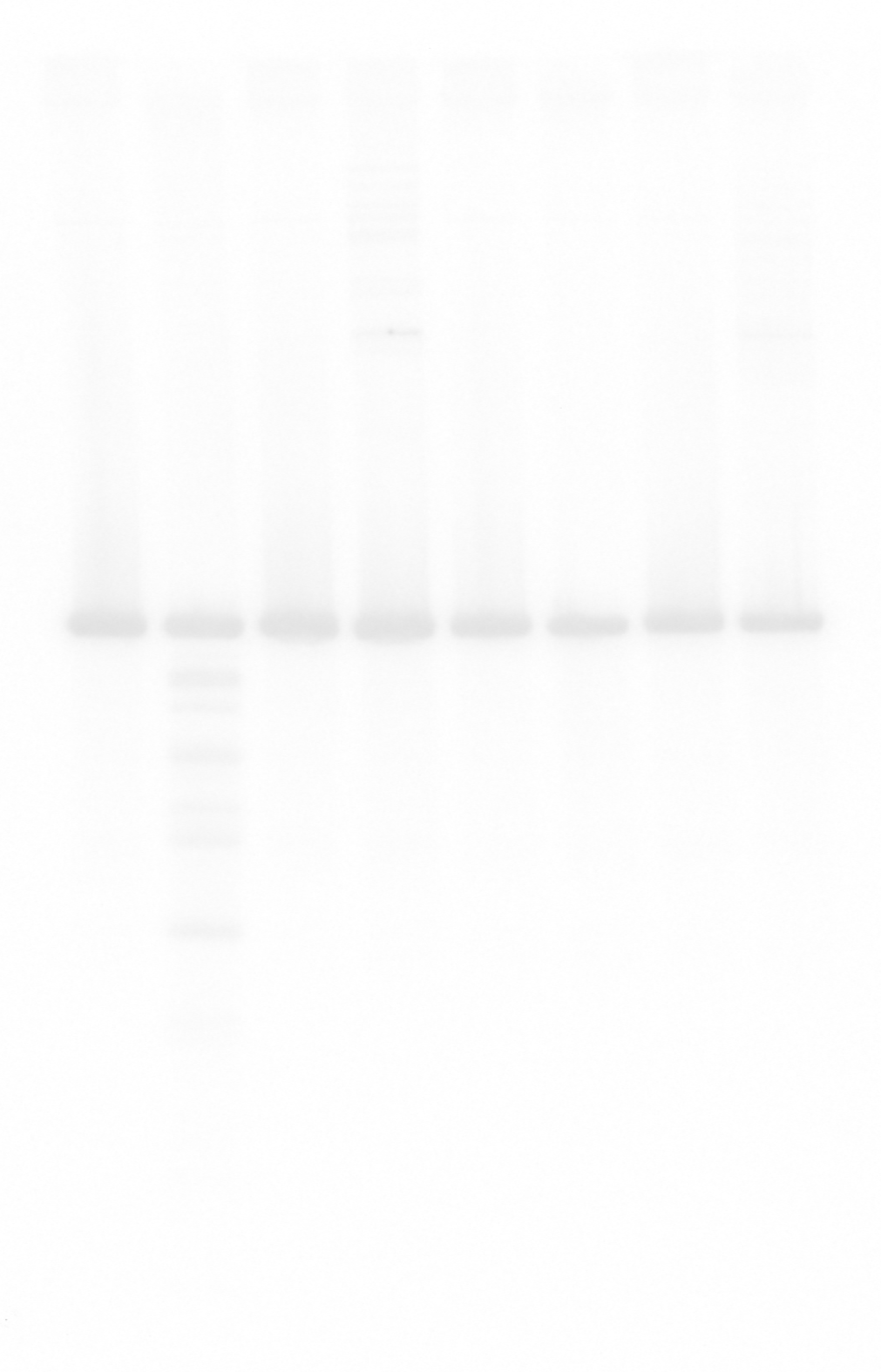


1 2 3 4 [lane]

OppZ (KPO-0845) 5S (KPO-0243)


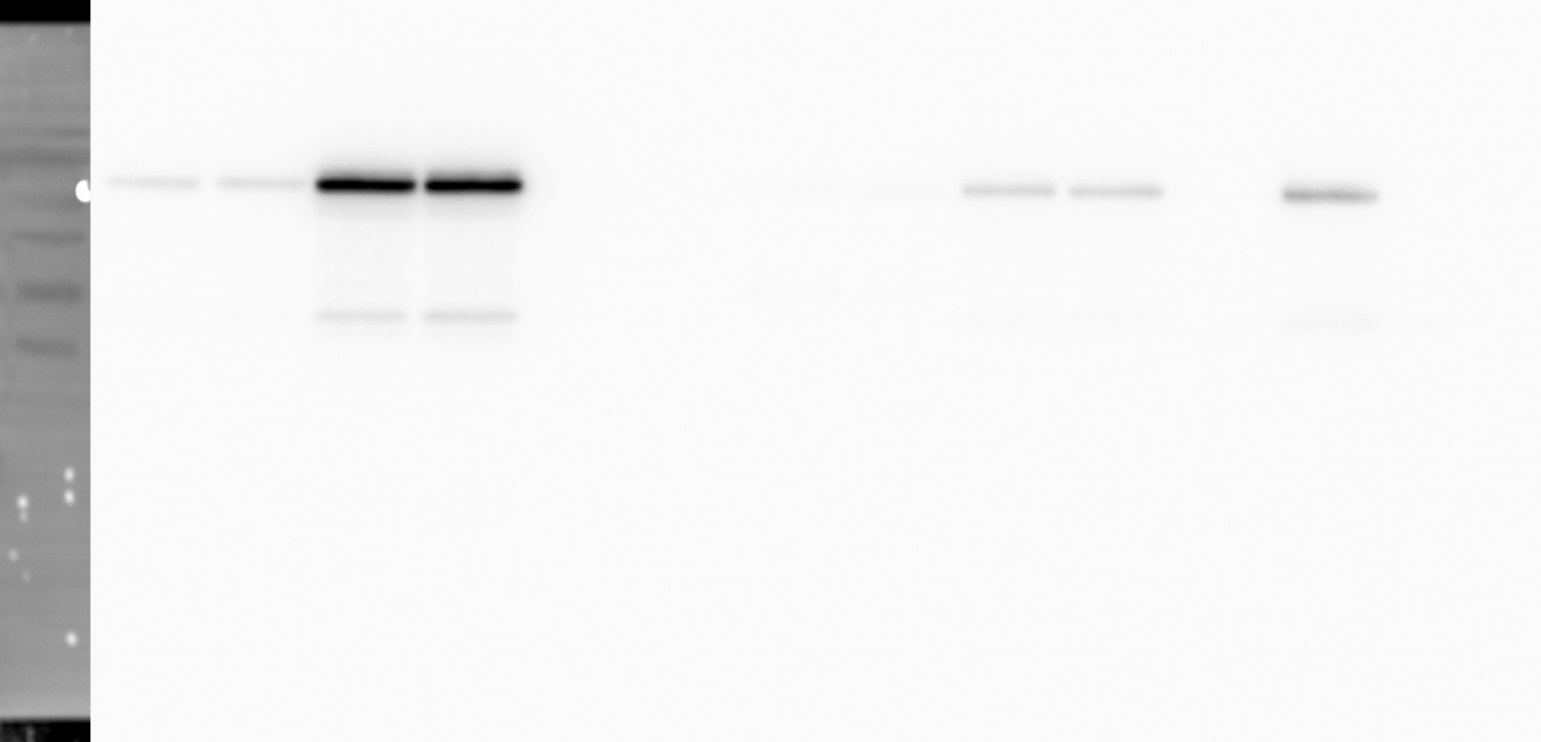


kDa

70

55

40

35

25

OppA

OppB

α-FLAG


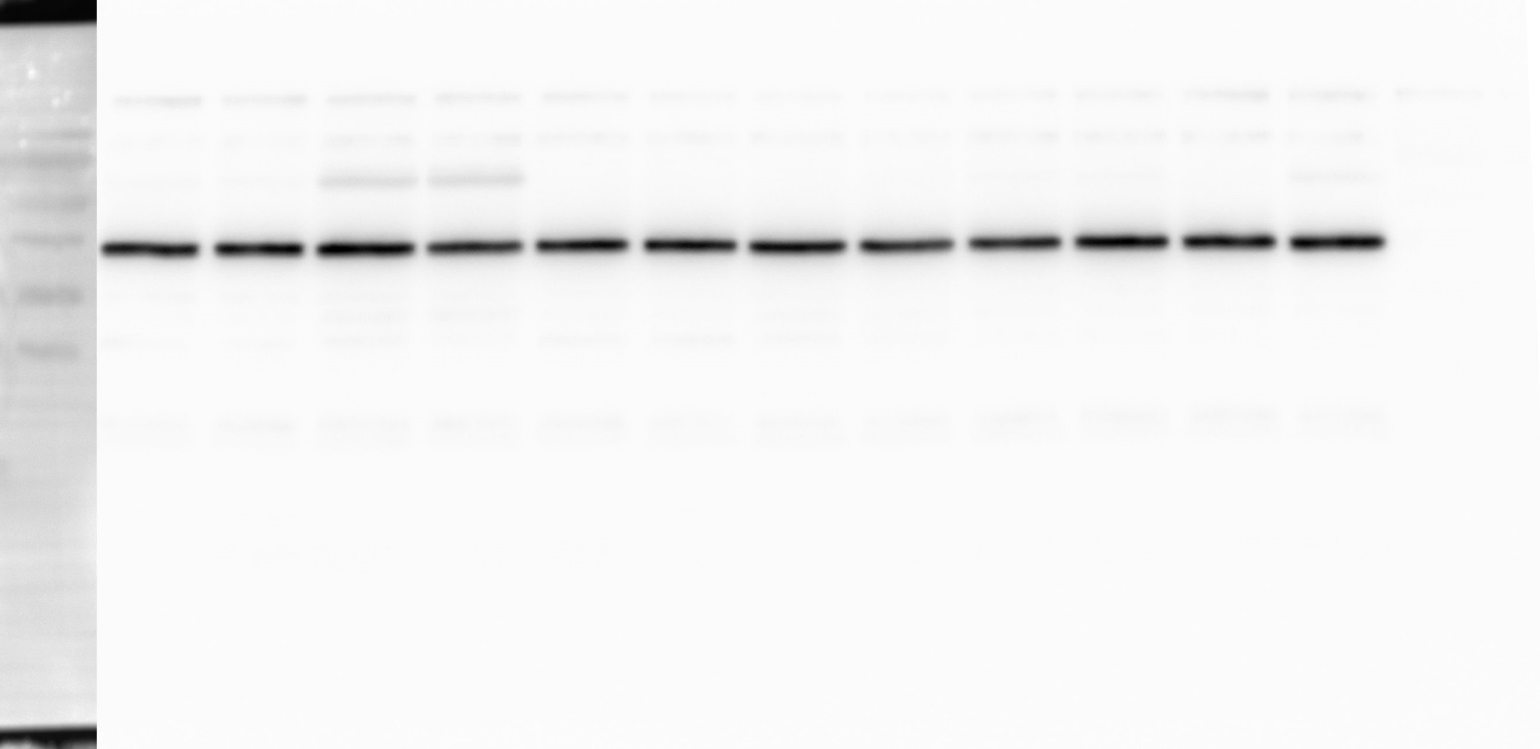


kDa

70

55

40

35

25

RNAP

α-RNAP

5S (KPO-0243)


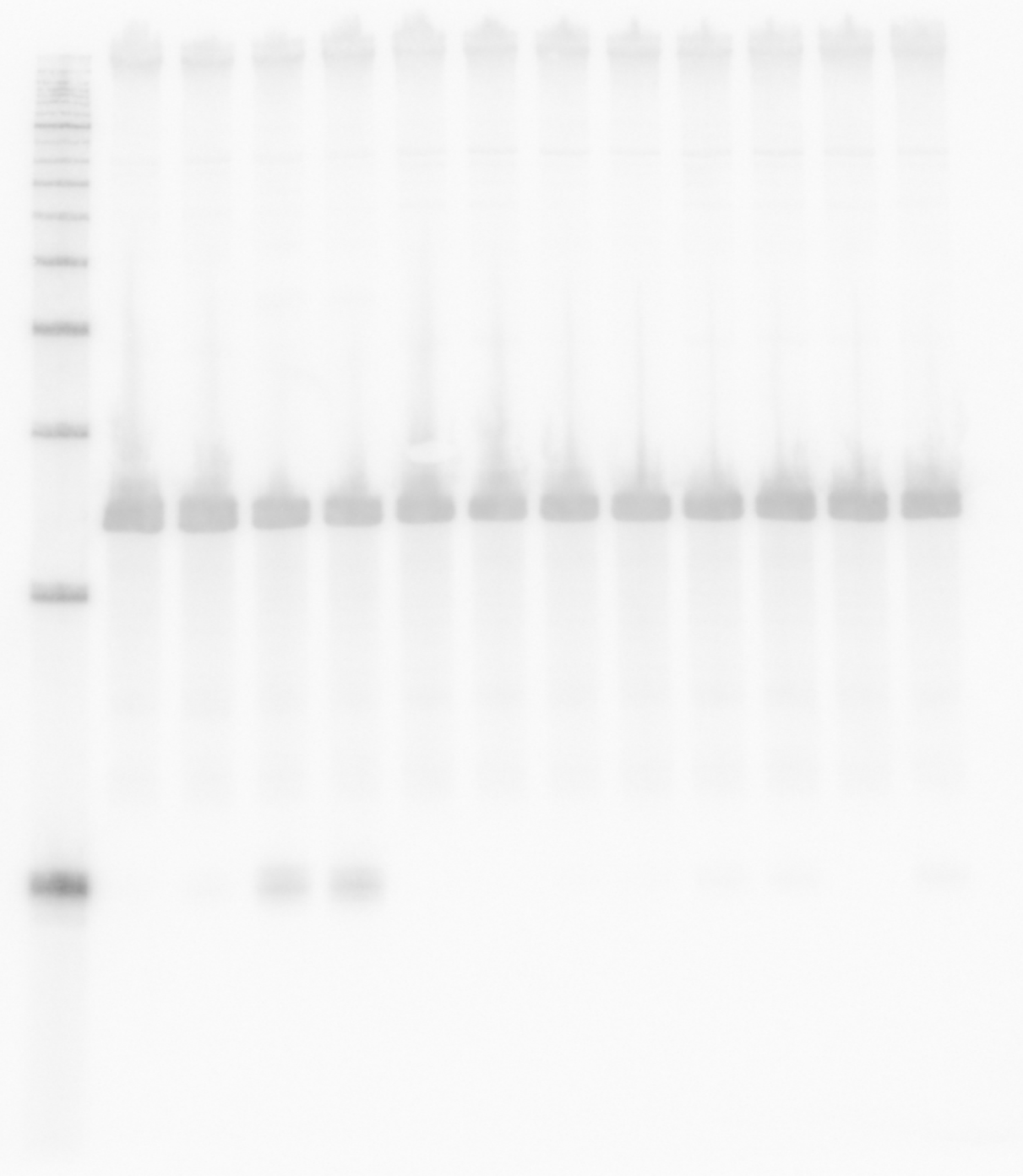


1 2 3 4 5 6 7 8 9 10 11 12 [lane]

O

ppZ (KPO-0845)

1 2 3 4 [lane]


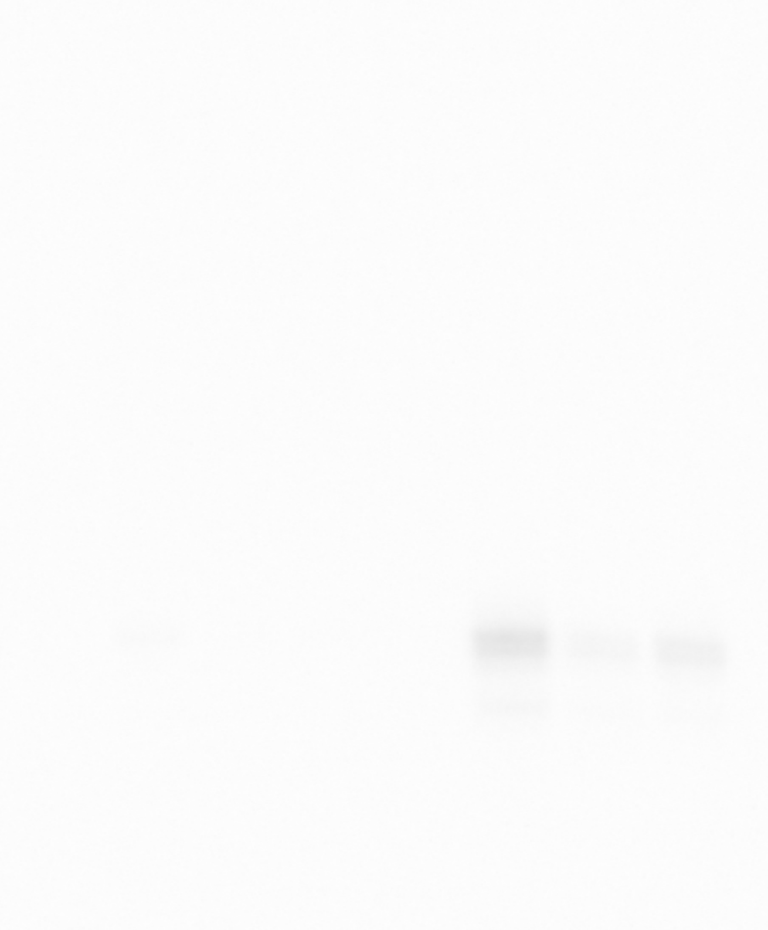

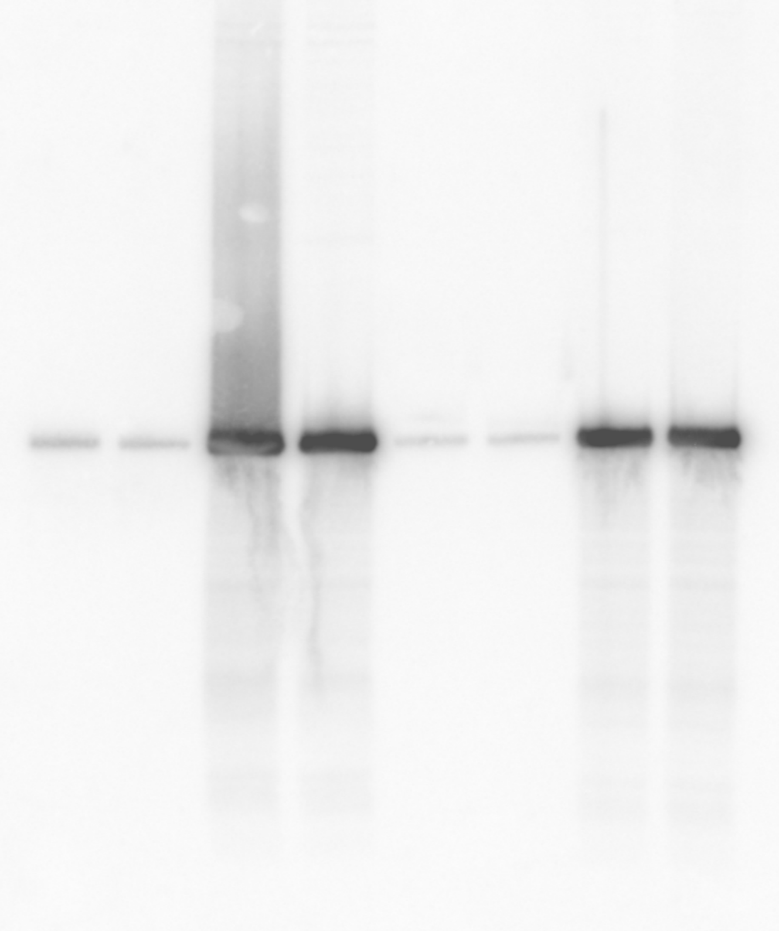


1 2 3 4 [lane]

OppZ (KPO-0845) 5S (KPO-0243)
