# Supplementary figures and images for: Gene autoregulation by 3’ UTR-derived bacterial small RNAs
Source: eLife. 2020 Aug 3;9:e58836. doi: 10.7554/eLife.58836 (PMC7398697; doi:10.7554/eLife.58836)

Source data for Figure 2 – figure supplement 1

Figure 2 – figure supplement 1B


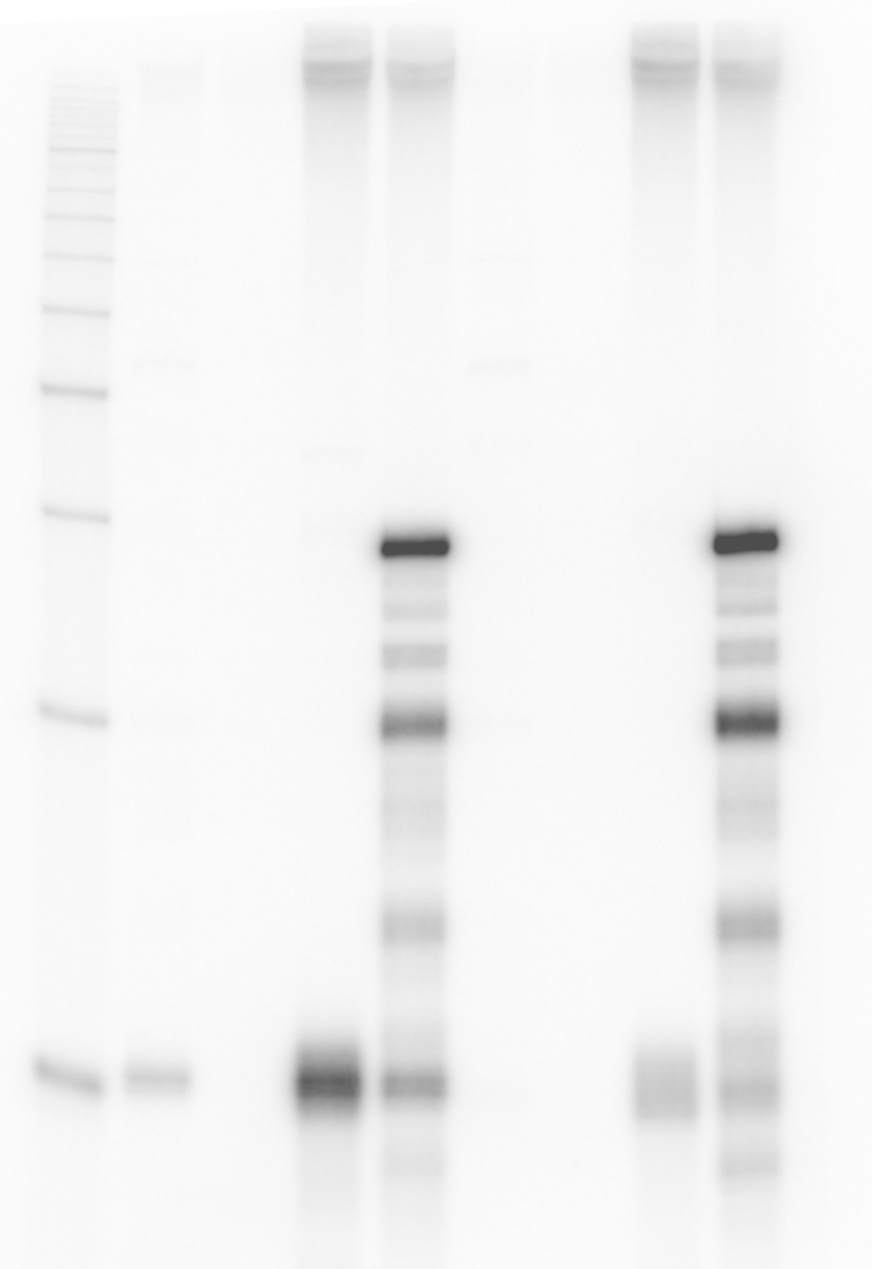


1 2 3 4 5 6 7 8 [lane]


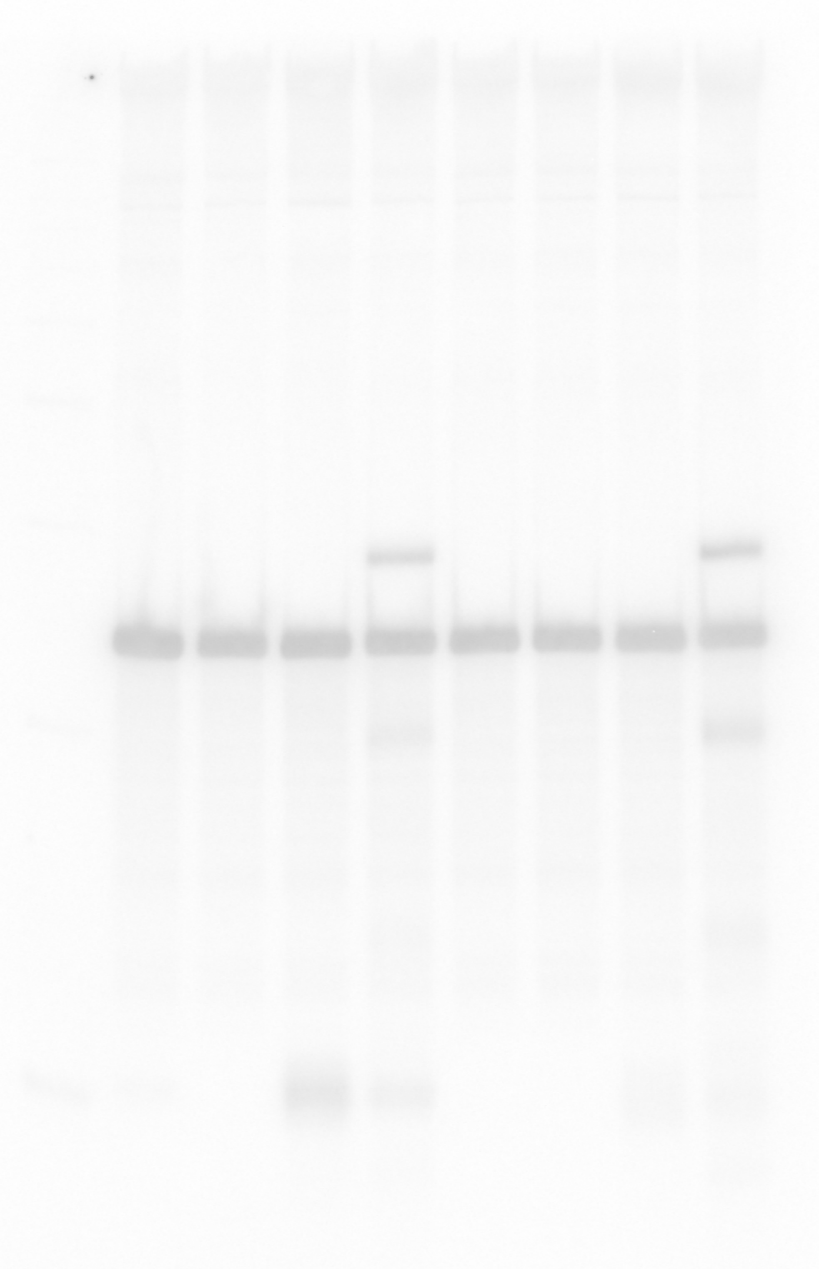


1 2 3 4 5 6 7 8 [lane]

OppZ (KPO-2687) 5S (KPO-0243)

Supplement: Figure 2—figure supplement 1—source data 1. [file elife-58836-fig2-figsupp1-data1.docx]

Source data for Figure 3 – figure supplement 3


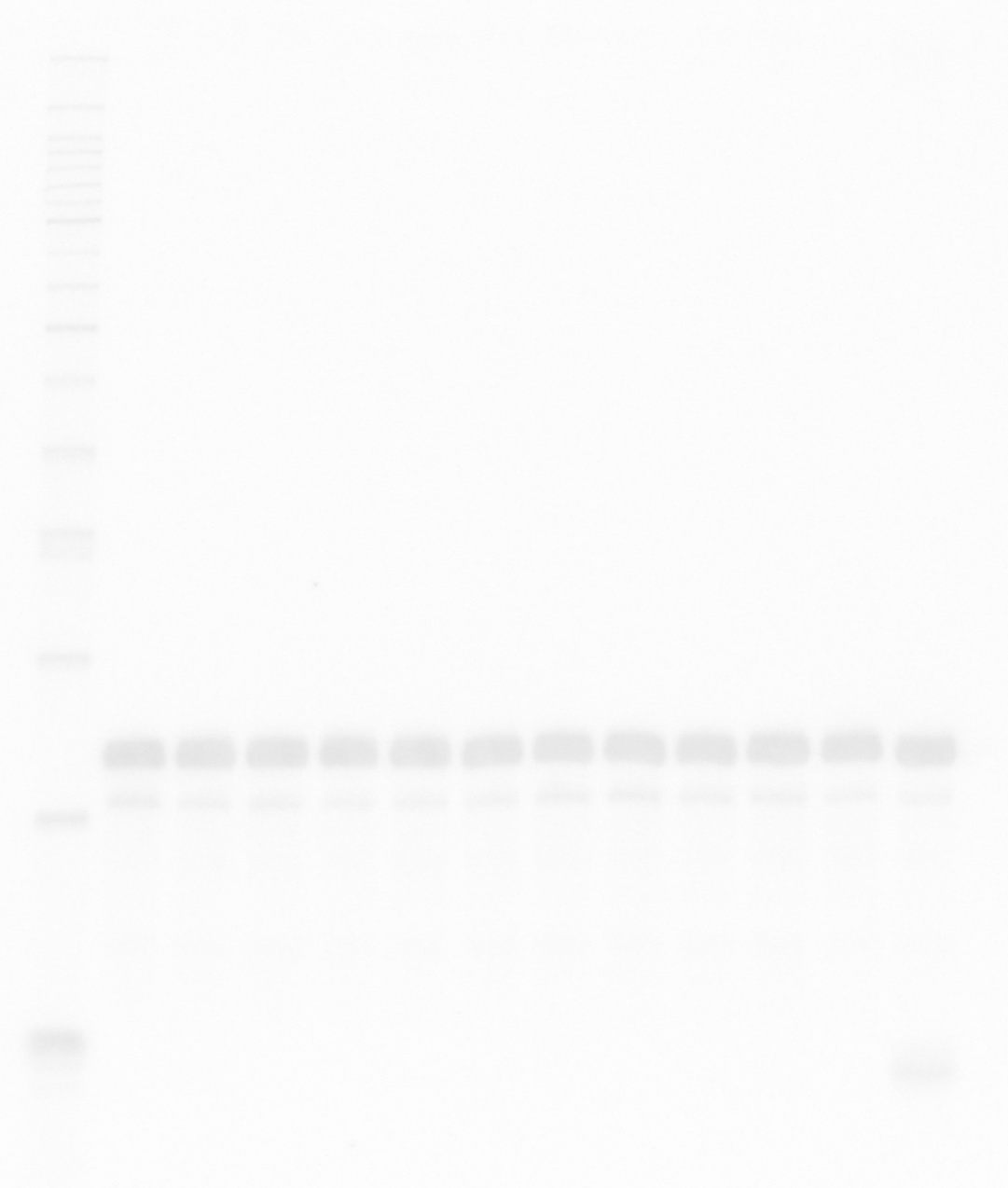


1 2 3 [lane]

Figure 3 – figure supplement 3B


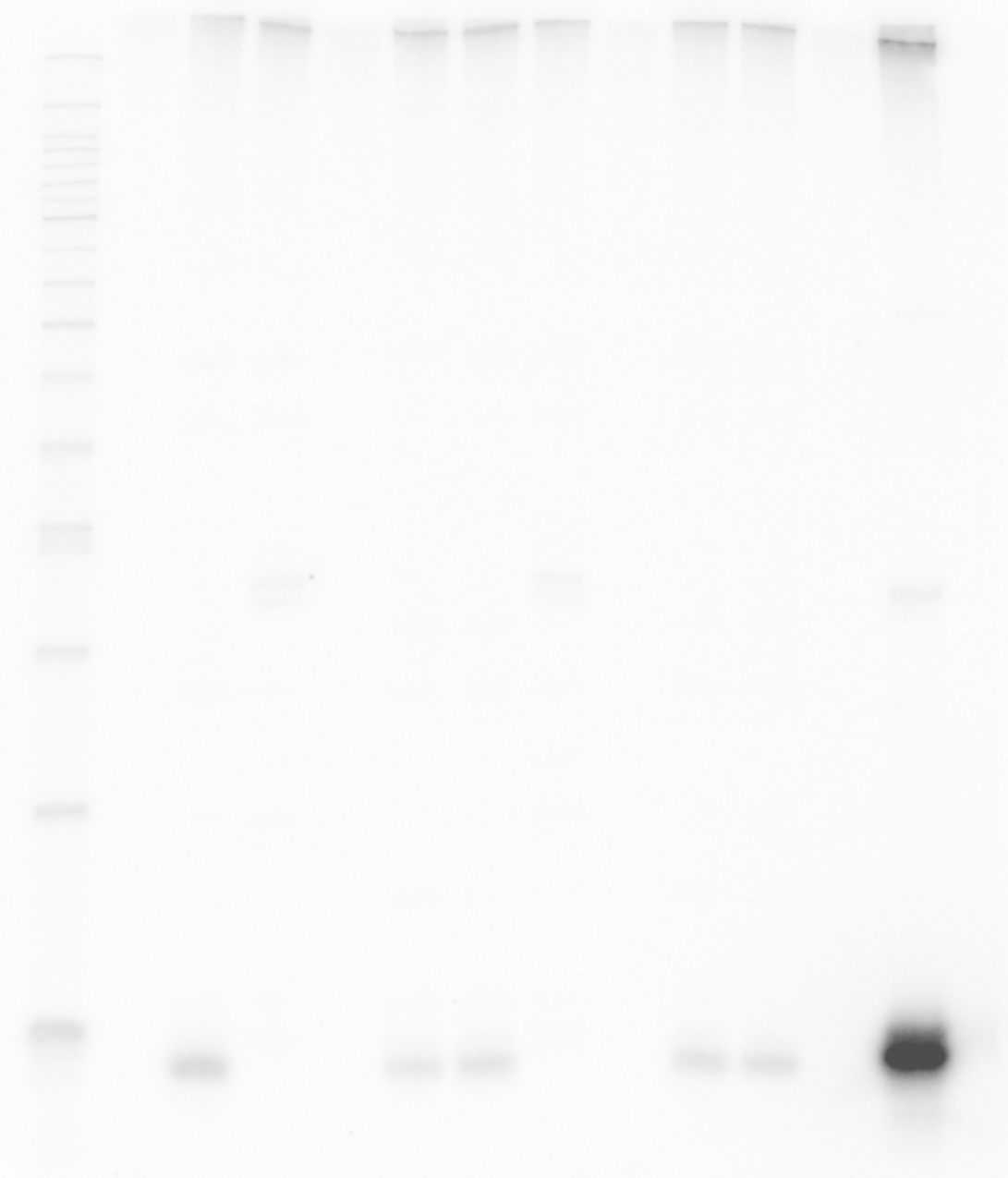


1 2 3 [lane]

OppZ (KPO-2687) 5S (KPO-0243)

Supplement: Figure 3—figure supplement 3—source data 1. [file elife-58836-fig3-figsupp3-data1.docx]

**Source data for Figure 6 Figure 6B**

kDa 70


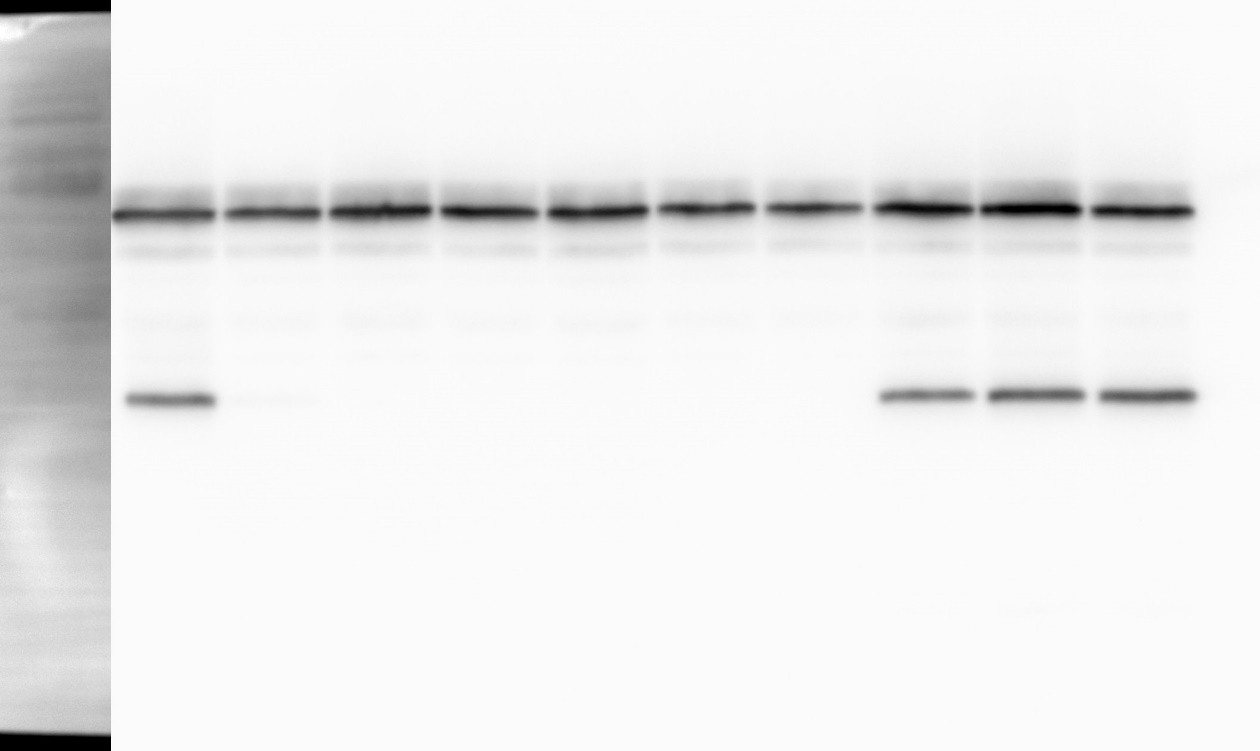


55

40

35

25

1 2 3 4 5 6 7 8 9 10 [lane]

OppA

OppB

α-FLAG


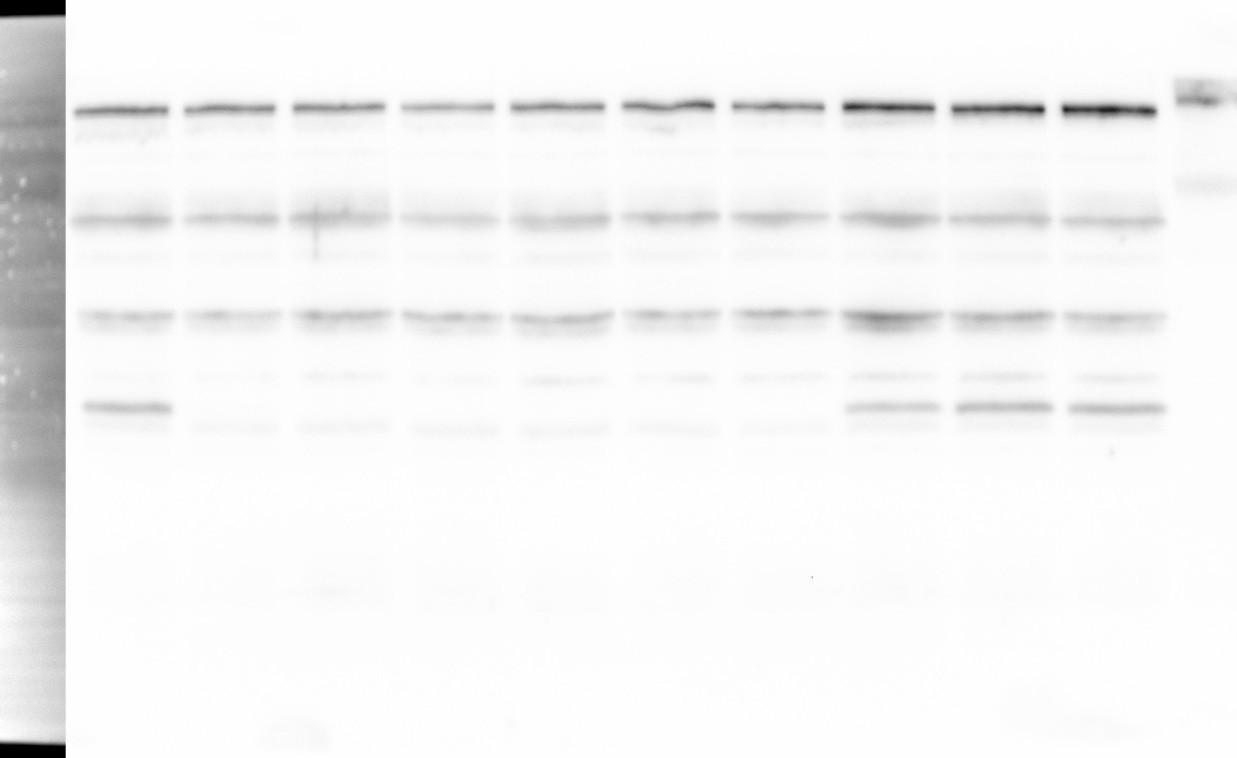


kDa 70

55

40

35

25

RNAP

α-RNAP


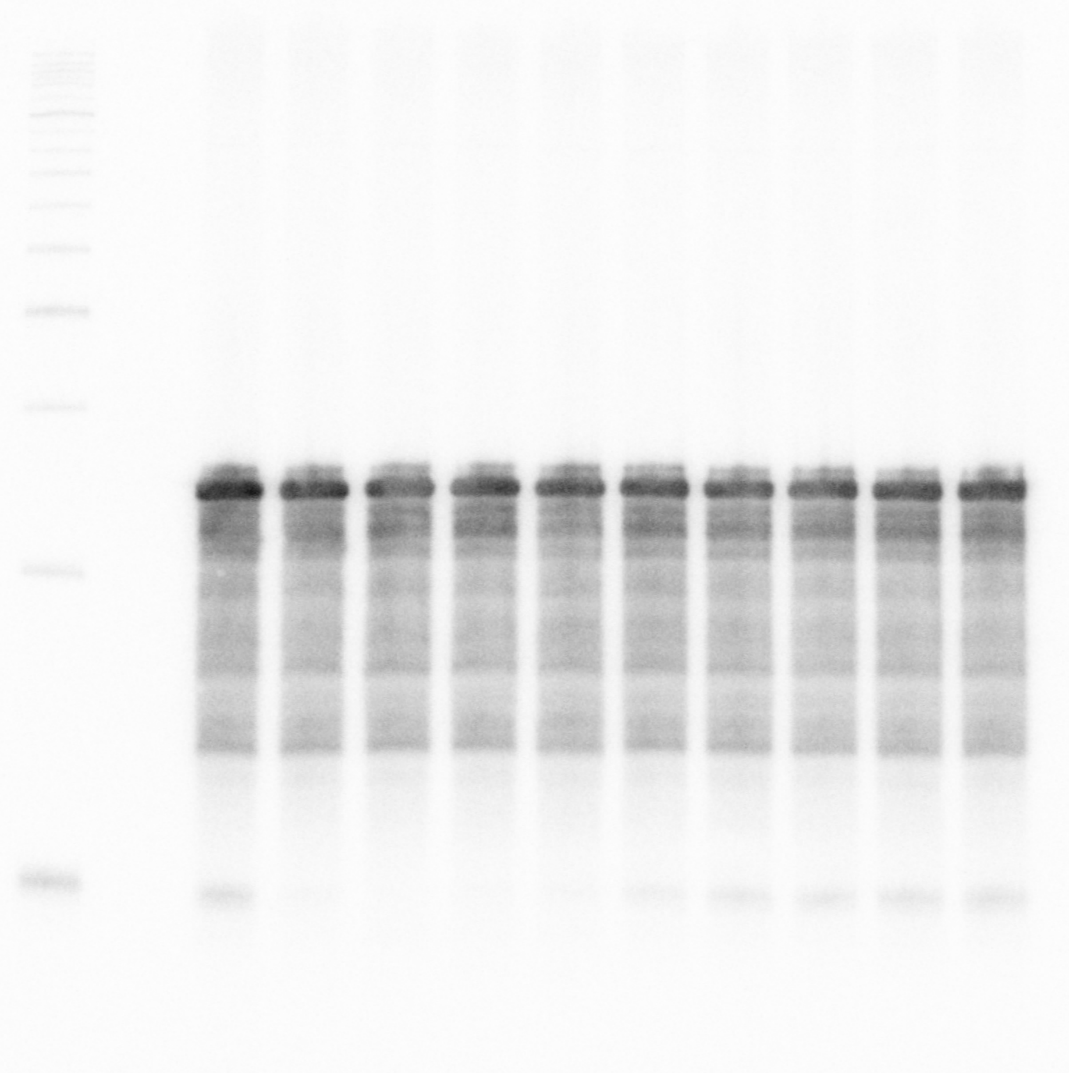


1 2 3 4 5 6 7 8 9 10 [lane]


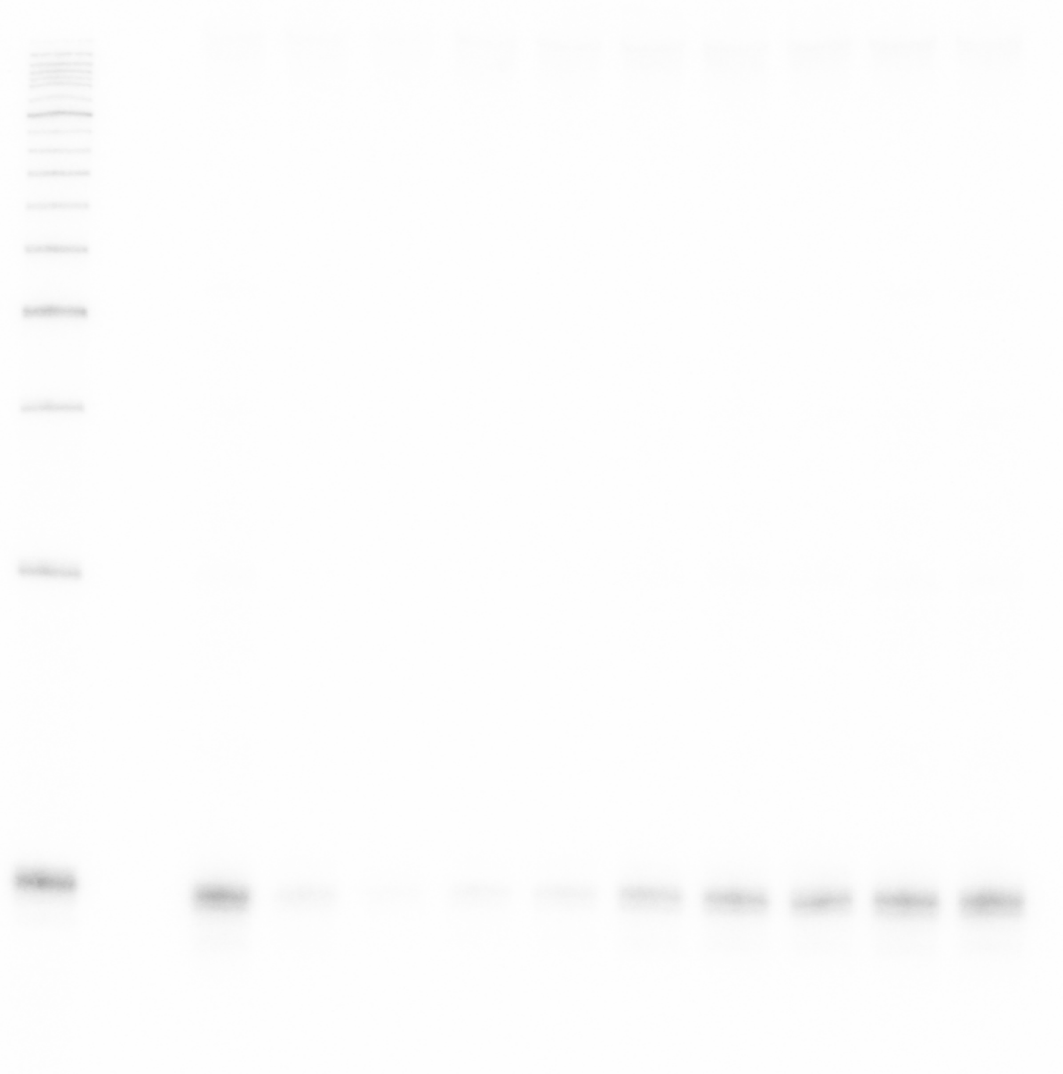


1 2 3 4 5 6 7 8 9 10 [lane]

OppZ (KPO-0845) 5S (KPO-0243)

Supplement: Figure 6—source data 1. [file elife-58836-fig6-data1.docx]
