# Supplementary material for: Gene autoregulation by 3’ UTR-derived bacterial small RNAs
Source: eLife. 2020 Aug 3;9:e58836. doi: 10.7554/eLife.58836 (PMC7398697; doi:10.7554/eLife.58836)
Supplement: Figure 2—figure supplement 2—source data 1. [file elife-58836-fig2-figsupp2-data1.docx]

# Source data for Figure 2 – figure supplement 2

**Figure 2 – figure supplement 2**

Data:

- OppZ and 5S quantified from three biological replicates on Northern blots
- OppZ normalized to 5S levels
- Fold change relative to t = 0 min

| **strain** | **min** | **rep 1** | **rep 2** | **rep 3** | **mean** | **SD** |
| --- | --- | --- | --- | --- | --- | --- |
| **WT** | 0 | 1.0000 | 1.0000 | 1.0000 | 1.0000 | 0.0000 |
|  | 2 | 0.9989 | 1.8421 | 1.1029 | 1.3147 | 0.3754 |
|  | 4 | 1.7584 | 1.0207 | 0.9059 | 1.2283 | 0.3777 |
|  | 8 | 1.9053 | 1.2524 | 0.8635 | 1.3404 | 0.4298 |
|  | 16 | 1.4940 | 1.5745 | 0.8445 | 1.3043 | 0.3268 |
|  | 32 | 1.4852 | 1.0616 | 0.6744 | 1.0737 | 0.3311 |
| **Δ*hfq*** | 0 | 1.0000 | 1.0000 | 1.0000 | 1.0000 | 0.0000 |
|  | 2 | 1.2078 | 0.8839 | 0.7603 | 0.9506 | 0.1887 |
|  | 4 | 1.3199 | 1.0816 | 0.9259 | 1.1091 | 0.1620 |
|  | 8 | 0.8122 | 1.0667 | 0.7236 | 0.8675 | 0.1454 |
|  | 16 | 0.5193 | 0.3877 | 0.6019 | 0.5030 | 0.0882 |
|  | 32 | 0.1547 | 0.0000 | 0.2446 | 0.1331 | 0.1010 |
