# Supplementary material for: Gene autoregulation by 3’ UTR-derived bacterial small RNAs
Source: eLife. 2020 Aug 3;9:e58836. doi: 10.7554/eLife.58836 (PMC7398697; doi:10.7554/eLife.58836)
Supplement: Figure 3—source data 1. [file elife-58836-fig3-data1.docx]

# Source data for Figure 3 Figure 3C


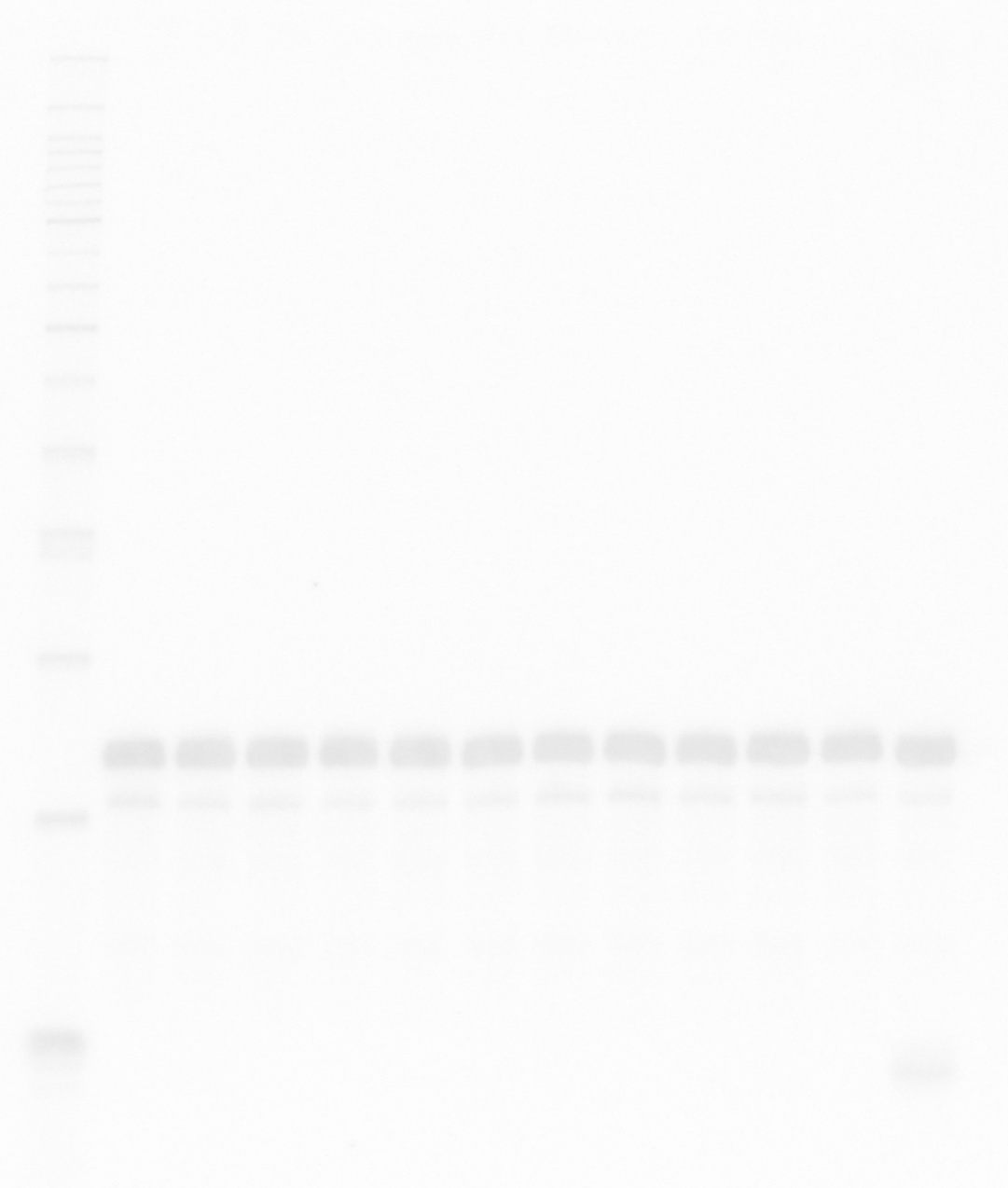


1 2 3 4 5 6 7 [lane]

Data: (fluorescence / OD600) - autofluorescence

|  |  | **GFP** | | | **mKate** | | |
| --- | --- | --- | --- | --- | --- | --- | --- |
| **target** | **sRNA** | **rep 1** | **rep 2** | **rep 3** | **rep 1** | **rep 2** | **rep 3** |
| oppB | pCtrl | 214821.225 | 222896.526 | 225483.774 | 244479.285 | 208228.068 | 254373.886 |
|  | pOppZ | 36993.6968 | 32668.3115 | 22104.5725 | 292132.404 | 290461.126 | 246929.724 |
|  | pOppZ M1 | 301983.259 | 309167.255 | 304122.279 | 310038.138 | 326541.874 | 283870.846 |
|  | pOppZ M2 | 69995.5568 | 68067.0223 | 71482.2319 | 335650.279 | 316804.109 | 327397.633 |
| oppB M1 | pCtrl | 258824.158 | 249376.83 | 264128.097 | 284191.375 | 270247.941 | 287480.077 |
|  | pOppZ | 243513.686 | 247494.309 | 249860.501 | 312764.722 | 286174.275 | 311146.673 |
|  | pOppZ M1 | 23159.5309 | 24740.9219 | 27431.4562 | 331955.362 | 338482.384 | 347508.692 |

# Figure 3D

Data: (fluorescence / OD600) - autofluorescence

| **5' end** | **3' UTR** | **rep 1** | **rep 2** | **rep 3** |
| --- | --- | --- | --- | --- |
| *oppB* | no *oppZ* | 134306.766 | 122162.299 | 119391.774 |
|  | *oppZ* | 76591.8146 | 87685.8367 | 75165.674 |
|  | *oppZ* M1 | 126353.86 | 123572.004 | 128291.904 |
|  | *oppZ* M2 | 160283.41 | 130466.274 | 143833.985 |
| *oppB* M1 | no *oppZ* | 125435.762 | 129691.884 | 125776.872 |
|  | *oppZ* | 140458.973 | 137865.096 | 137130.305 |
|  | *oppZ* M1 | 94553.8471 | 80045.6251 | 75825.2929 |


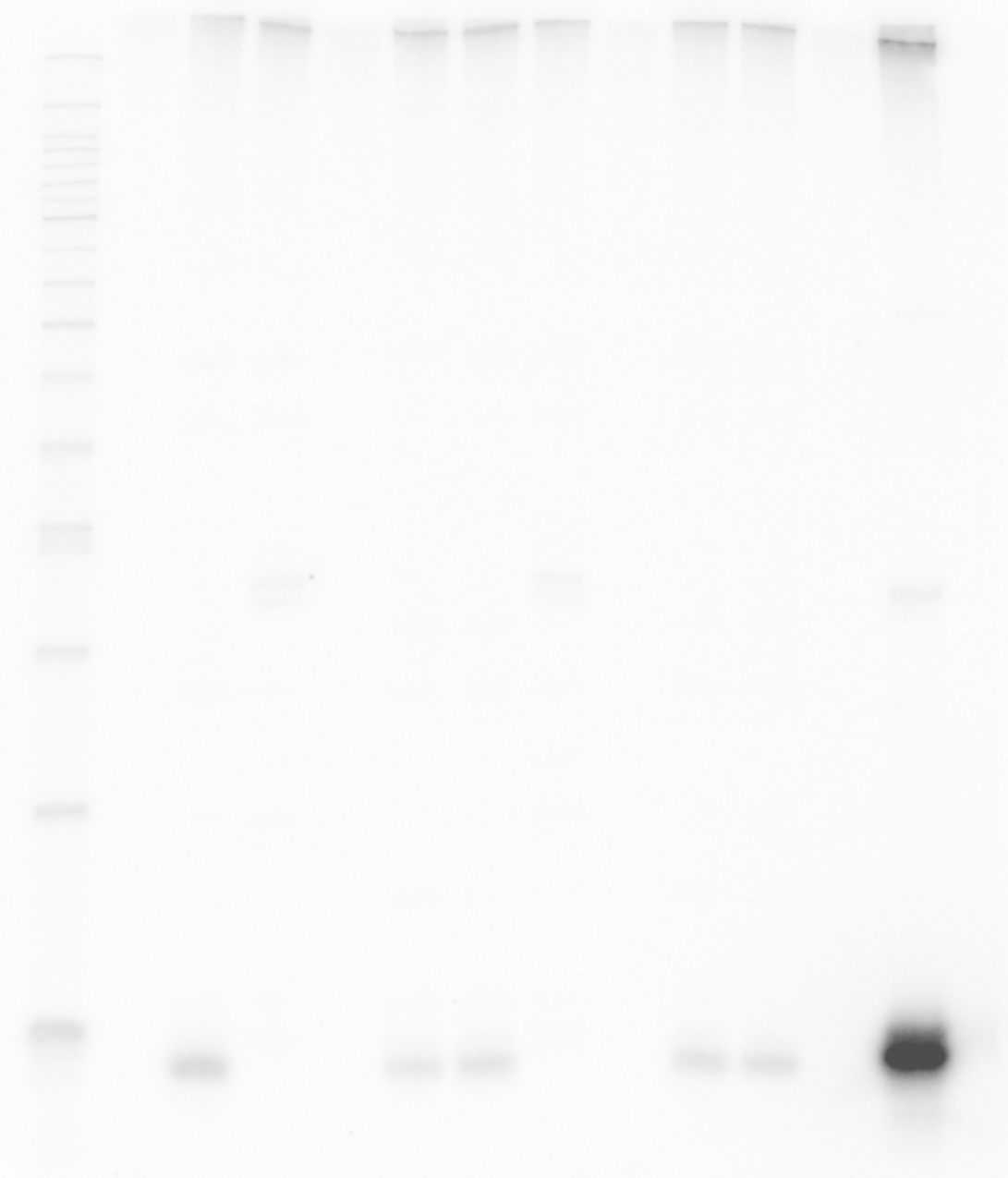


1 2 3 4 5 6 7 [lane]

OppZ (KPO-2687) 5S (KPO-0243)
