# Supplementary material for: Gene autoregulation by 3’ UTR-derived bacterial small RNAs
Source: eLife. 2020 Aug 3;9:e58836. doi: 10.7554/eLife.58836 (PMC7398697; doi:10.7554/eLife.58836)
Supplement: Figure 3—figure supplement 1—source data 1. [file elife-58836-fig3-figsupp1-data1.docx]

# Source data for Figure 3 – figure supplement 1 Figure 3 – figure supplement 1A


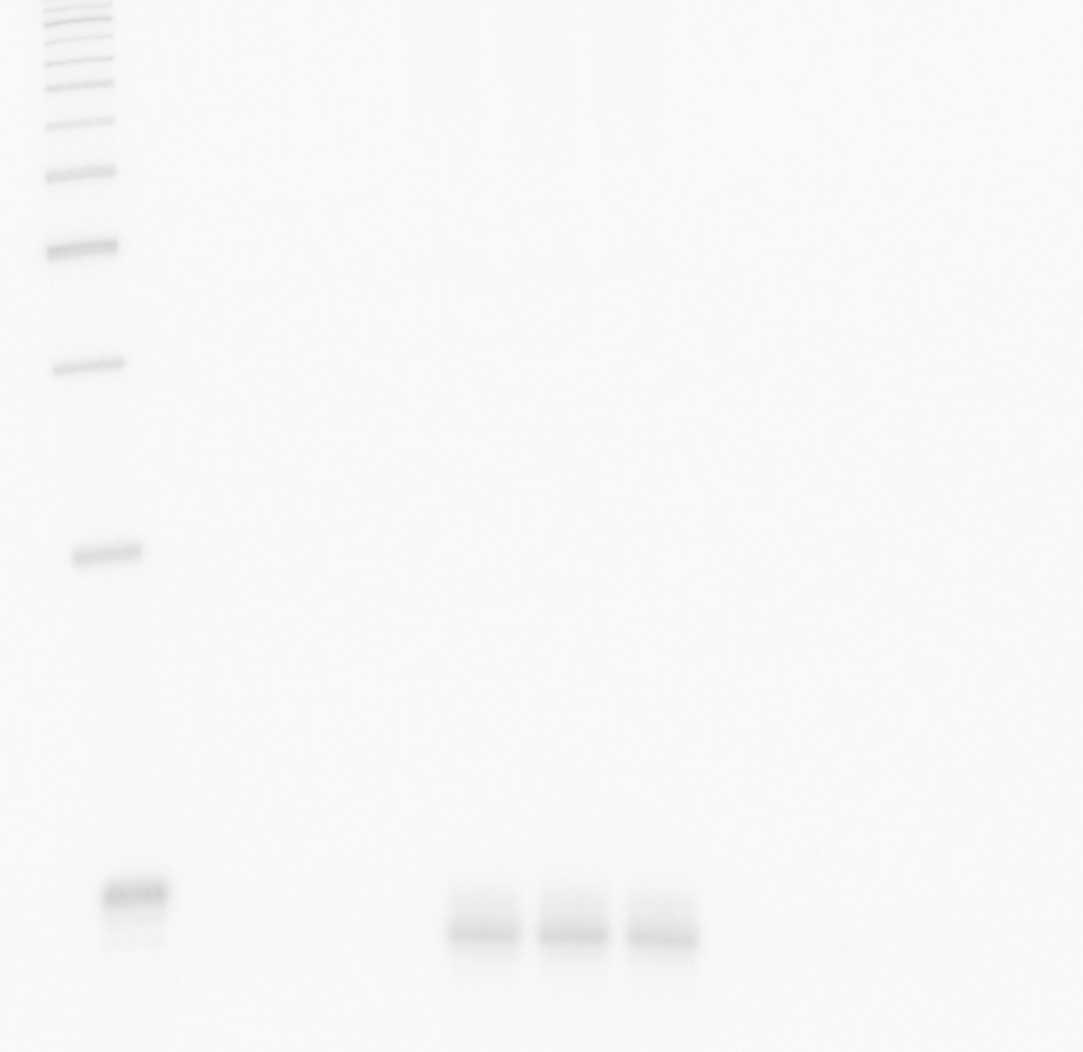


1 2 3 4 5 6 [lane]


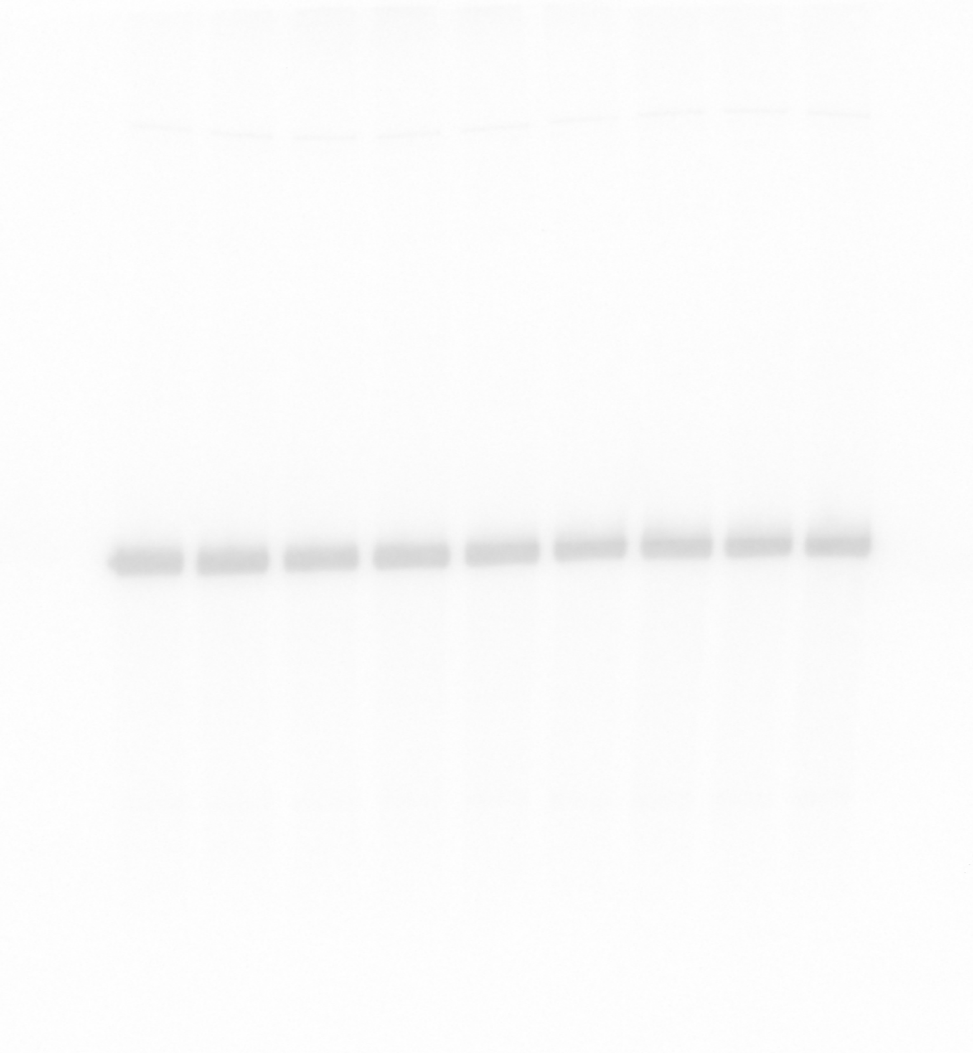


1 2 3 4 5 6 [lane]

OppZ (KPO-0845) 5S (KPO-0243)

# Figure 3 – figure supplement 1C

Data: *oppABCDF* transcript levels determined by qRT-PCR, fold change in pOppZ relative to pCtrl

|  | **fold rep 1** | **fold rep 2** | **fold rep 3** | **fold mean** | **SD** |
| --- | --- | --- | --- | --- | --- |
| *oppA* | 1.337 | 0.945 | 1.258 | 1.180 | 0.169 |
| *oppB* | 0.404 | 0.338 | 0.485 | 0.409 | 0.060 |
| *oppC* | 0.181 | 0.140 | 0.220 | 0.180 | 0.033 |
| *oppD* | 0.294 | 0.258 | 0.371 | 0.308 | 0.047 |
| *oppE* | 0.356 | 0.259 | 0.342 | 0.319 | 0.043 |

# Figure 3 – figure supplement 1D

Data: *oppB* transcript levels determined by qRT-PCR, fold change relative to t = 0 min

|  | **pCtrl** | | | | | **pOppZ** | | | | |
| --- | --- | --- | --- | --- | --- | --- | --- | --- | --- | --- |
| **[min]** | rep 1 | rep 2 | rep 3 | mean | SD | rep 1 | rep 2 | rep 3 | mean | SD |
| **0** | 1.000 | 1.000 | 1.000 | 1.000 | 0.000 | 1.000 | 1.000 | 1.000 | 1.000 | 0.000 |
| **1** | 0.844 | 0.808 | 0.739 | 0.797 | 0.069 | 0.505 | 0.640 | 0.483 | 0.543 | 0.069 |
| **2** | 0.234 | 0.221 | 0.186 | 0.214 | 0.004 | 0.069 | 0.070 | 0.061 | 0.067 | 0.004 |
| **3** | 0.059 | 0.065 | 0.064 | 0.063 | 0.004 | 0.032 | 0.035 | 0.042 | 0.036 | 0.004 |
| **4** | 0.033 | 0.040 | 0.042 | 0.038 | 0.002 | 0.023 | 0.023 | 0.027 | 0.024 | 0.002 |
