# Supplementary material for: Gene autoregulation by 3’ UTR-derived bacterial small RNAs
Source: eLife. 2020 Aug 3;9:e58836. doi: 10.7554/eLife.58836 (PMC7398697; doi:10.7554/eLife.58836)
Supplement: Figure 3—figure supplement 2—source data 1. [file elife-58836-fig3-figsupp2-data1.docx]

Source data for Figure 3 – figure supplement 2

Figure 3 – figure supplement 2A

Data: (fluorescence / OD600) - autofluorescence

| **sRNA** | **rep 1** | **rep 2** | **rep 3** |
| --- | --- | --- | --- |
| pCtrl | 250766.998 | 114006.625 | 201284.237 |
| pOppZ | 256789.78 | 129353.281 | 195539.517 |
| pOppZ M1 | 258744.26 | 129773.313 | 221616.852 |

Figure 3 – figure supplement 2B


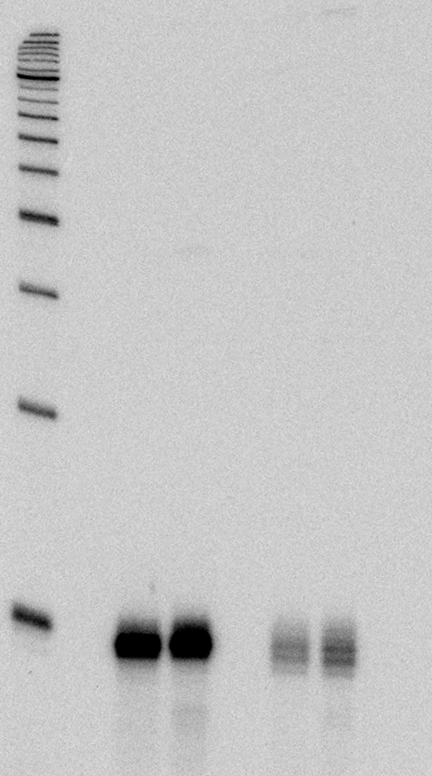


1 2 3 4 5 6 [lane]

OppZ (KPO-2687)


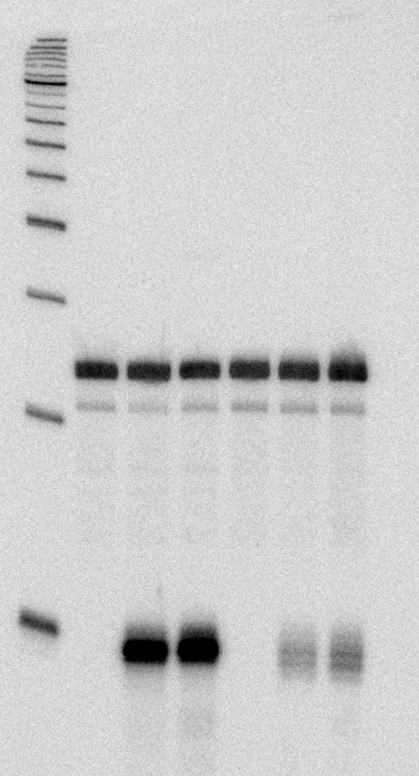


1 2 3 4 5 6 [lane]

5S (KPO-0243)

Figure 3 – figure supplement 2C

Data: (fluorescence / OD600) - autofluorescence

|  |  | **GFP** | | | **mKate** | | |
| --- | --- | --- | --- | --- | --- | --- | --- |
| **target** | sRNA | rep 1 | rep 2 | rep 3 | rep 1 | rep 2 | rep 3 |
| ***oppC*** | pCtrl | 146948 | 148987 | 145165 | 172030 | 166228 | 165915 |
|  | pOppZ | 32428 | 45376 | 29339 | 173024 | 170414 | 162363 |
|  | pOppZ M1 | 176547 | 177294 | 165847 | 214439 | 216317 | 212395 |
| ***oppD*** | pCtrl | 86261 | 85659 | 86573 | 92581 | 80363 | 82164 |
|  | pOppZ | 14218 | 14718 | 14068 | 94891 | 86677 | 83236 |
|  | pOppZ M1 | 90320 | 92716 | 94514 | 104463 | 94313 | 93750 |
| ***oppF*** | pCtrl | 124443 | 128568 | 130940 | 85665 | 79828 | 72171 |
|  | pOppZ | 25176 | 26169 | 24263 | 87551 | 86063 | 95053 |
|  | pOppZ M1 | 154775 | 162380 | 159499 | 103658 | 102131 | 100759 |
| ***oppF* M1** | pCtrl | 131629 | 130497 | 133717 | 84622 | 86158 | 84789 |
|  | pOppZ | 137830 | 142594 | 140001 | 106032 | 105197 | 107753 |
|  | pOppZ M1 | 35421 | 35743 | 34979 | 97128 | 96908 | 99259 |
