# Supplementary material for: Gene autoregulation by 3’ UTR-derived bacterial small RNAs
Source: eLife. 2020 Aug 3;9:e58836. doi: 10.7554/eLife.58836 (PMC7398697; doi:10.7554/eLife.58836)
Supplement: Figure 4—source data 1. [file elife-58836-fig4-data1.docx]

# Figure 4B

kDa


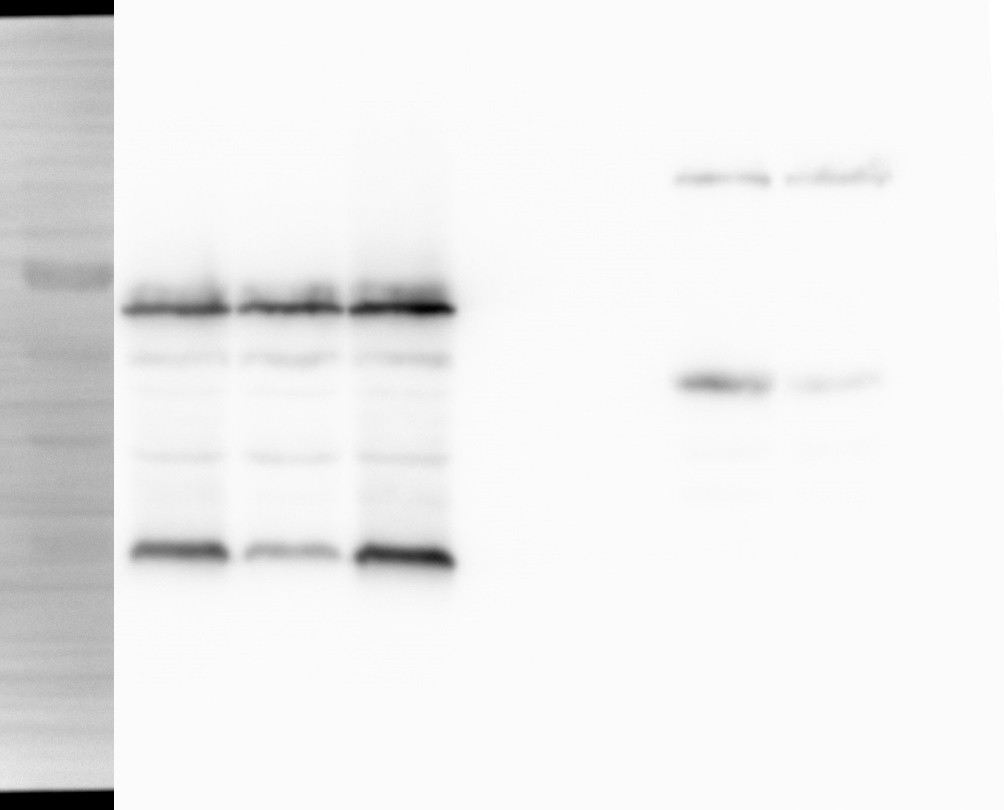


1 2 3 [lane]

α-FLAG

| 70 |  | OppA | 70 |
| --- | --- | --- | --- |
| 55 |  |  | 55 |
| 40 |  |  | 40 |
| 35 |  | OppB | 35 |


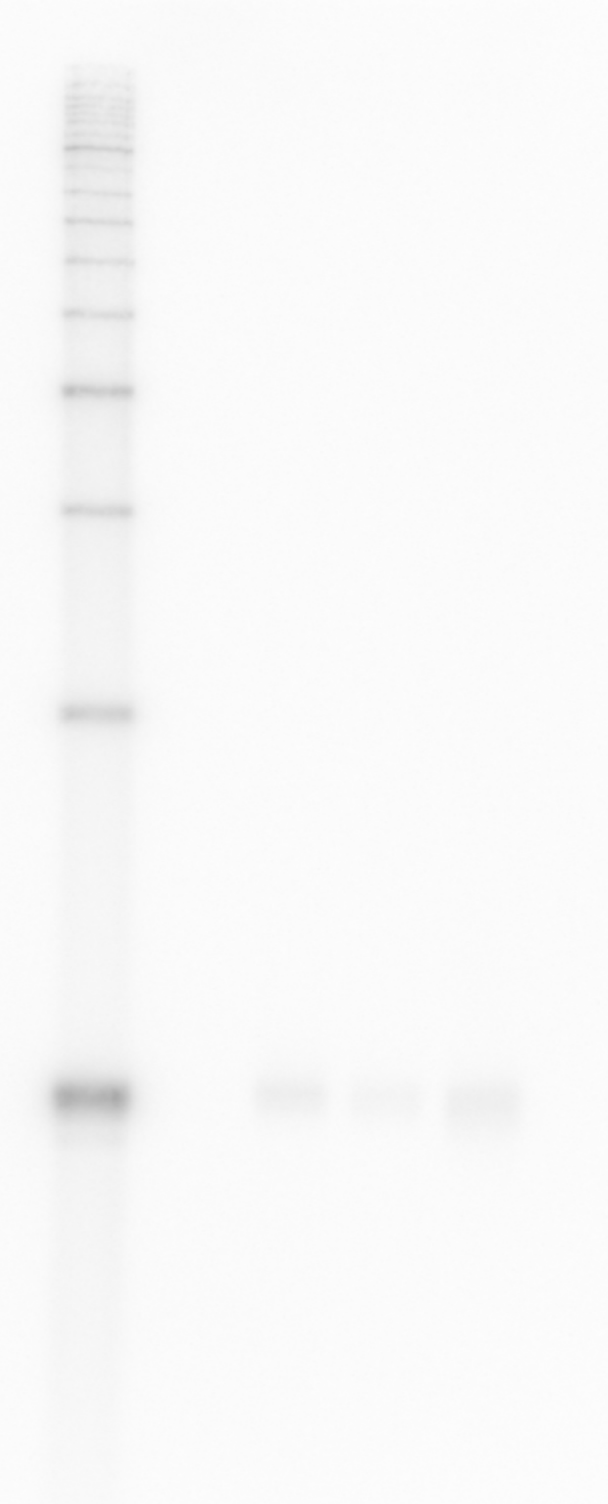


1 2 3 [lane]


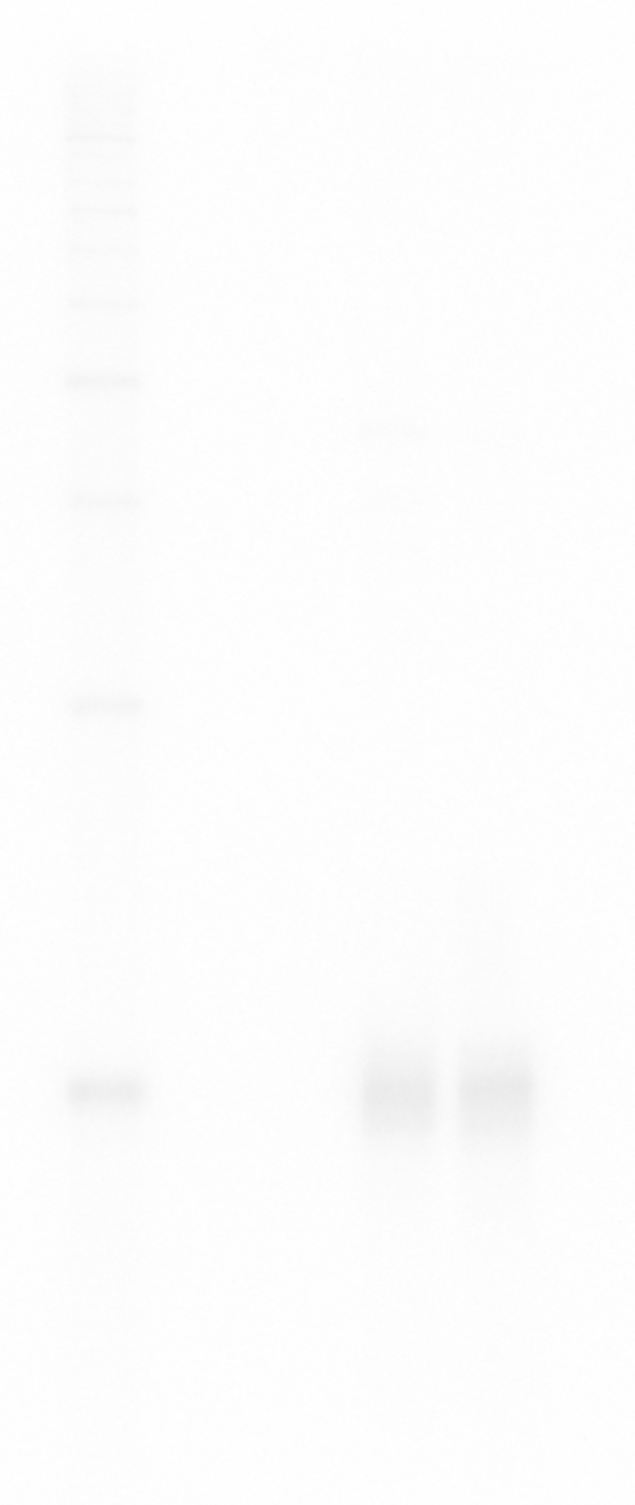


1 2 3 [lane]


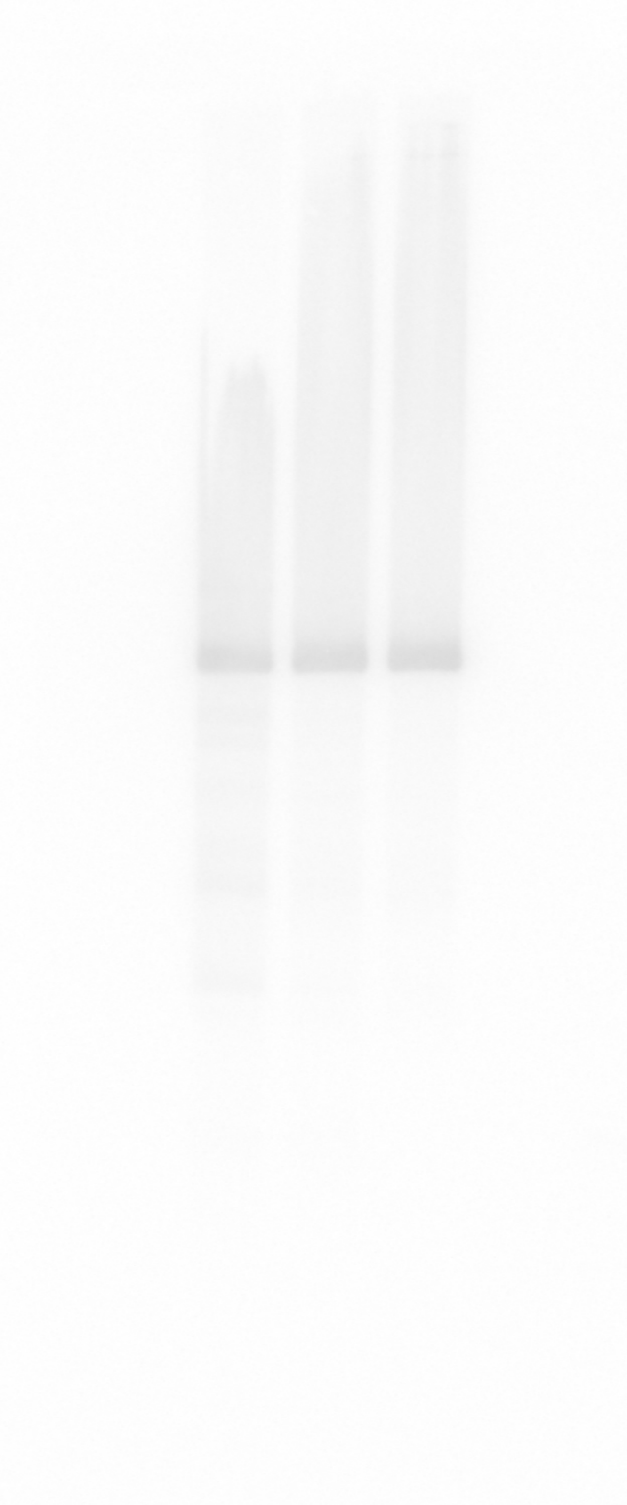


1 2 3 [lane]

native OppZ (KPO-2688)


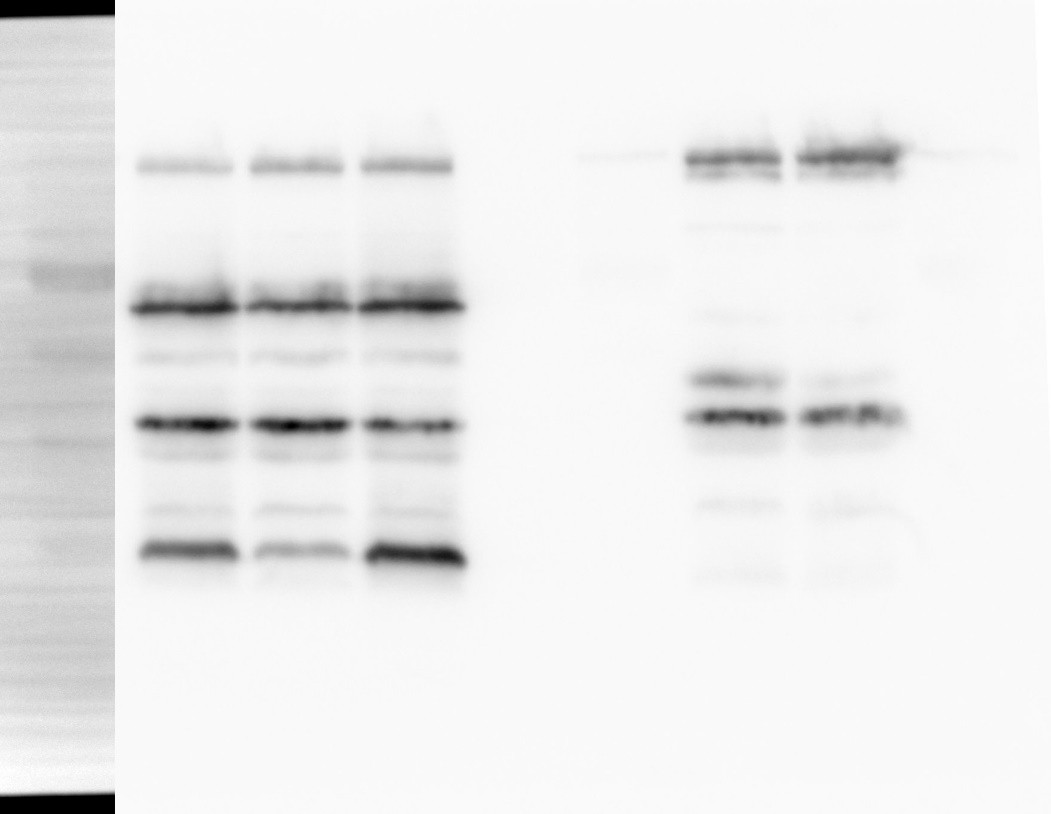


1 2 3 [lane]

kDa

RNAP

α-RNAP

regulator OppZ (KPO-3192)

5S (KPO-0243)


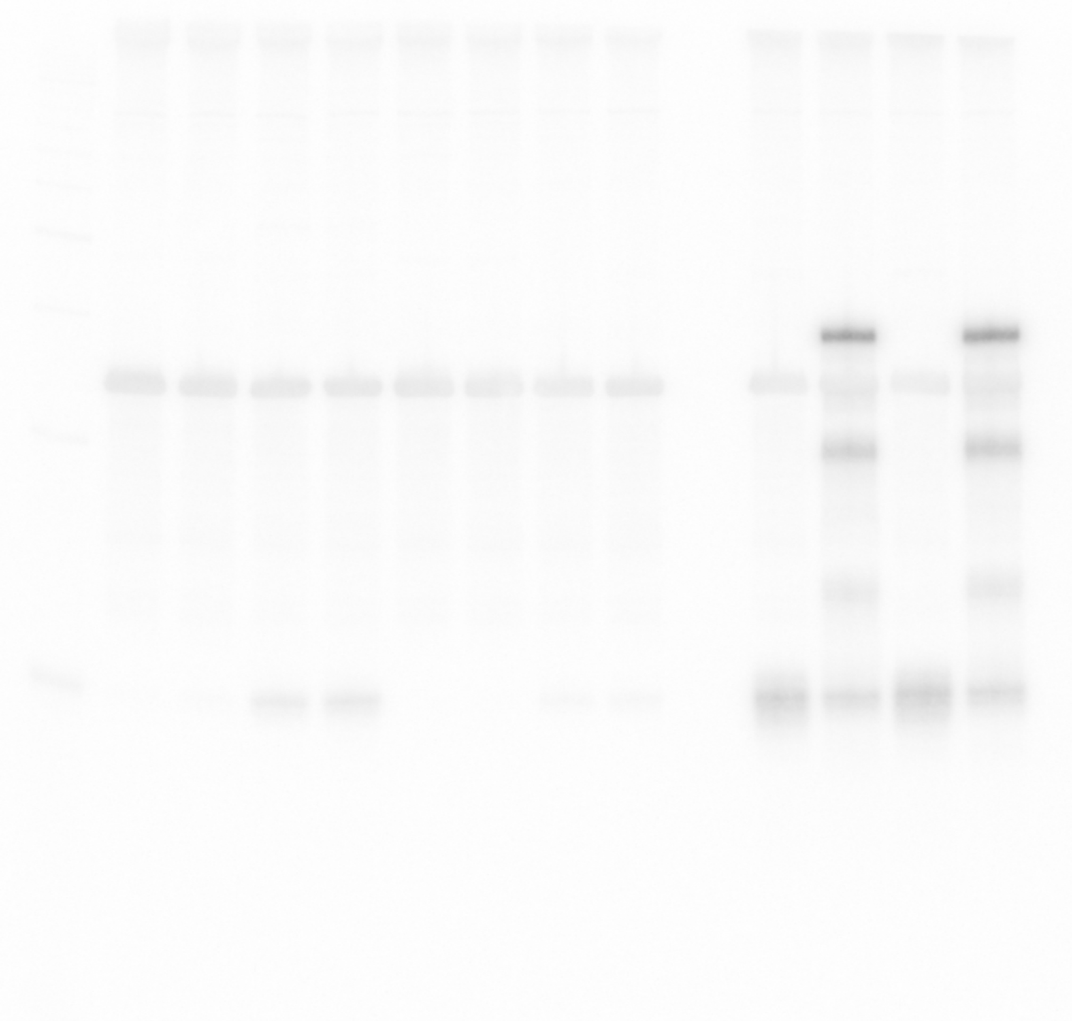


1 2 3 4 5 6 7 8 [lane]


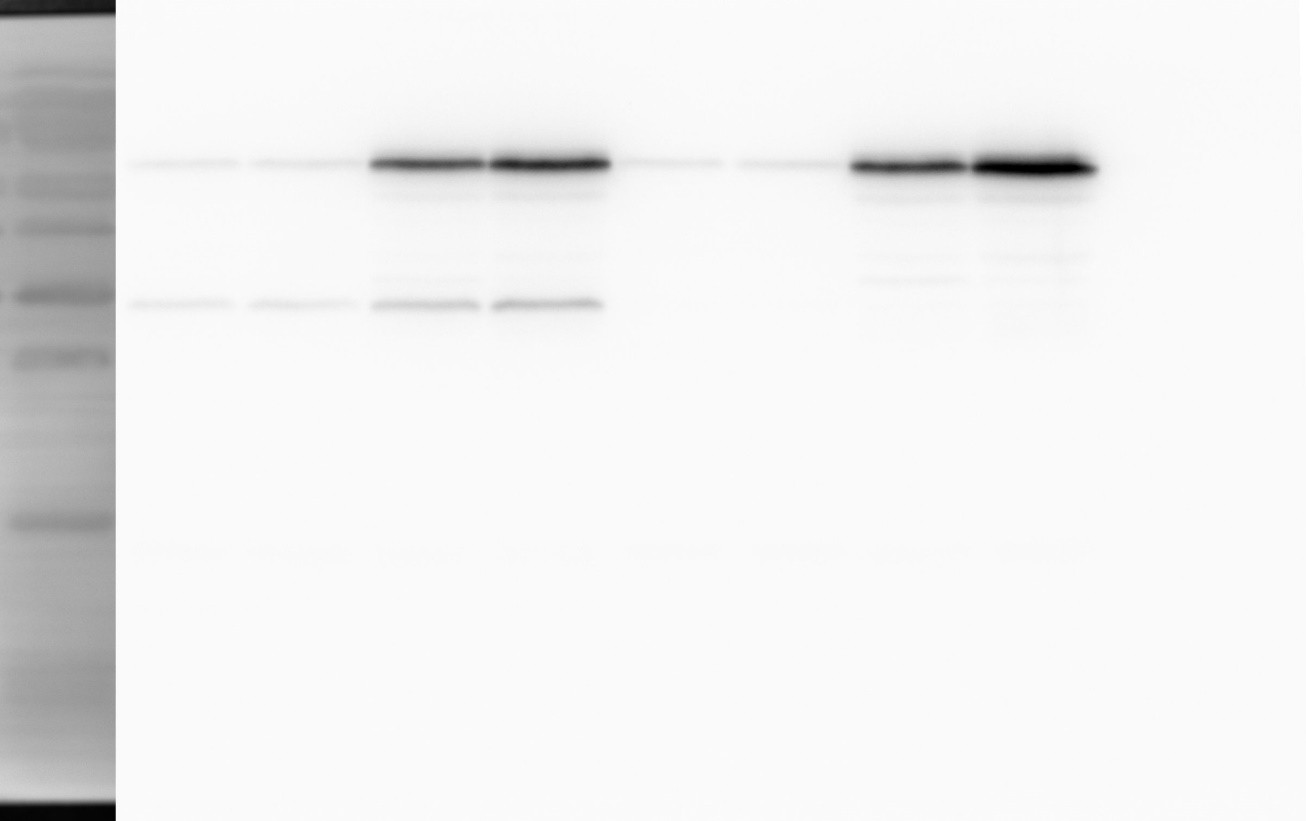


kDa 70

55

40

35

25

1

2

3

4 5

6 7

8 [lane]

OppA

OppB

α-FLAG


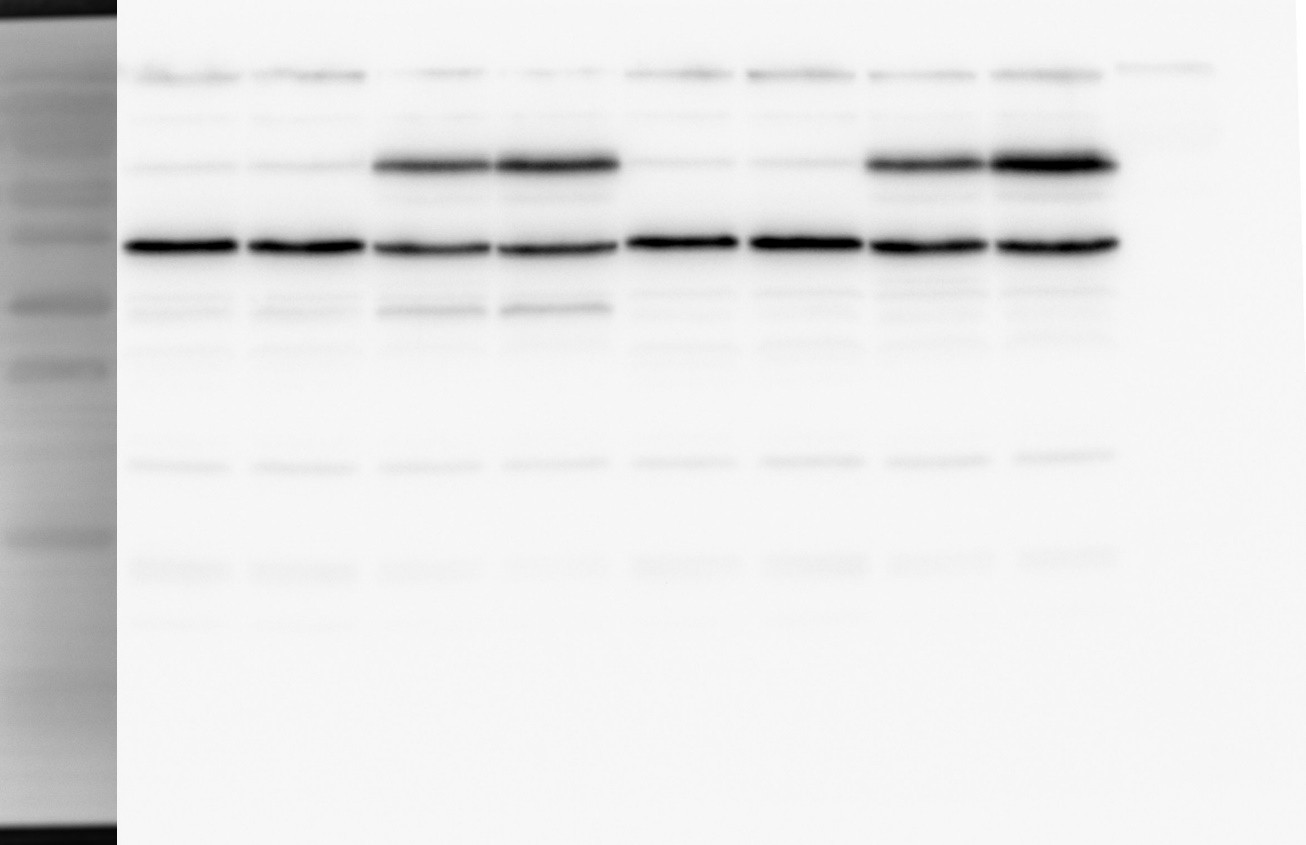


kDa 70

55

40

RNAP

35

25

1

2

3

4 5

6 7

8 [lane]

α-RNAP


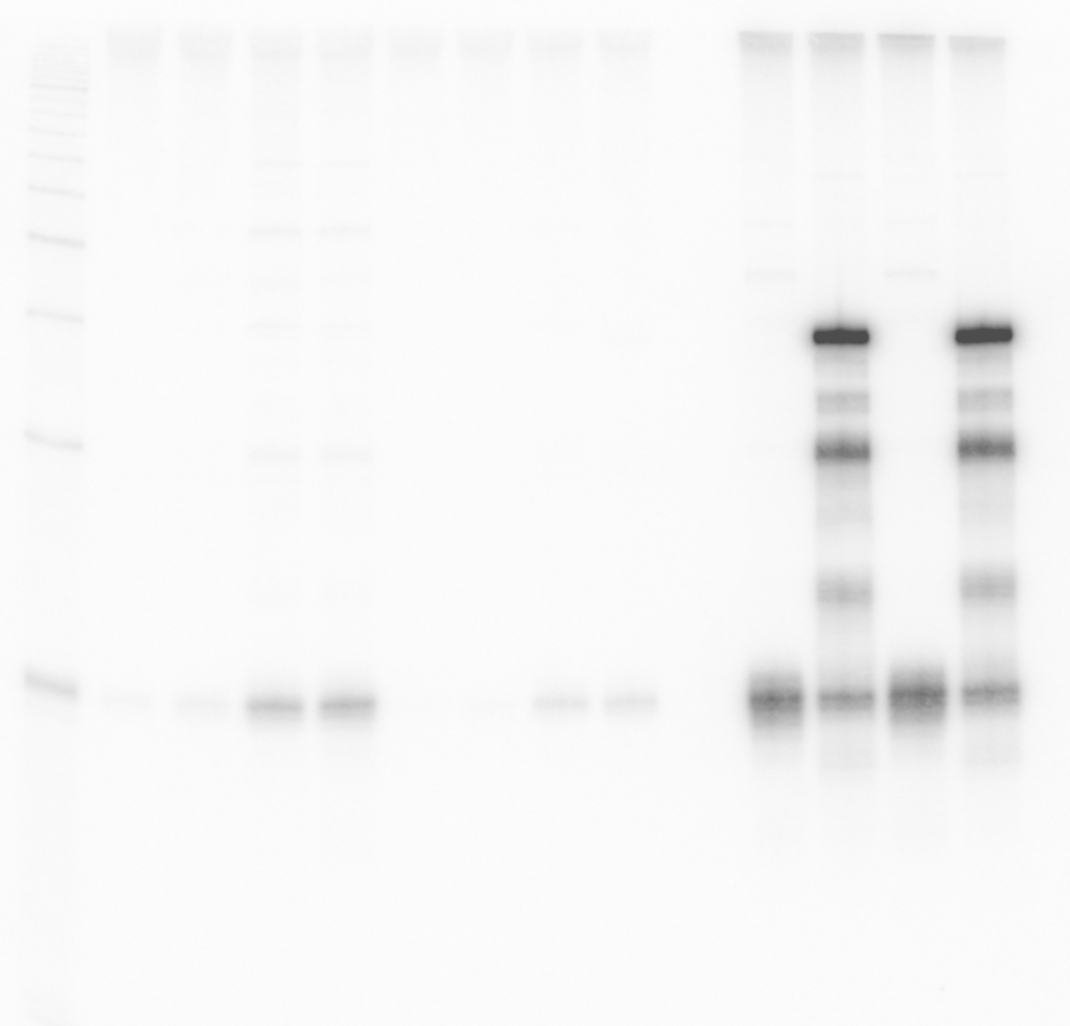


1 2 3 4 5 6 7 8 [lane]

OppZ (KPO-0845) 5S (KPO-0243)
