# Supplementary material for: Gene autoregulation by 3’ UTR-derived bacterial small RNAs
Source: eLife. 2020 Aug 3;9:e58836. doi: 10.7554/eLife.58836 (PMC7398697; doi:10.7554/eLife.58836)
Supplement: Figure 4—figure supplement 1—source data 1. [file elife-58836-fig4-figsupp1-data1.docx]

# Figure 4 – figure supplement 1A

Data:

- OppZ and 5S quantified from three biological replicates on Northern blots
- OppZ normalized to 5S levels
- Fold change relative to t = 0 min

| **strain** | **min** | **rep 1** | **rep 2** | **rep 3** | **mean** | **SD** |
| --- | --- | --- | --- | --- | --- | --- |
| **WT** | 0 | 1.0000 | 1.0000 | 1.0000 | 1.0000 | 0.0000 |
|  | 2 | 1.1272 | 1.8005 | 1.1009 | 1.3429 | 0.3238 |
|  | 4 | 1.1031 | 1.1159 | 1.1230 | 1.1140 | 0.0083 |
|  | 8 | 0.8733 | 0.9310 | 1.2083 | 1.0042 | 0.1462 |
|  | 16 | 1.3418 | 1.0955 | 1.1696 | 1.2023 | 0.1032 |
|  | 32 | 1.0512 | 0.9424 | 1.1430 | 1.0455 | 0.0820 |
| ***oppB* ATC** | 0 | 1.0000 | 1.0000 | 1.0000 | 1.0000 | 0.0000 |
|  | 2 | 1.5245 | 1.0153 | 0.9619 | 1.1673 | 0.2536 |
|  | 4 | 1.7158 | 0.9489 | 1.1030 | 1.2559 | 0.3312 |
|  | 8 | 1.4386 | 1.1048 | 1.2175 | 1.2536 | 0.1386 |
|  | 16 | 1.2780 | 0.9040 | 1.0009 | 1.0609 | 0.1585 |
|  | 32 | 0.8014 | 0.7710 | 0.9999 | 0.8574 | 0.1015 |

kDa


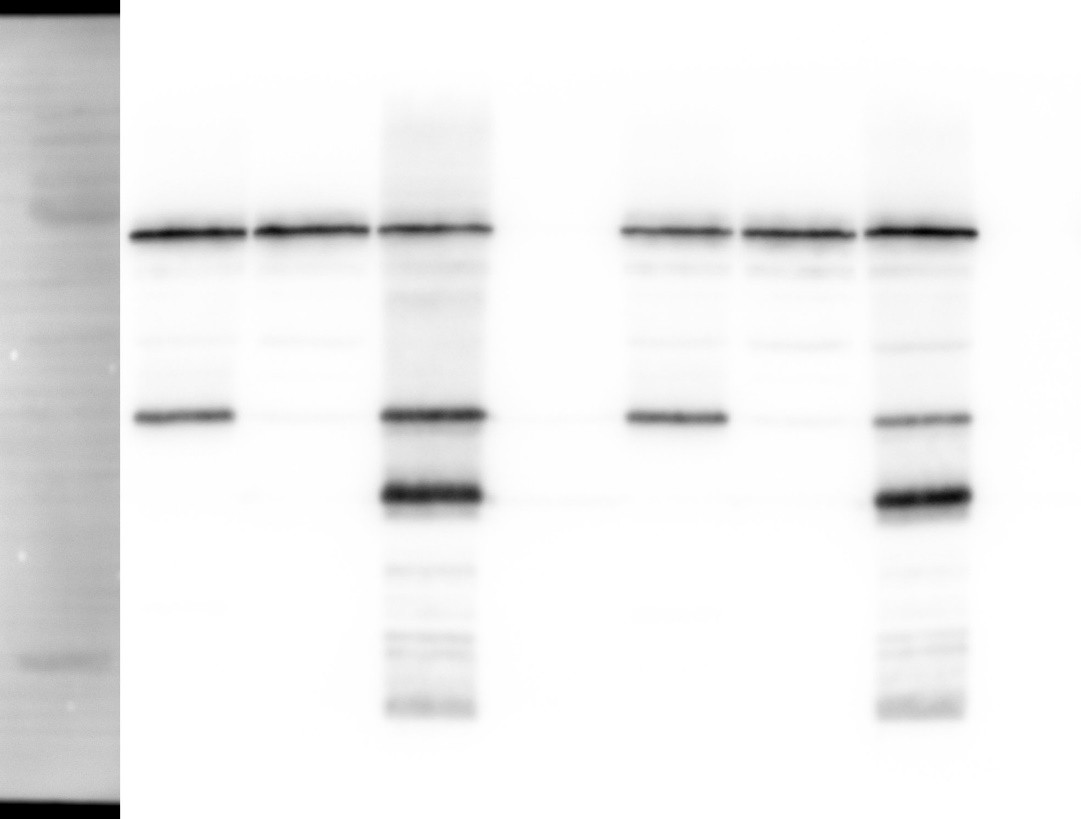


1

2

3 [lane]

α-FLAG

kDa


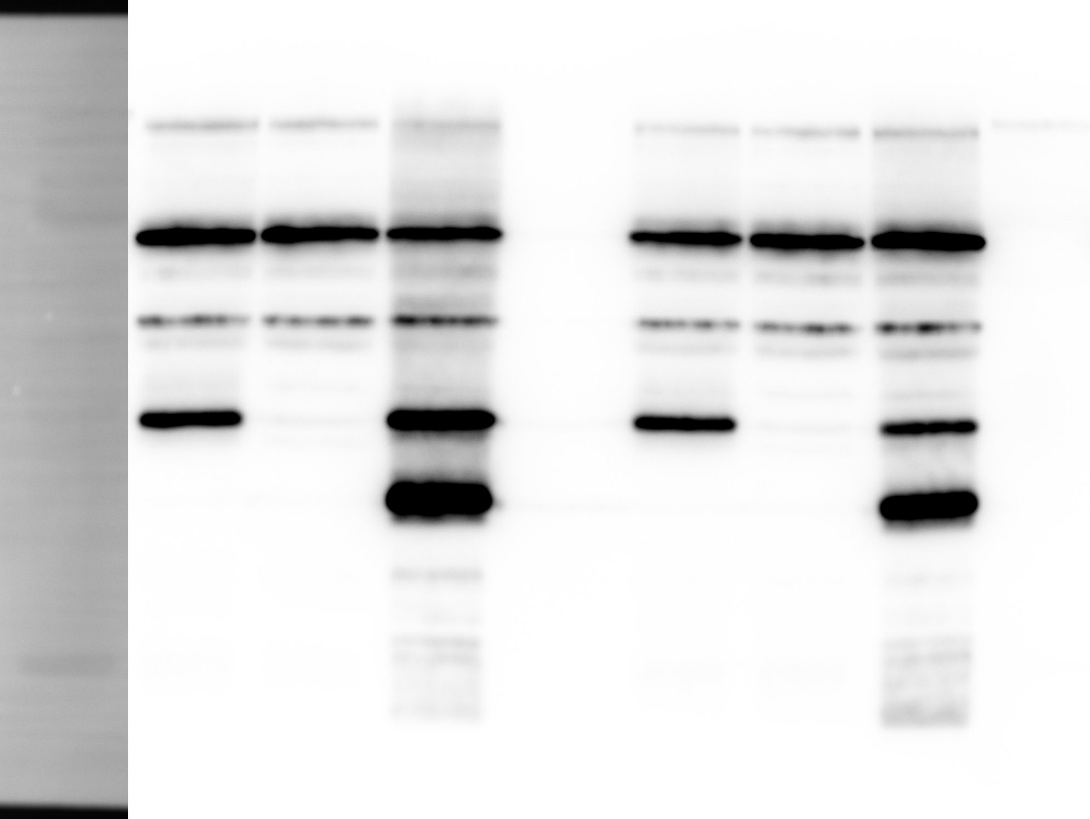


1

2

3 [lane]

RNAP

α-RNAP

| 70  55 |  | OppA | 70  55 |
| --- | --- | --- | --- |
| 40 |  |  | 40 |
| 35 |  | OppB | 35 |
| 25 |  |  | 25 |


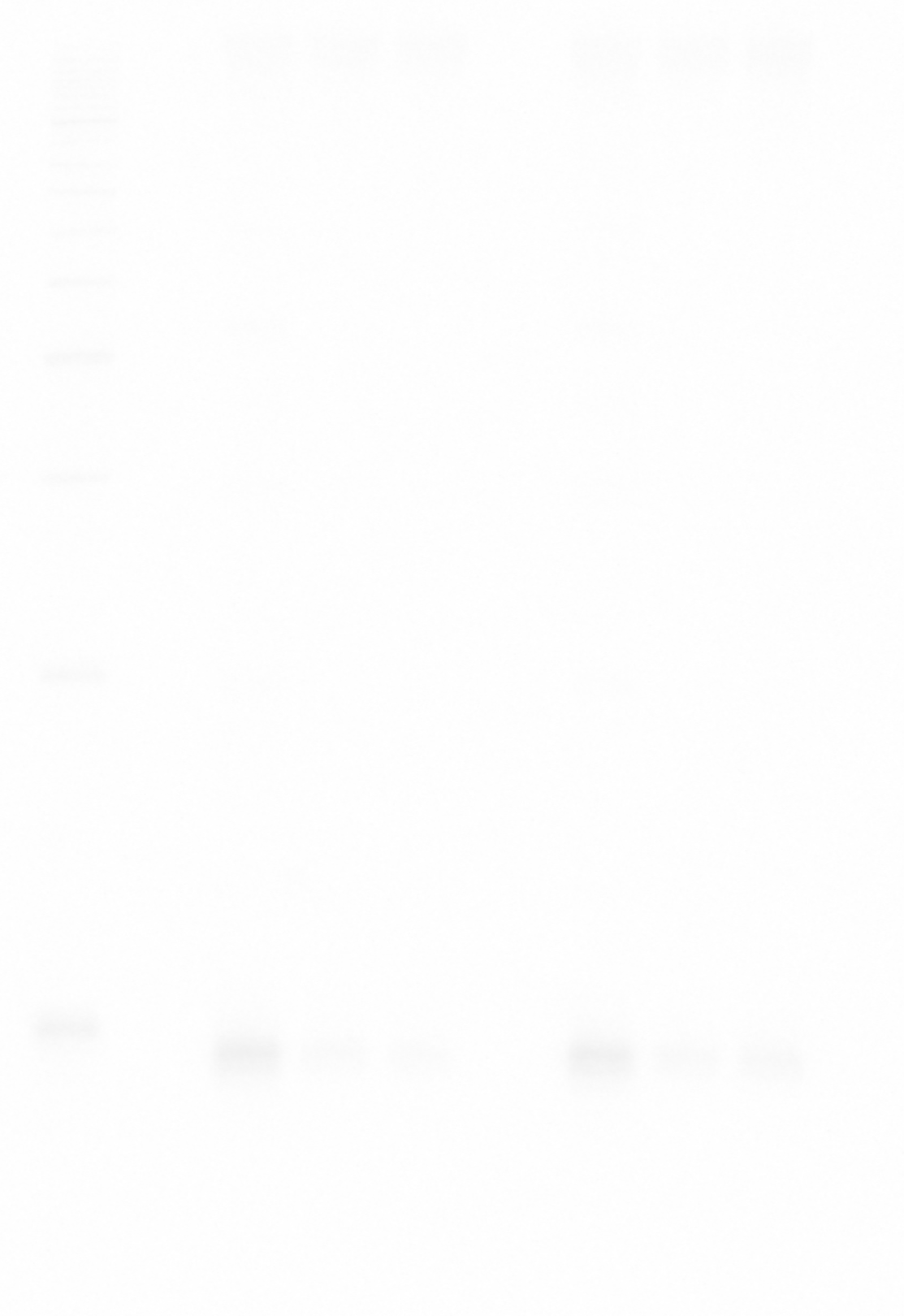


1 2 3 [lane]


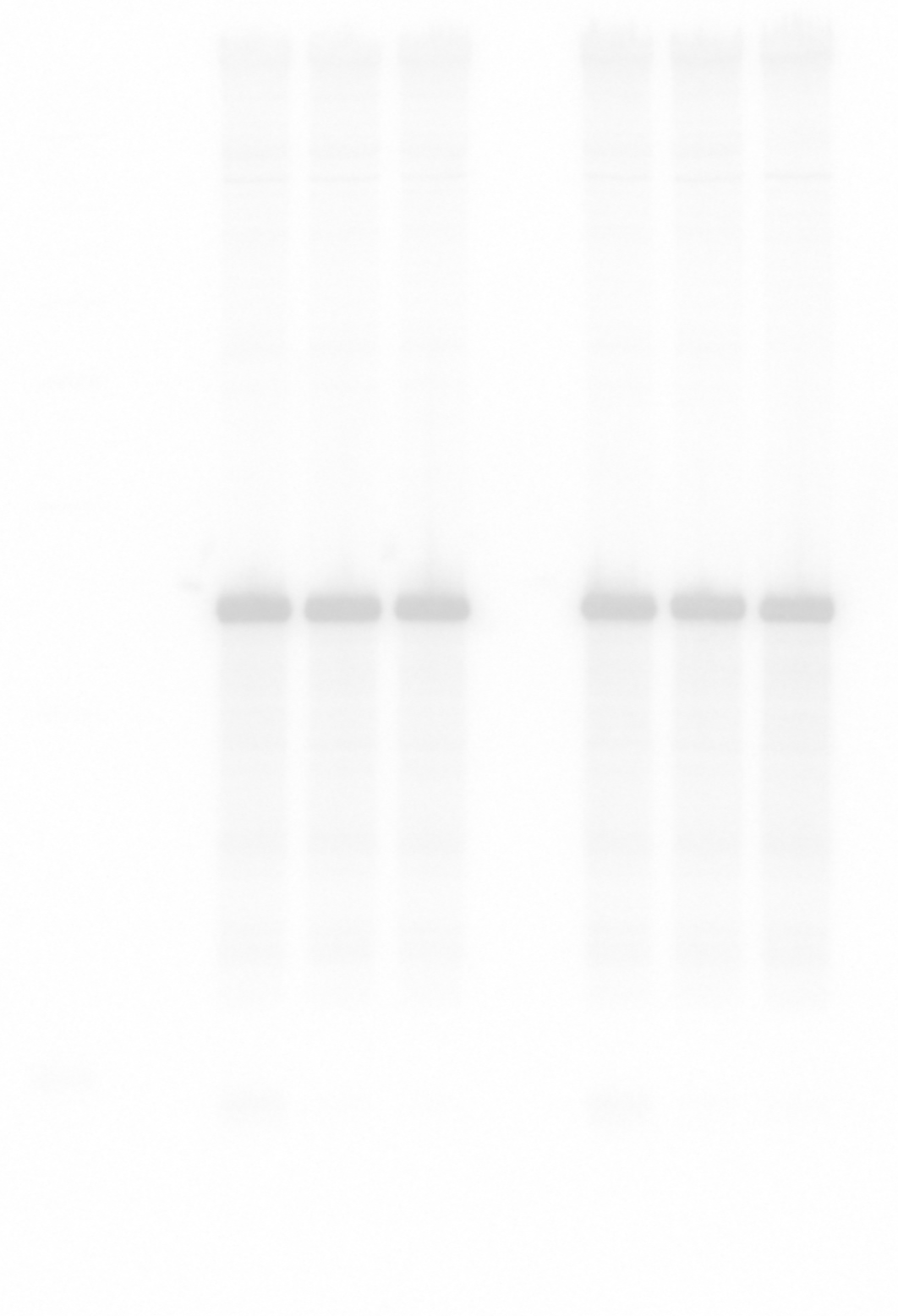


1 2 3 [lane]

OppZ (KPO-0845) 5S (KPO-0243)
