# Supplementary material for: Gene autoregulation by 3’ UTR-derived bacterial small RNAs
Source: eLife. 2020 Aug 3;9:e58836. doi: 10.7554/eLife.58836 (PMC7398697; doi:10.7554/eLife.58836)
Supplement: Figure 5—source data 1. [file elife-58836-fig5-data1.docx]

# Figure 5A

kDa 70

55

40

35

25

1 2 3 4 [lane]

OppA


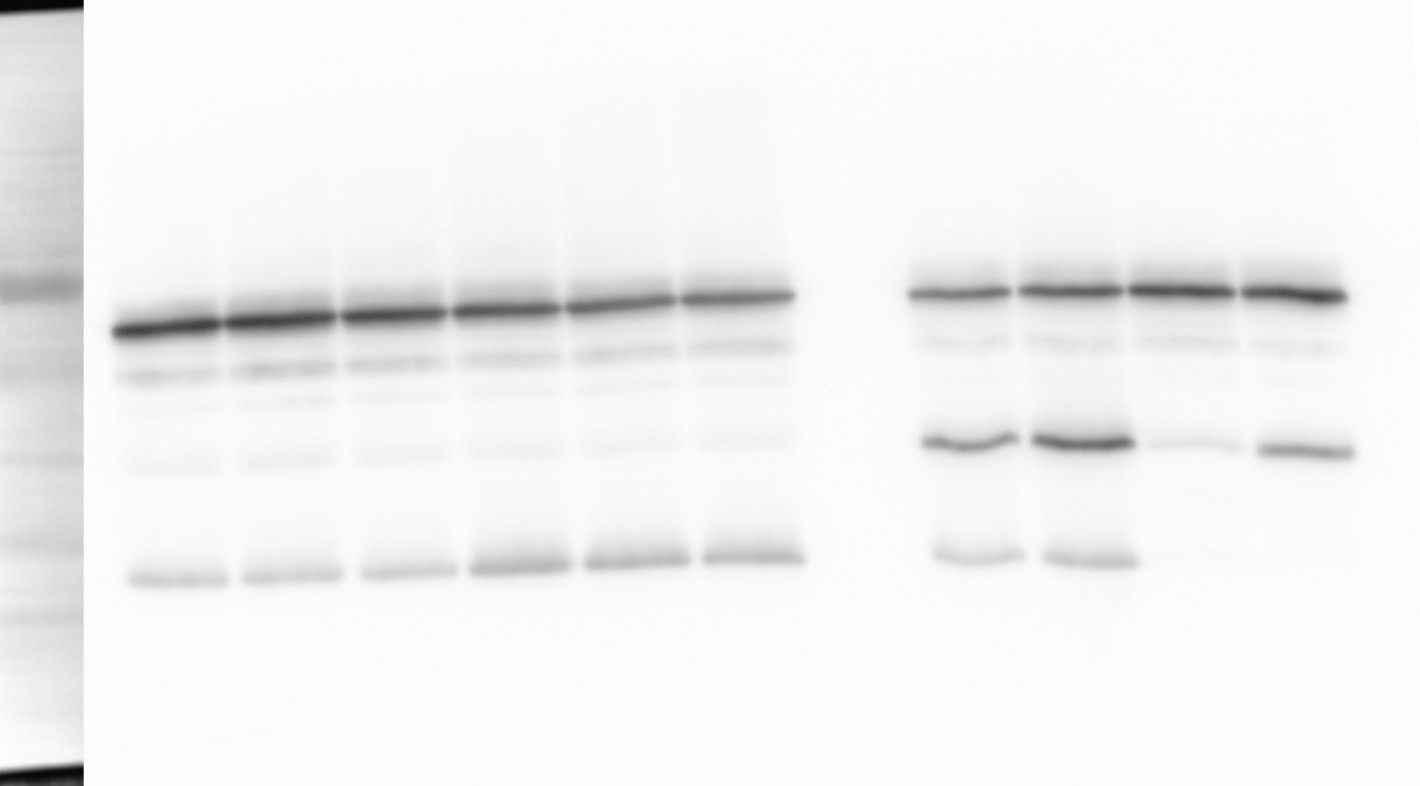


OppF OppB

α-FLAG

kDa 70


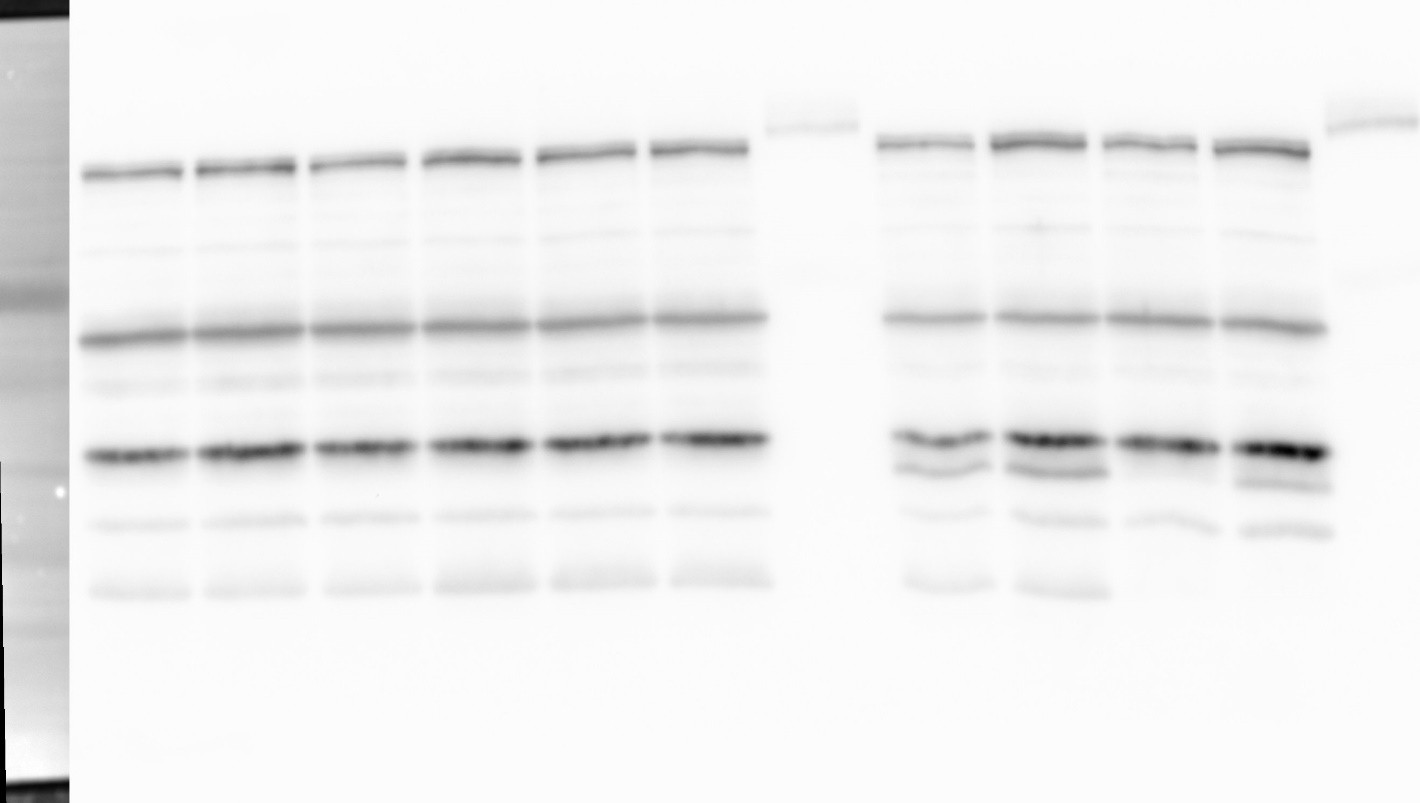


1 2 3 4 [lane]

RNAP

α-RNAP

55

40

35

25

1 2 3 4 [lane]


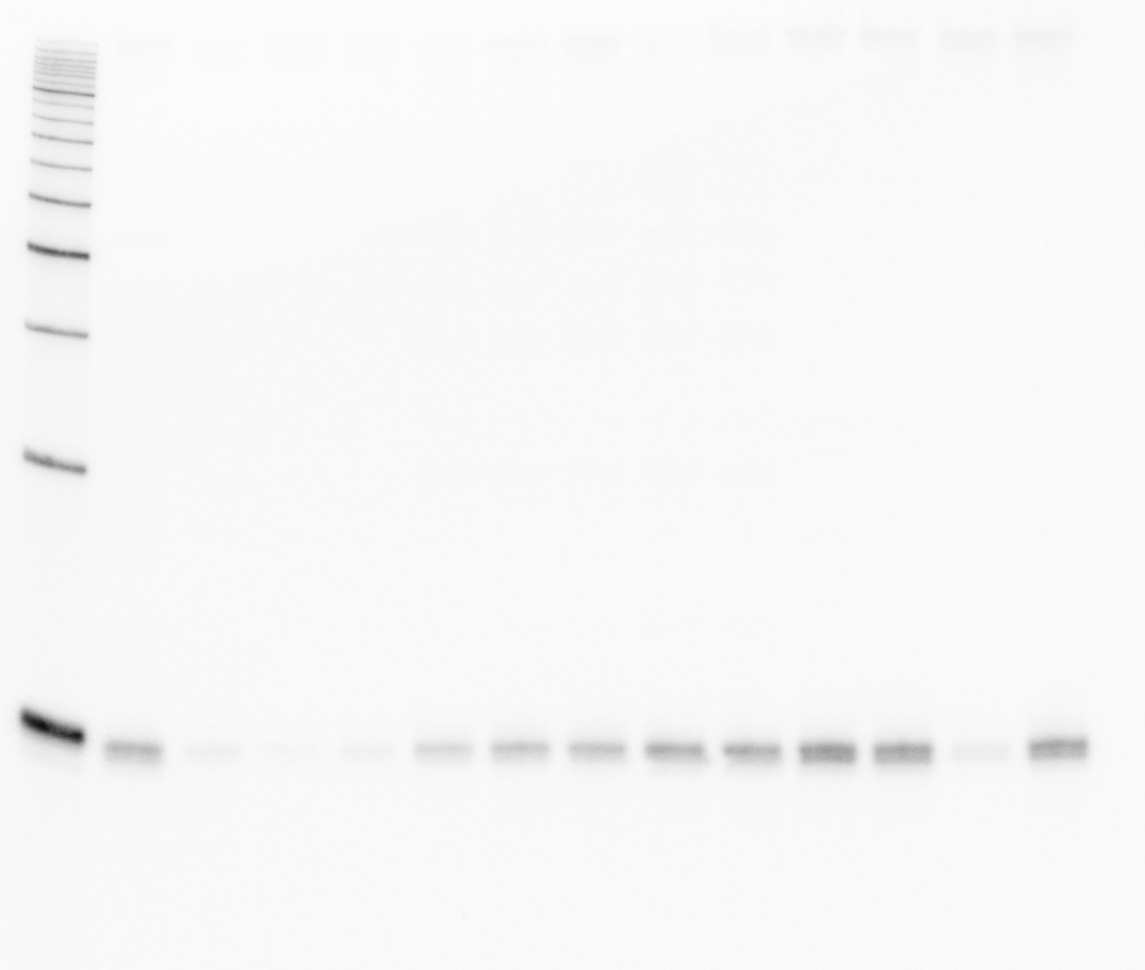


1 2 3 4 [lane]


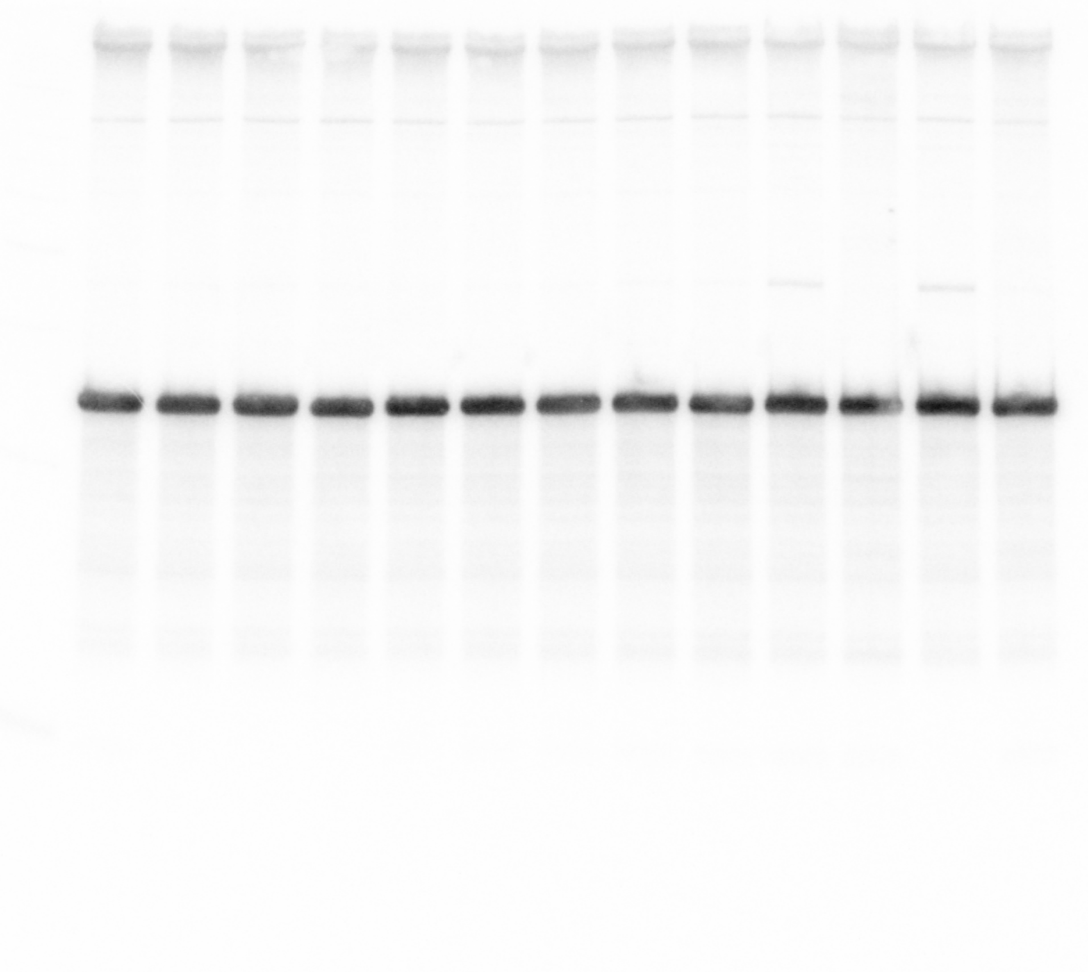


OppZ (KPO-0845) 5S (KPO-0243)

# Figure 5B

Data: transcript levels determined by qRT-PCR, fold change in oppB ATC relative to wild-type

| **mock treatment** | | | | | |
| --- | --- | --- | --- | --- | --- |
|  | fold rep 1 | fold rep 2 | fold rep 3 | fold mean | SD |
| ***oppA*** | 0.78645819 | 0.95532929 | 0.81542486 | 0.85240412 | 0.07373358 |
| ***oppB*** | 0.32898495 | 0.35991672 | 0.51671573 | 0.40187246 | 0.08218242 |
| ***oppC*** | 0.06759312 | 0.07983918 | 0.10012996 | 0.08252075 | 0.01341777 |
| ***oppD*** | 0.06501343 | 0.08597138 | 0.09670638 | 0.08256373 | 0.01316105 |
| ***oppF*** | 0.06159395 | 0.07443344 | 0.09041392 | 0.07548044 | 0.01178897 |
|  |  |  |  |  |  |
| **bicyclomycin treatment** | | | | | |
|  | fold rep 1 | fold rep 2 | fold rep 3 | fold mean | SD |
| ***oppA*** | 1.23050985 | 0.92862701 | 0.83182355 | 0.99698681 | 0.16978906 |
| ***oppB*** | 0.83290544 | 0.71407962 | 1.23121388 | 0.92606631 | 0.22115782 |
| ***oppC*** | 0.5353467 | 0.60844859 | 0.63623383 | 0.59334304 | 0.04254947 |
| ***oppD*** | 0.69922294 | 0.68575921 | 0.71795186 | 0.700978 | 0.01320106 |
| ***oppF*** | 0.68410193 | 0.63075942 | 0.63699785 | 0.65061973 | 0.02381208 |

**Figure 5D**

kDa 70


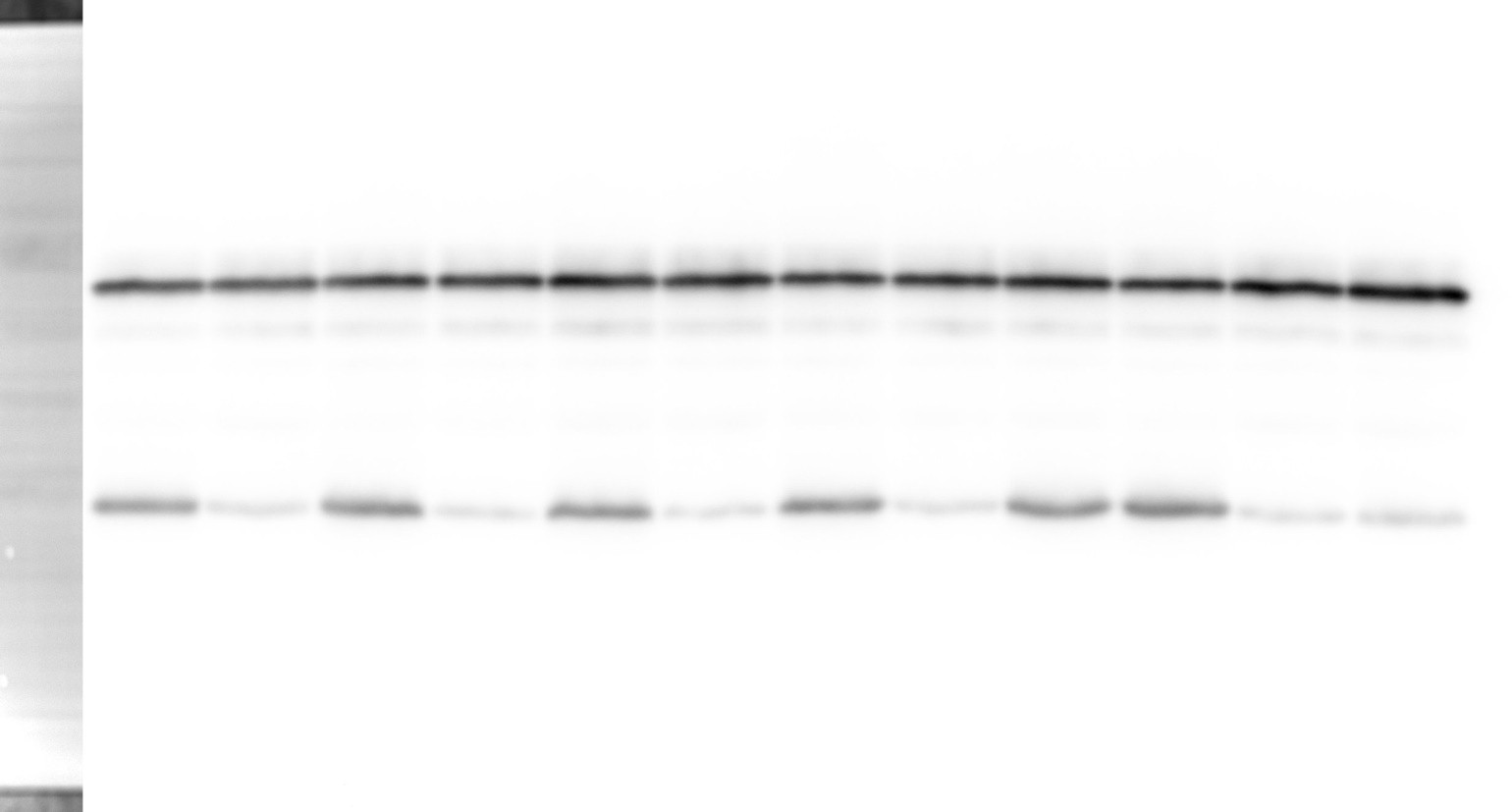


55

40

35

25

1 2 3 4 [lane]

OppA

OppB

α-FLAG

kDa 70

55

40

35

25

1 2 3 4 [lane]

RNAP


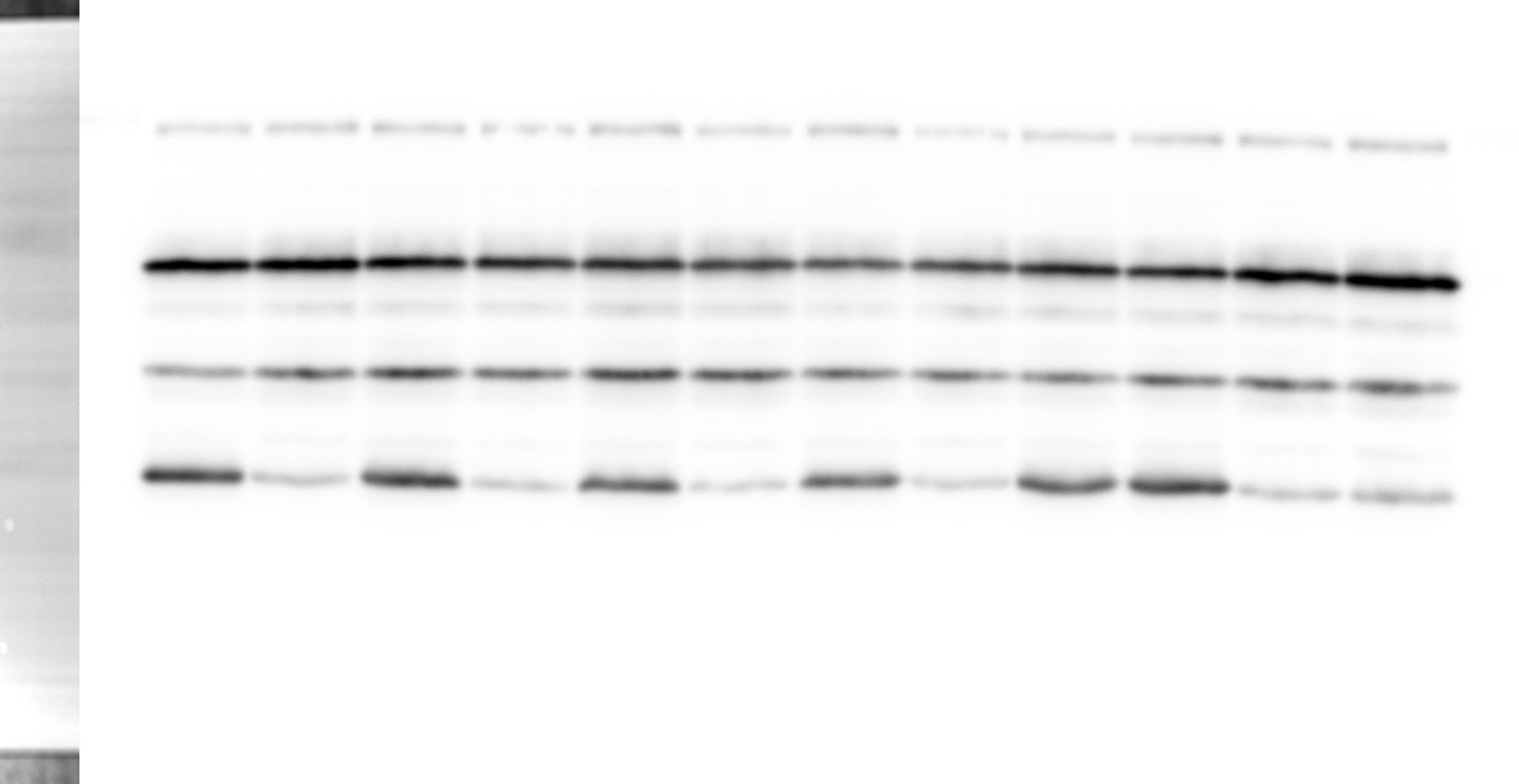


α-RNAP

1 2 3 4 [lane]


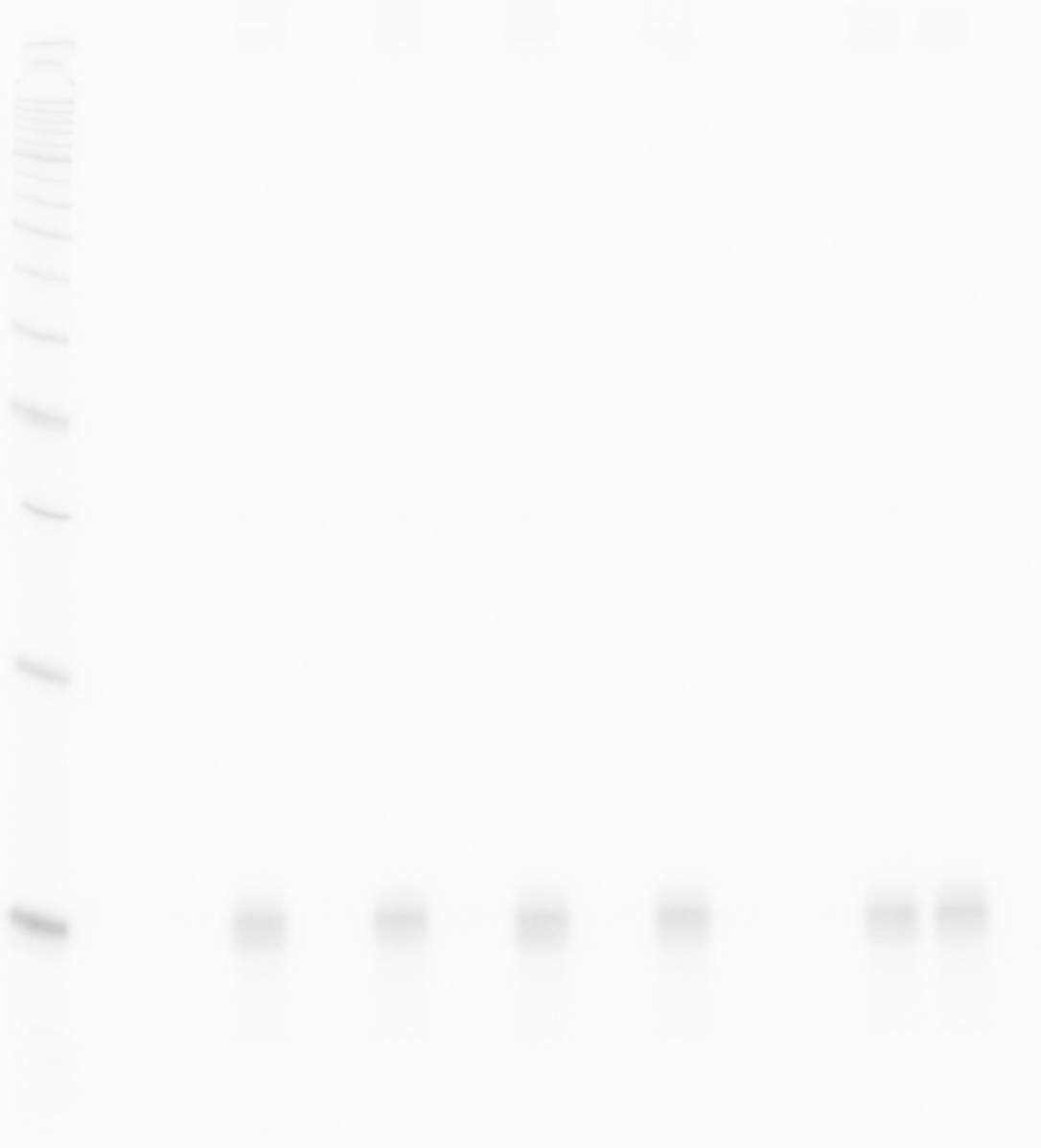

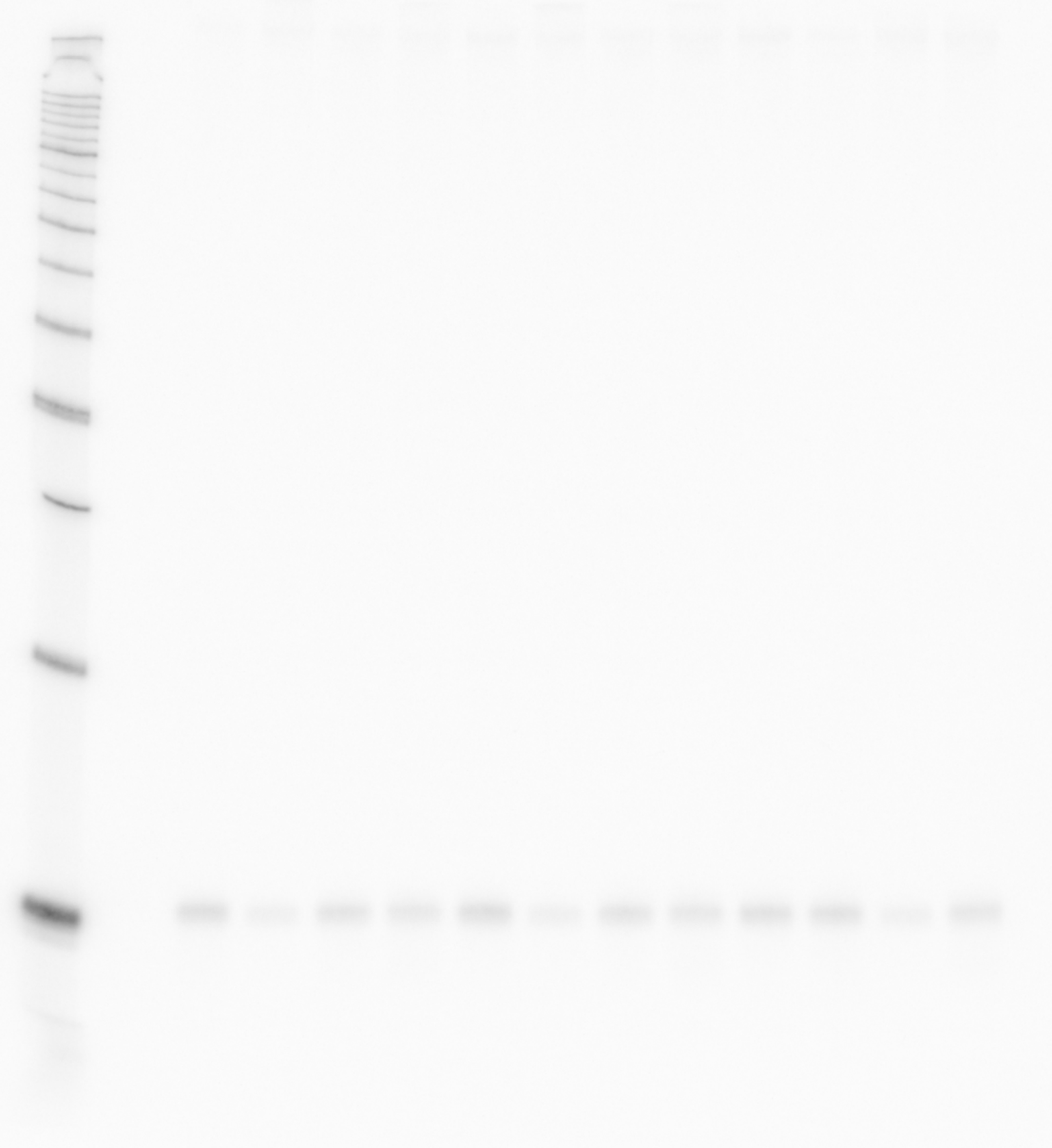


1 2 3 4 [lane]

Native OppZ (KPO-2688)

Regulator OppZ (KPO-3192)


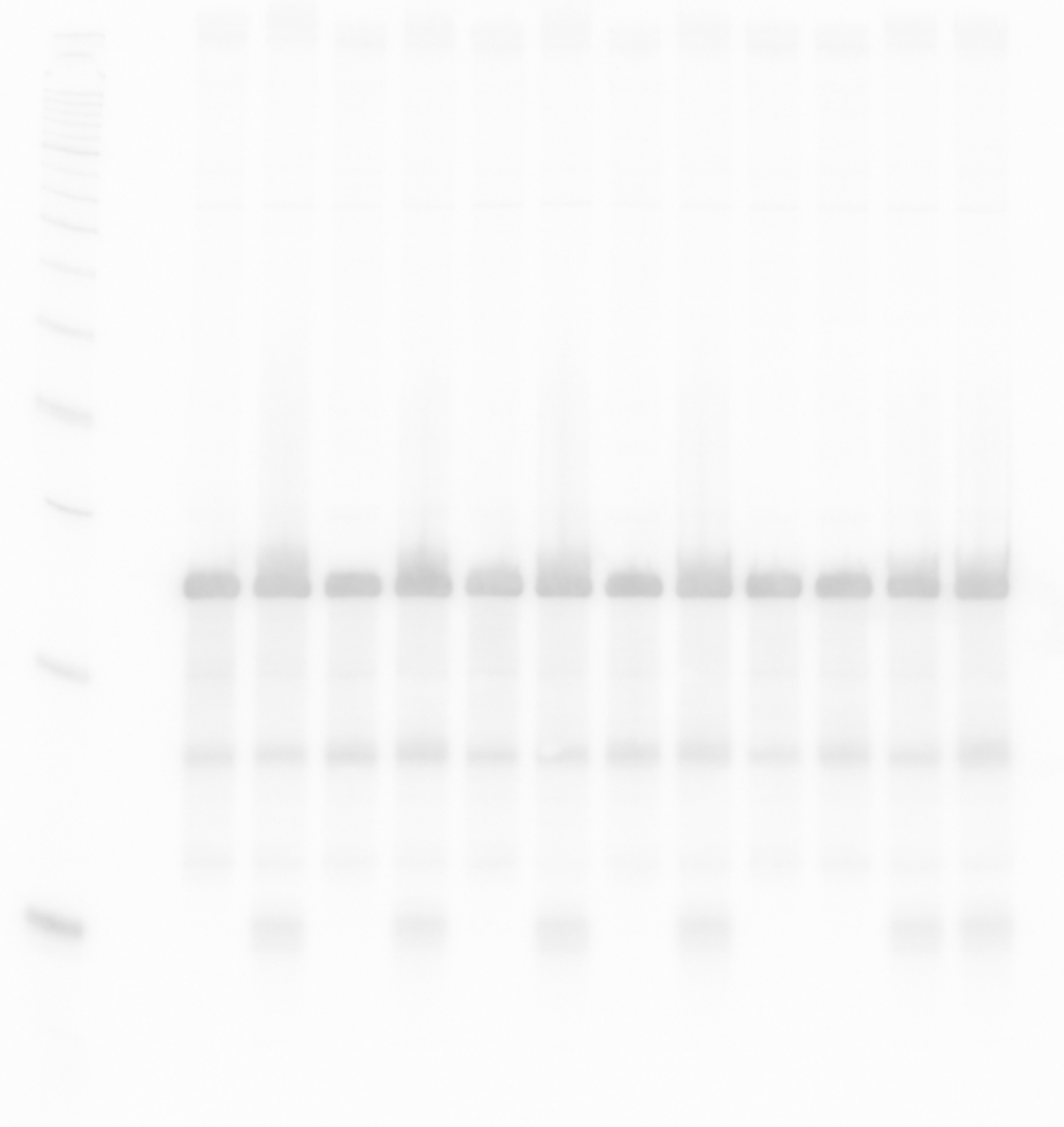


1 2 3 4 [lane]

5S (KPO-0243)

# Figure 5E

Data: transcript levels determined by qRT-PCR, fold change in pOppZ relative to pCtrl

| **mock treatment** | | | | | |
| --- | --- | --- | --- | --- | --- |
|  | fold rep 1 | fold rep 2 | fold rep 3 | fold mean | SD |
| ***oppA*** | 1.39119129 | 0.79230037 | 0.76172727 | 0.98173964 | 0.28979495 |
| ***oppB*** | 0.53299772 | 0.47831301 | 0.37873358 | 0.4633481 | 0.06386088 |
| ***oppC*** | 0.35846617 | 0.25841789 | 0.24660979 | 0.28783129 | 0.0501785 |
| ***oppD*** | 0.44370677 | 0.39793552 | 0.32672651 | 0.38945627 | 0.04813189 |
| ***oppF*** | 0.29283168 | 0.39318922 | 0.23718419 | 0.30773503 | 0.06455475 |
|  |  |  |  |  |  |
| **bicyclomycin treatment** | | | | | |
|  | fold rep 1 | fold rep 2 | fold rep 3 | fold mean | SD |
| ***oppA*** | 1.5135985 | 1.15847985 | 1.13398126 | 1.26868654 | 0.17346748 |
| ***oppB*** | 0.84152379 | 0.76807016 | 1.05561411 | 0.88840268 | 0.12197979 |
| ***oppC*** | 0.85240972 | 0.58331607 | 0.54697454 | 0.66090011 | 0.13622806 |
| ***oppD*** | 0.79712055 | 0.84670324 | 0.68970124 | 0.77784168 | 0.06552945 |
| ***oppF*** | 0.50442611 | 0.92550597 | 0.60345454 | 0.67779554 | 0.17976281 |
