# Supplementary material for: Gene autoregulation by 3’ UTR-derived bacterial small RNAs
Source: eLife. 2020 Aug 3;9:e58836. doi: 10.7554/eLife.58836 (PMC7398697; doi:10.7554/eLife.58836)
Supplement: Figure 7—source data 1. [file elife-58836-fig7-data1.docx]

# Figure 7C

Data: (fluorescence / OD600) - autofluorescence

| **5‘ end** | **3‘ UTR** | **rep 1** | **rep 2** | **rep 3** |
| --- | --- | --- | --- | --- |
| ***carA*** | no *carZ* | 143552.092 | 140492.98 | 143981.507 |
|  | *carZ* | 11372.015 | 10917.6514 | 11351.3335 |
|  | *carZ* M1 | 246591.982 | 262134.049 | 249504.585 |
| ***carA* M1** | no *carZ* | 158593.635 | 157321.165 | 155981.291 |
|  | *carZ* | 268753.441 | 274478.496 | 274550.428 |
|  | *carZ* M1 | 13655.605 | 12810.717 | 12925.8406 |


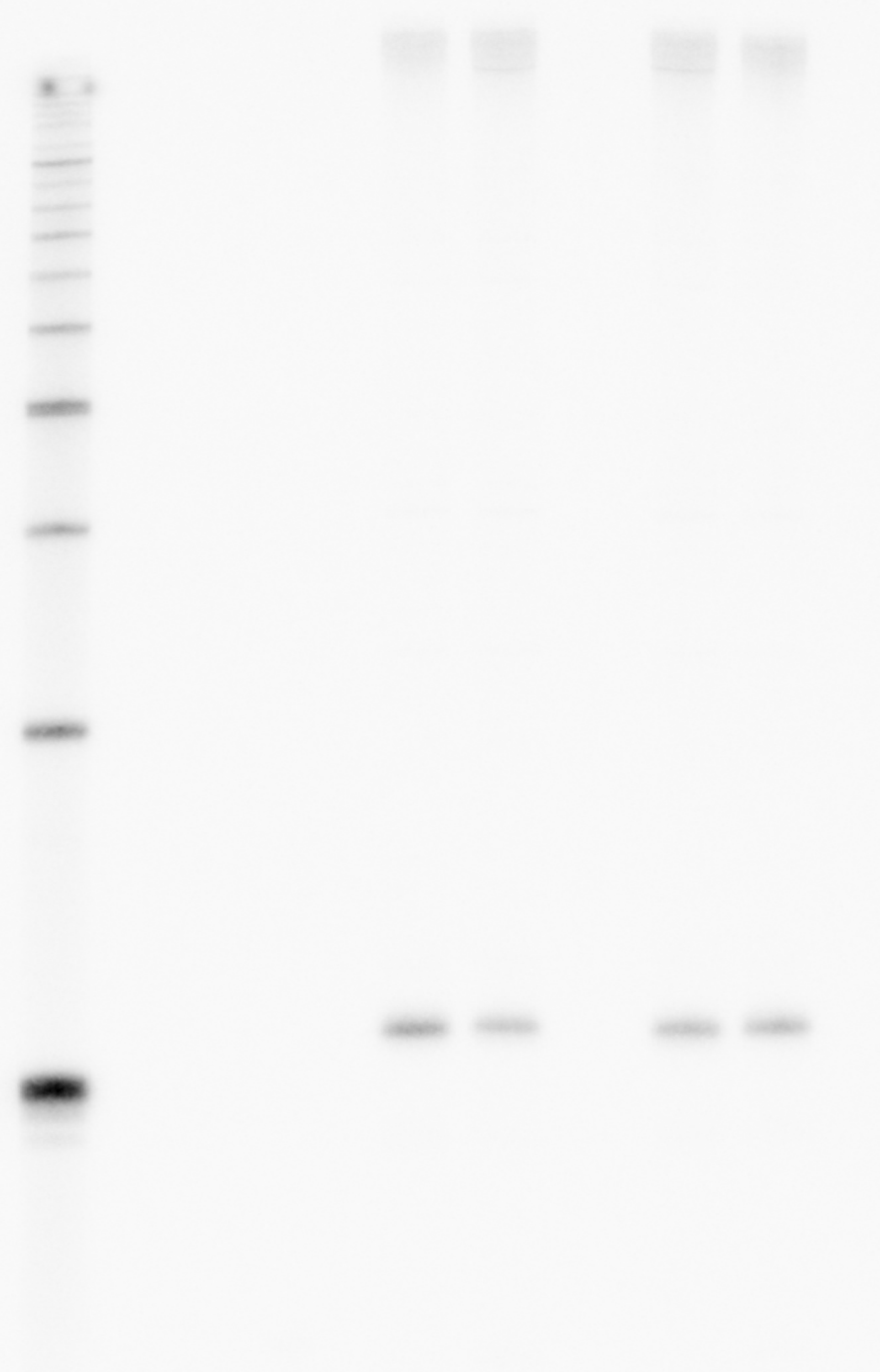


1 2 3 4 5 6 [lane]


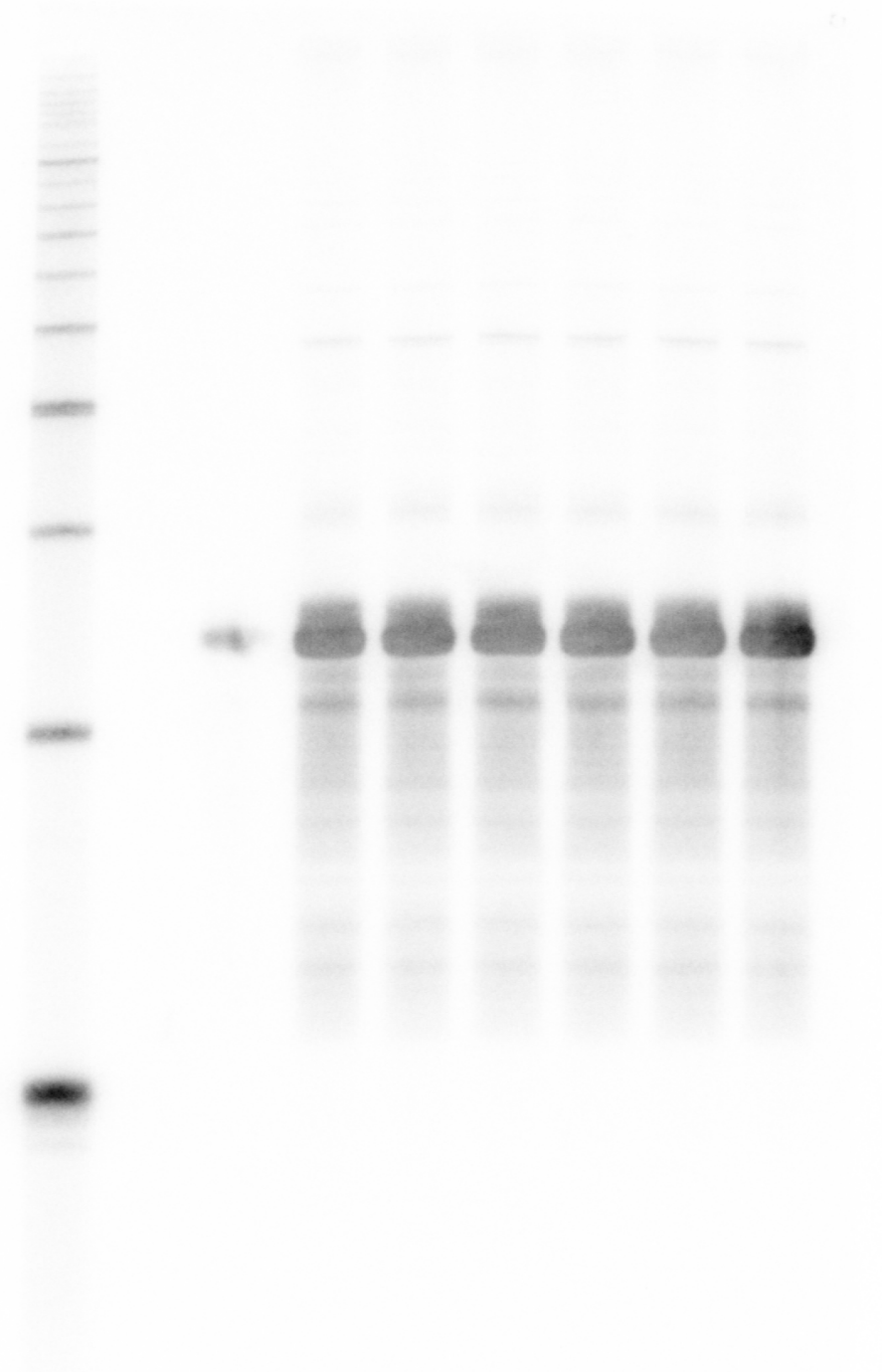


1 2 3 4 5 6 [lane]

CarZ (KPO-2482) 5S (KPO-0243)


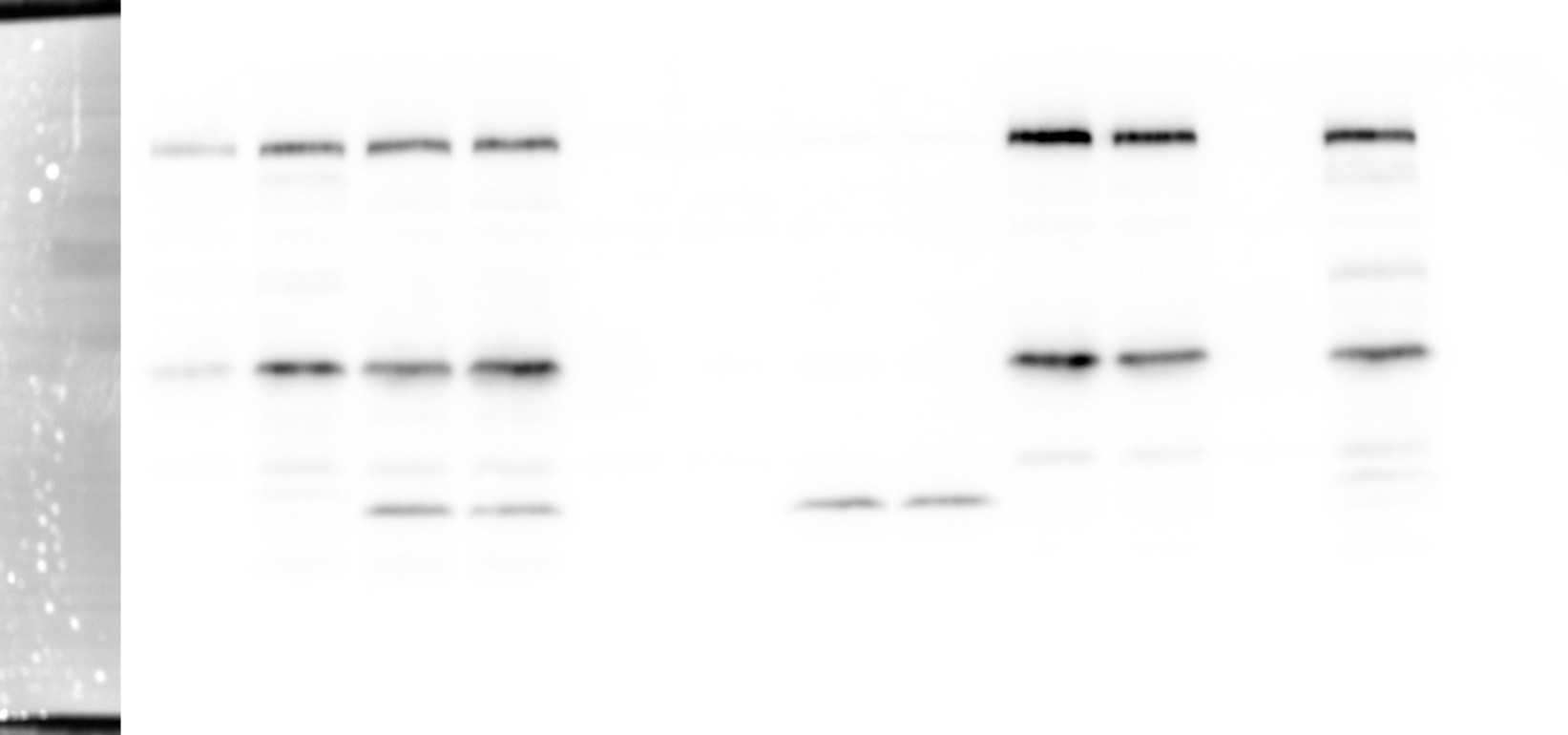

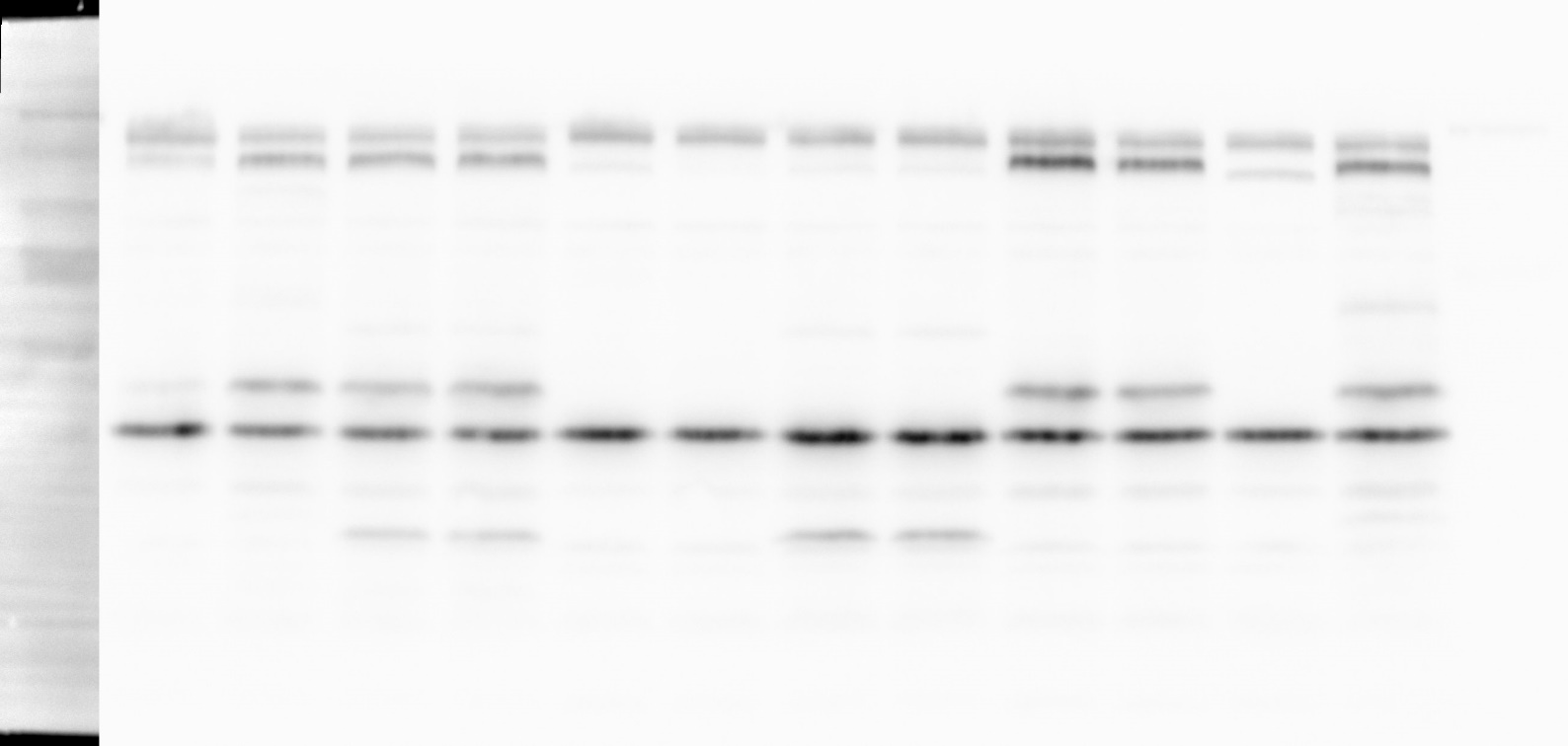


kDa 130

100

70

1 2 3 4 5 6 7 8 9 10 11 12 [lane]

CarB

55

CarA

40

α-FLAG

kDa 130

100

70

55

1 2 3 4 5 6 7 8 9 10 11 12 [lane]

40

RNAP

α-RNAP

1 2 3 4 5 6 7 8 9 10 11 12 [lane]


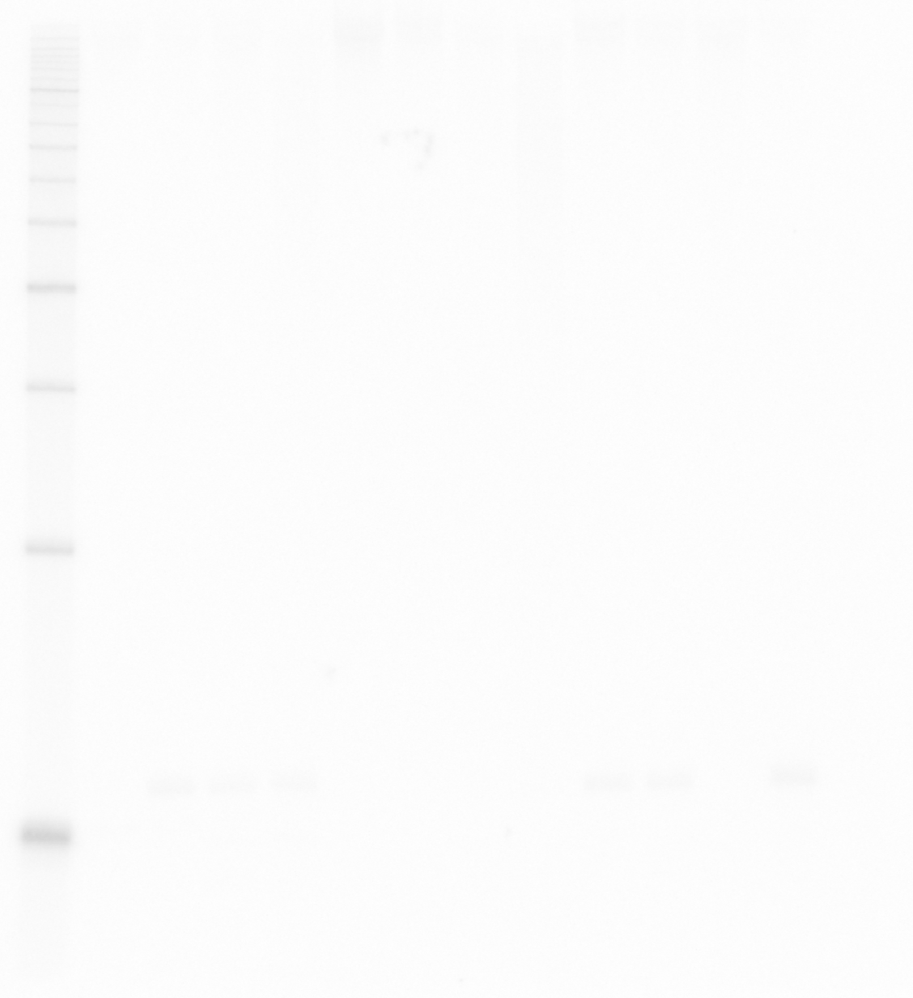

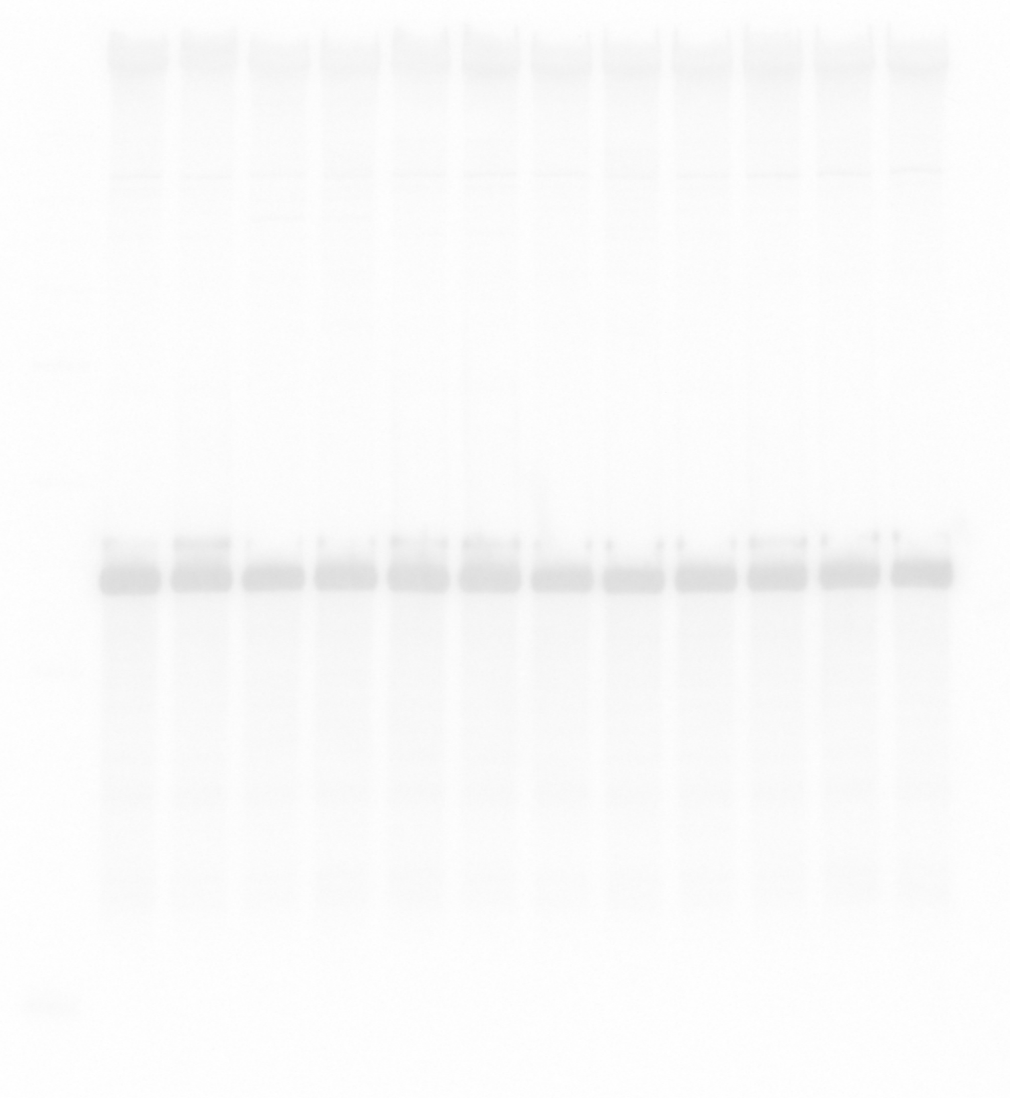


1 2 3 4 5 6 7 8 9 10 11 12 [lane]

CarZ (KPO-2482) 5S (KPO-0243)


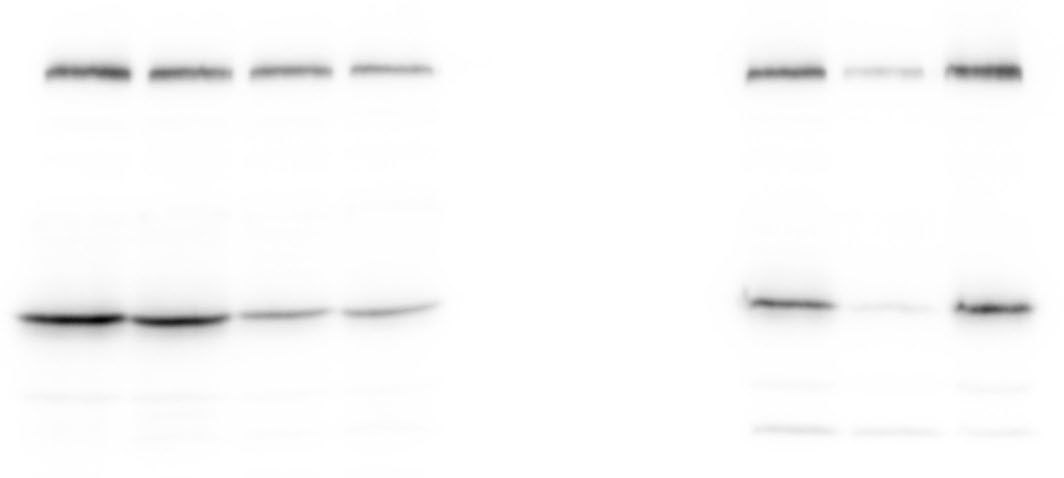


CarB

[lane] 1 2 3

CarA


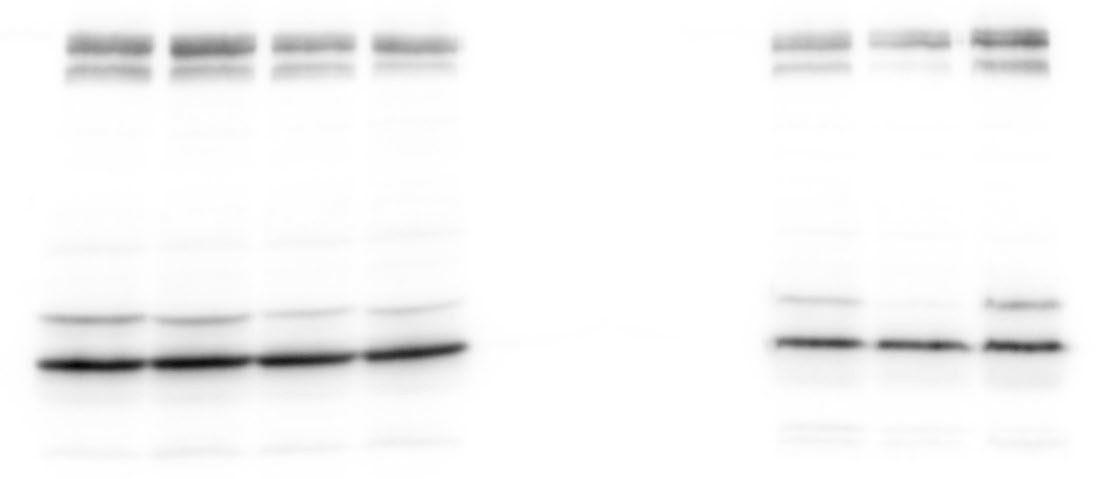


[lane] 1 2 3

RNAP

α-FLAG α-RNAP


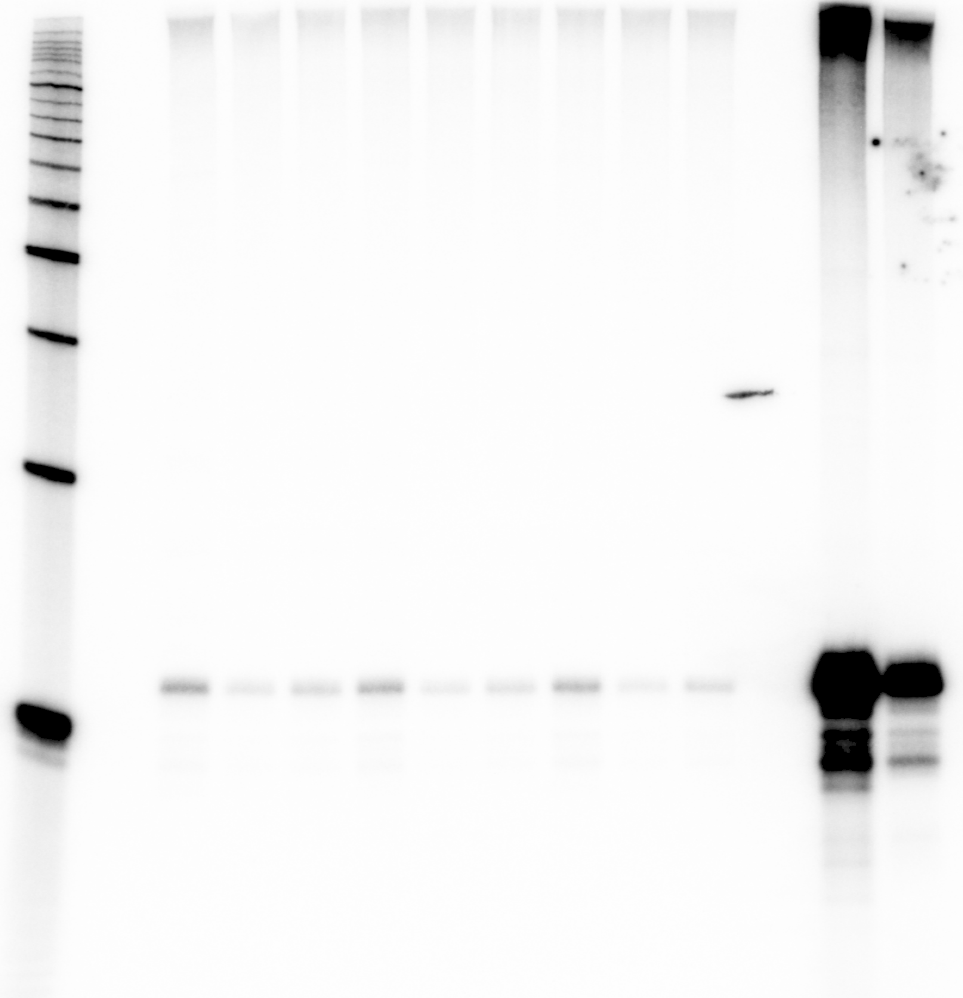


[lane] 1 2 3

Native CarZ (KPO-0821)


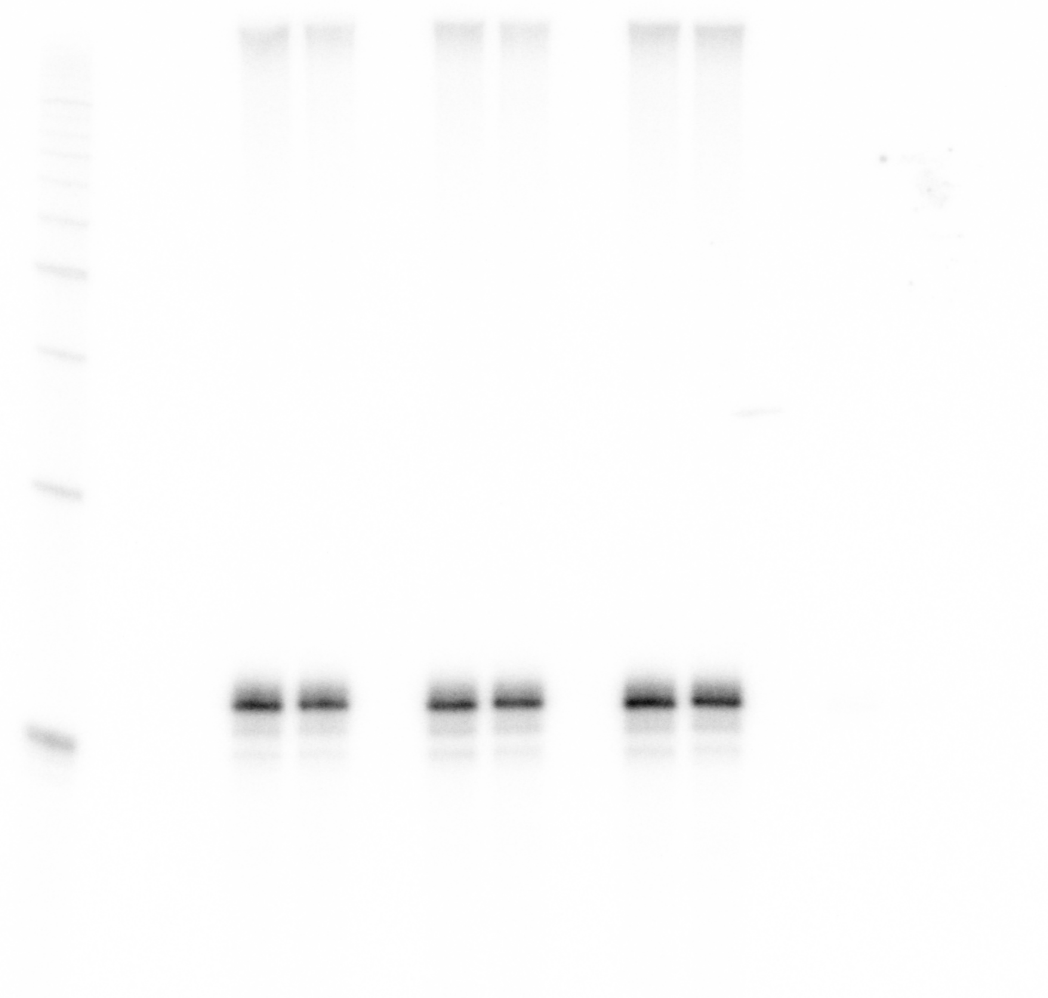


[lane] 1 2 3

Regulator CarZ (KPO-5822)


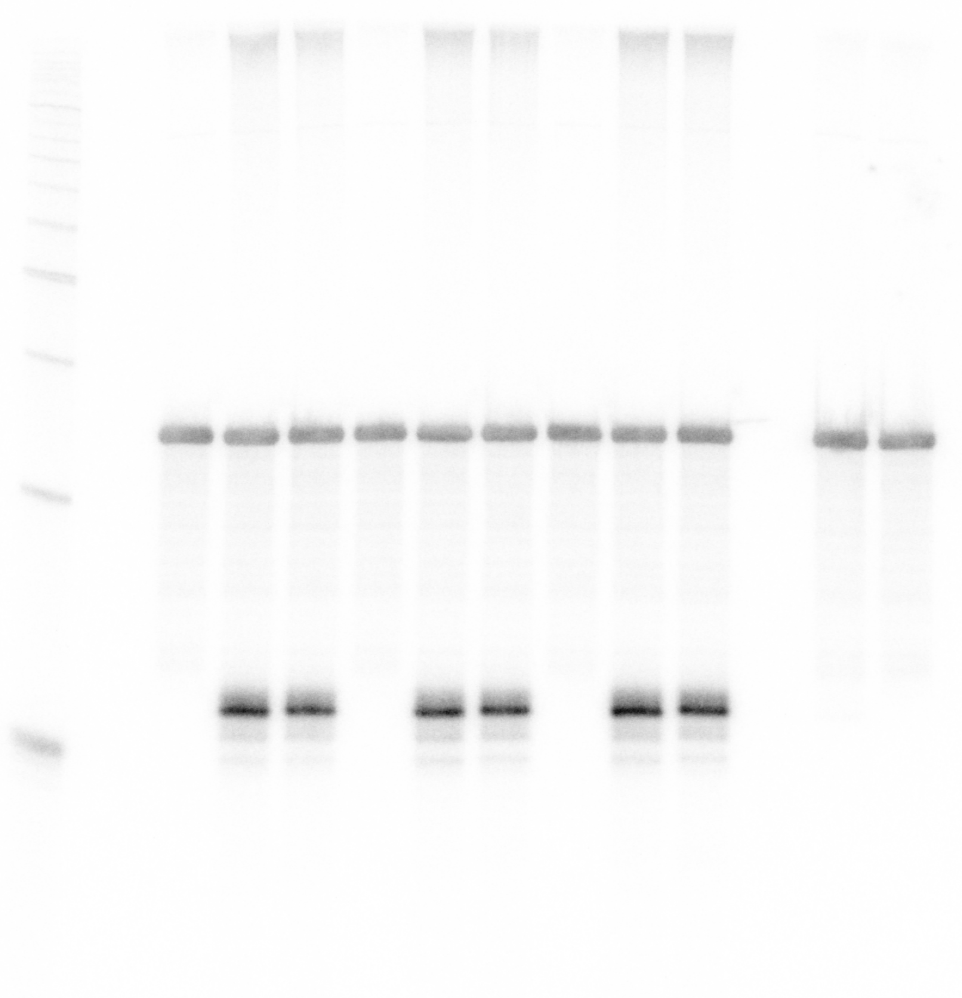


[lane] 1 2 3

5S (KPO-0243)


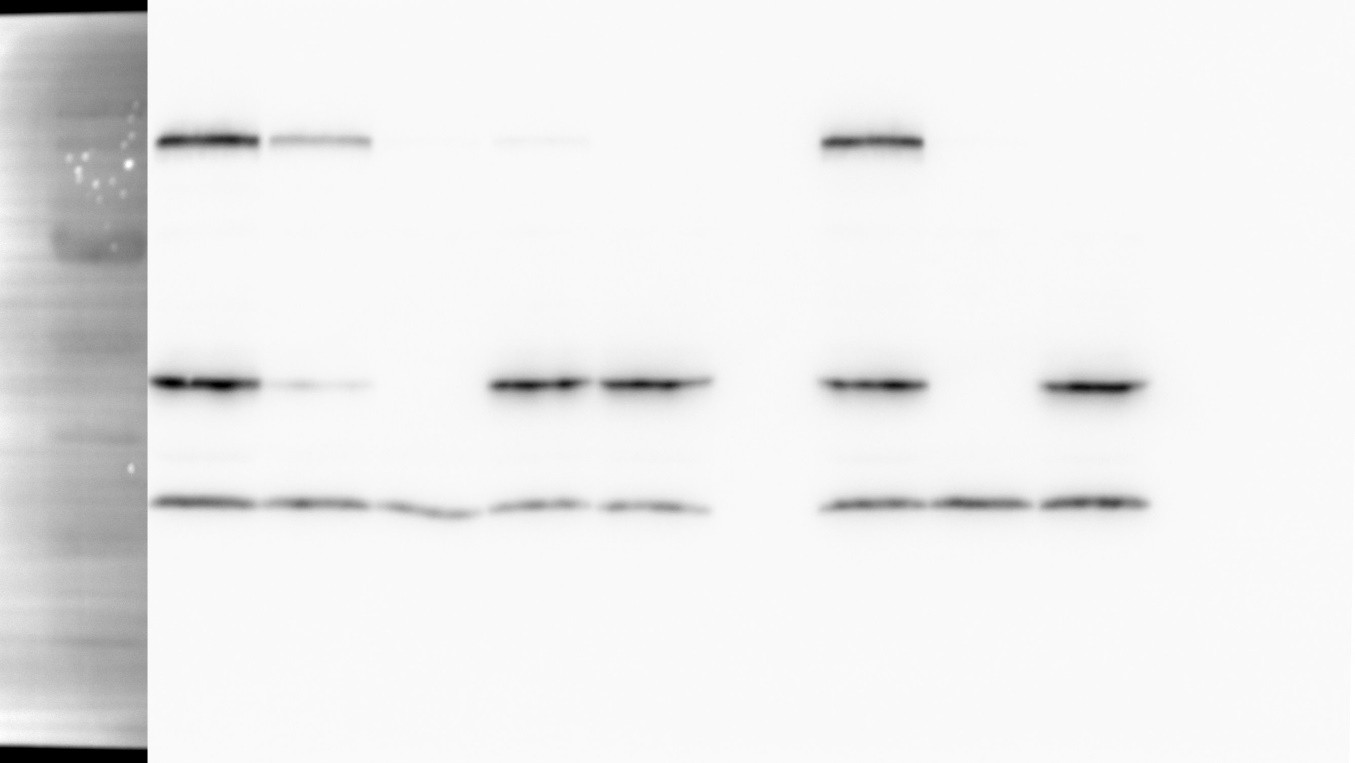


kDa 130

100

70

1 2 3 [lane]

CarB

55

CarA

40

35

α-FLAG


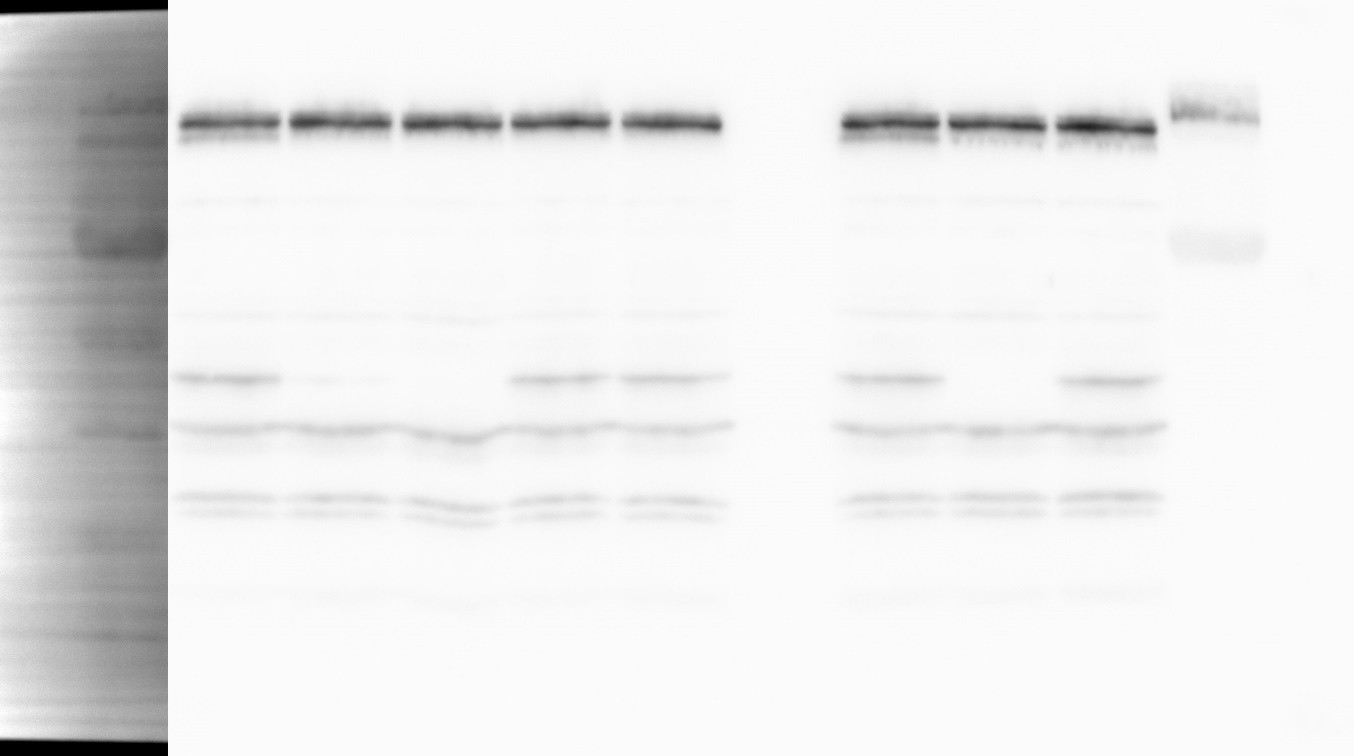


1 2 3

kDa 130

100

70

[lane]

RNAP

55

40

35

α-RNAP

1 2 3 [lane]


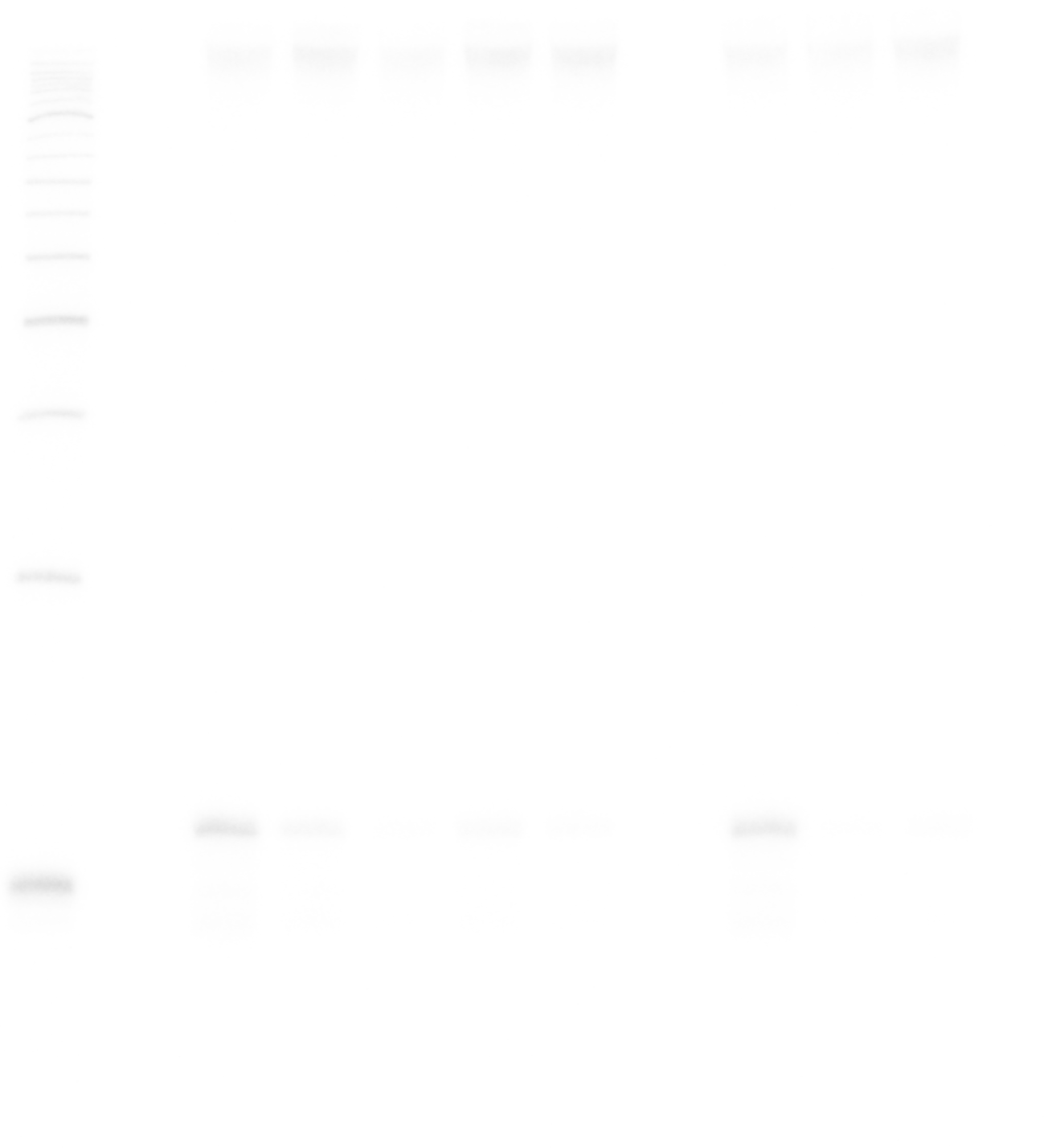

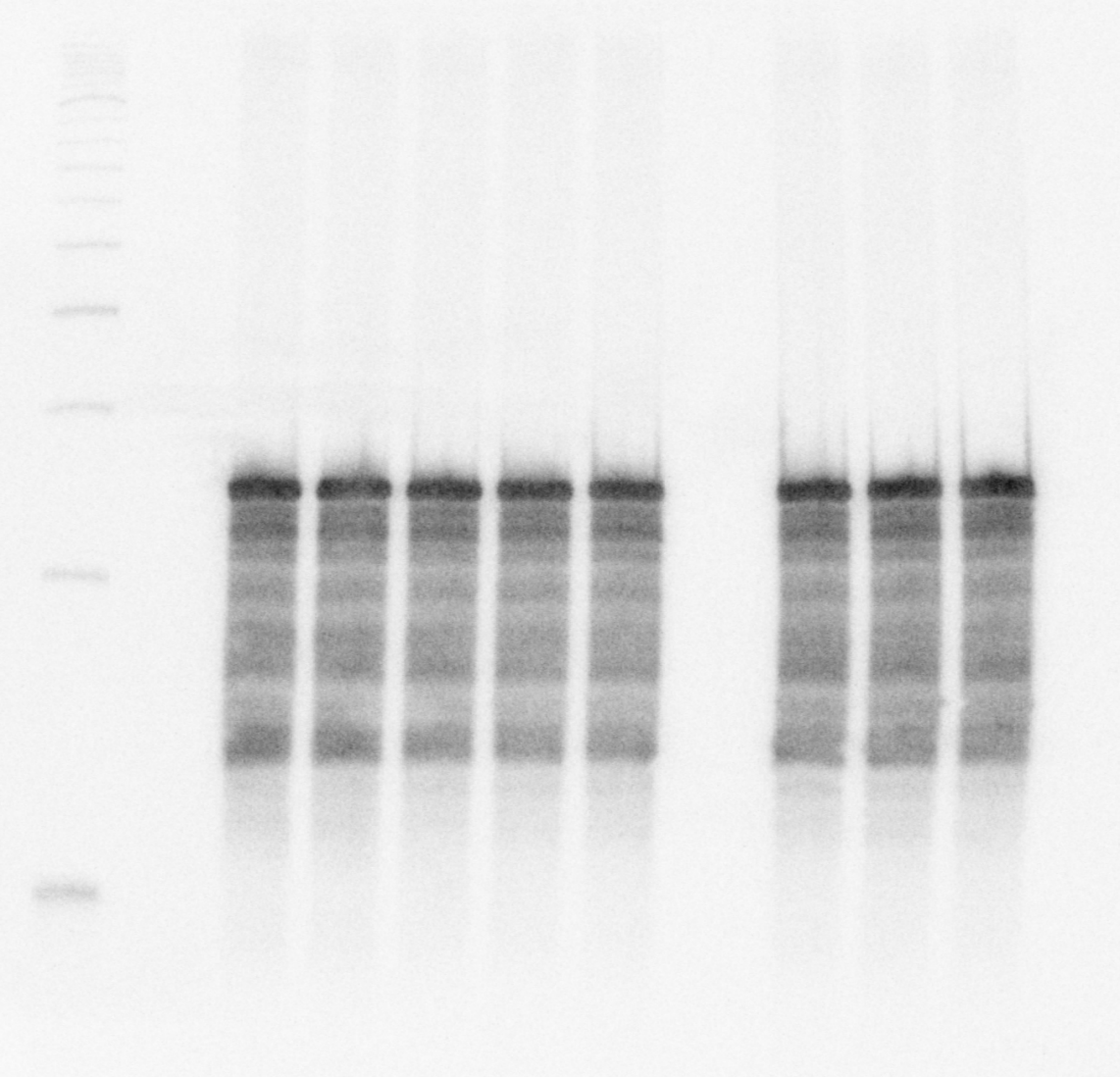


1 2 3 [lane]

CarZ (KPO-0821) 5S (KPO-0243)
