# Supplementary material for: Gene autoregulation by 3’ UTR-derived bacterial small RNAs
Source: eLife. 2020 Aug 3;9:e58836. doi: 10.7554/eLife.58836 (PMC7398697; doi:10.7554/eLife.58836)
Supplement: Figure 7—figure supplement 1—source data 1. [file elife-58836-fig7-figsupp1-data1.docx]

Figure 7 – figure supplement 1B

Data: (fluorescence / OD600) - autofluorescence

| **target** | **sRNA** | **rep 1** | **rep 2** | **rep 3** |
| --- | --- | --- | --- | --- |
| ***carA*** | pCtrl | 361858.314 | 353026.158 | 376882.269 |
|  | pCarZ | 5934.69912 | 5001.14263 | 4187.01872 |
|  | pCarZ M1 | 474552.61 | 507863.509 | 494835.238 |
| ***carA* M1** | pCtrl | 376290.49 | 370151.581 | 373293.958 |
|  | pCarZ | 329619.382 | 339412.4 | 359741.329 |
|  | pCarZ M1 | 8554.77993 | 9839.31999 | 8020.39223 |
| ***carB*** | pCtrl | 213977.98 | 216988.176 | 218533.447 |
|  | pCarZ | 7774.23889 | 8082.55741 | 7674.90904 |
|  | pCarZ M1 | 251059.148 | 269696.104 | 265688.202 |
| ***carB* M1** | pCtrl | 173751.276 | 186568.496 | 176131.298 |
|  | pCarZ | 167966.978 | 175854.393 | 171251.735 |
|  | pCarZ M1 | 7972.894 | 8041.1428 | 8006.85709 |

Figure 7 – figure supplement 1C

1 2 3 4 [lane]


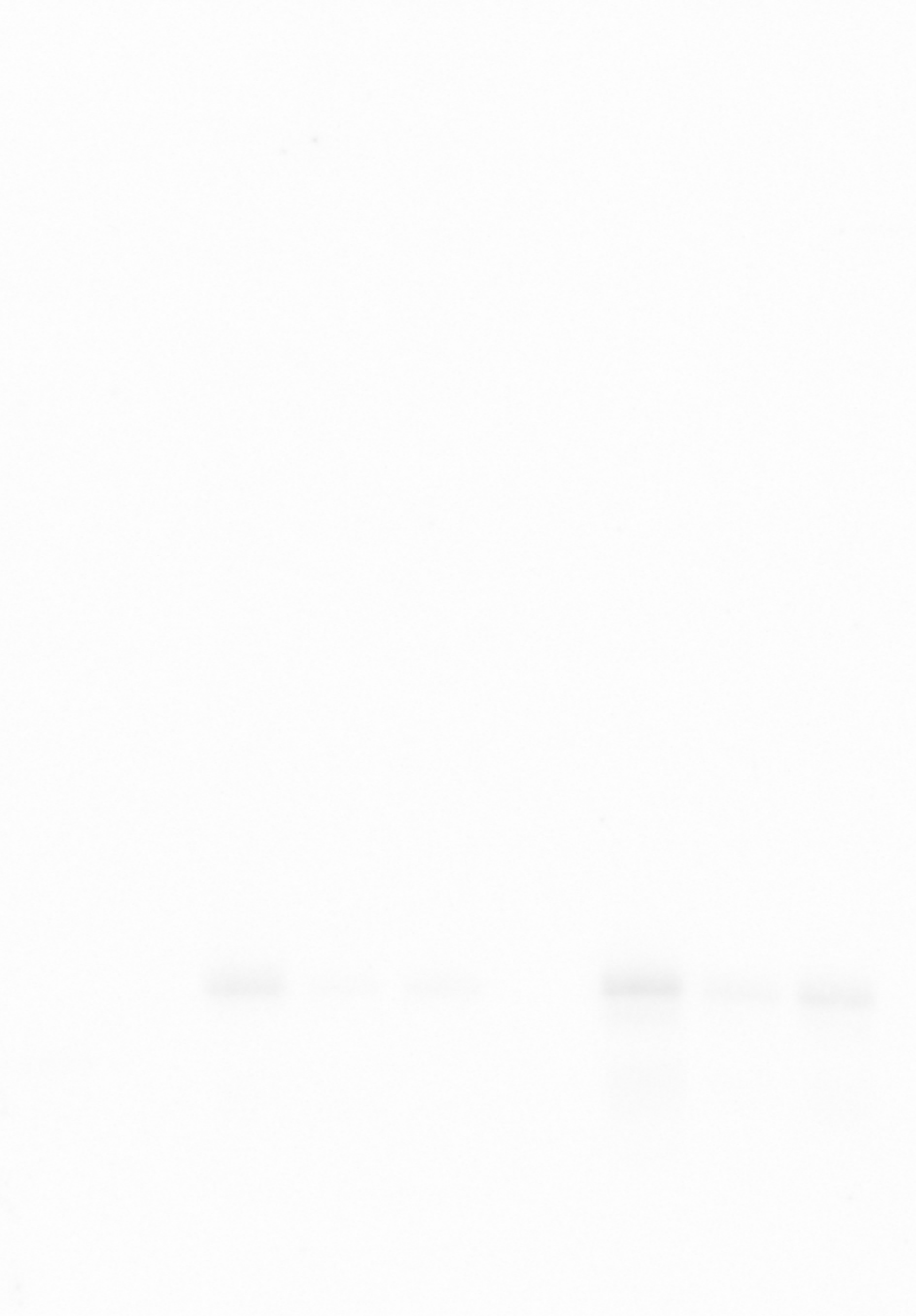


1 2 3 4 [lane]


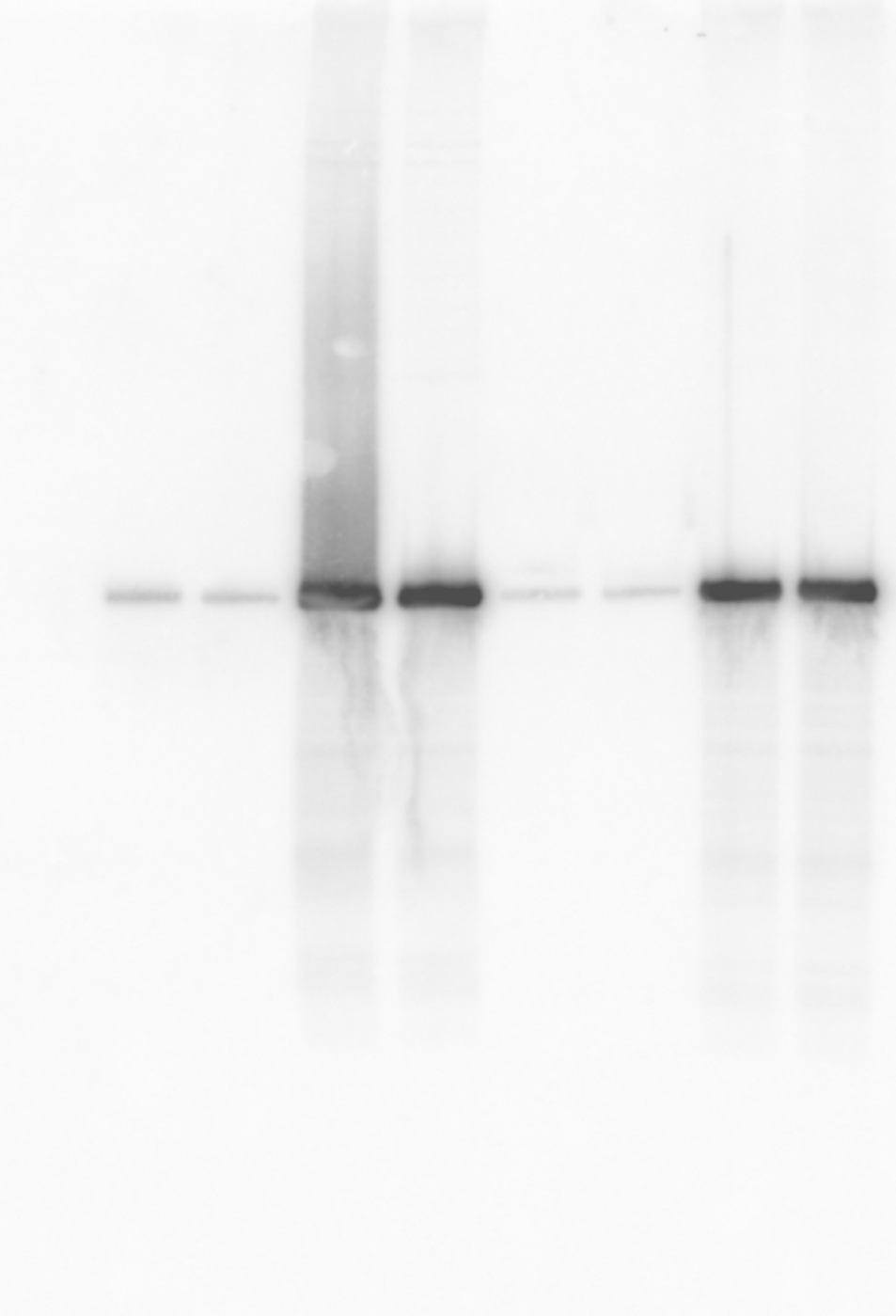


CarZ (KPO-0821) 5S (KPO-0243)

Data: (fluorescence / OD600) - autofluorescence

| **sRNA** | **rep 1** | **rep 2** | **rep 3** |
| --- | --- | --- | --- |
| **pCtrl** | 302626.046 | 241331.163 | 313665.23 |
| **pCarZ** | 304283.345 | 241878.643 | 324403.628 |
| **pCarZ M1** | 303796.737 | 236465.074 | 310800.844 |

Figure 7 – figure supplement 1E


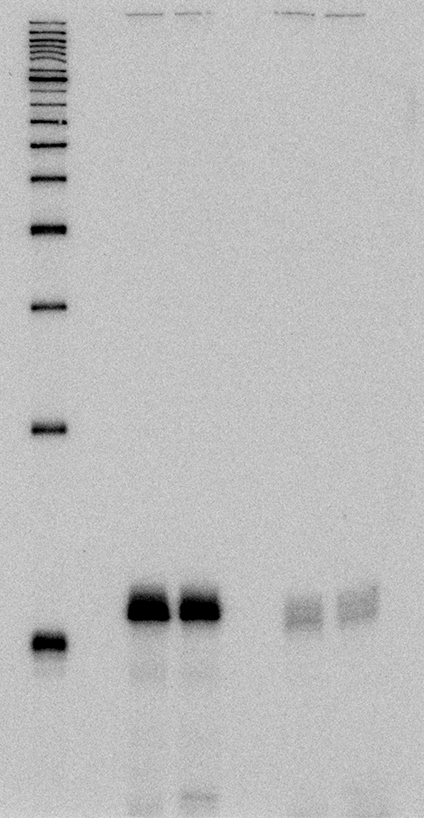


1 2 3 4

5 6 [lane]


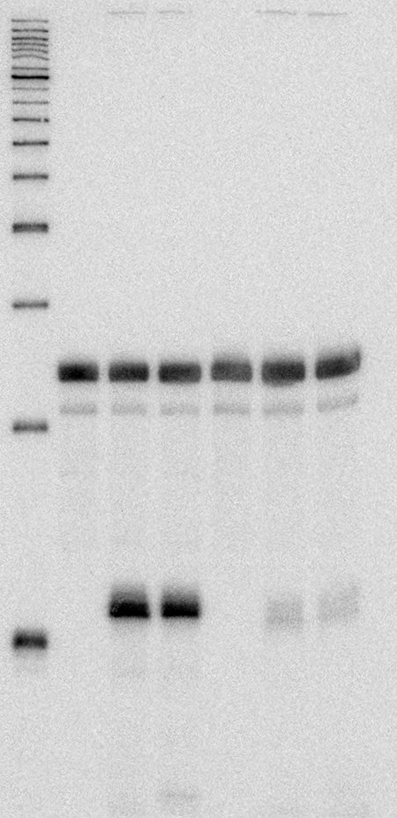


1 2 3 4

5 6 [lane]

CarZ (KPO-2482) 5S (KPO-0243)
