# Supplementary material for: Gene autoregulation by 3’ UTR-derived bacterial small RNAs
Source: eLife. 2020 Aug 3;9:e58836. doi: 10.7554/eLife.58836 (PMC7398697; doi:10.7554/eLife.58836)
Supplement: Figure 7—figure supplement 2—source data 1. [file elife-58836-fig7-figsupp2-data1.docx]

# Source data for Figure 7 – figure supplement 2

**Figure 7 – figure supplement 2A**

Data: *carA* transcript levels determined by qRT-PCR, fold change relative to t = 0 min

|  | **pCtrl** | | | | | **pCarZ** | | | | |
| --- | --- | --- | --- | --- | --- | --- | --- | --- | --- | --- |
| **[min]** | rep 1 | rep 2 | rep 3 | mean | SD | rep 1 | rep 2 | rep 3 | mean | SD |
| **0** | 1.000 | 1.000 | 1.000 | 1.000 | 0.000 | 1.000 | 1.000 | 1.000 | 1.000 | 0.000 |
| **1** | 0.411 | 0.467 | 0.405 | 0.427 | 0.008 | 0.094 | 0.084 | 0.073 | 0.084 | 0.008 |
| **2** | 0.320 | 0.258 | 0.285 | 0.287 | 0.006 | 0.037 | 0.051 | 0.043 | 0.044 | 0.006 |
| **3** | 0.202 | 0.164 | 0.202 | 0.189 | 0.001 | 0.032 | 0.031 | 0.033 | 0.032 | 0.001 |
| **4** | 0.113 | 0.101 | 0.138 | 0.117 | 0.001 | 0.020 | 0.022 | 0.020 | 0.021 | 0.001 |

# Figure 7 – figure supplement 2B

Data: *carB* transcript levels determined by qRT-PCR, fold change relative to t = 0 min

|  | **pCtrl** | | | | | **pCarZ** | | | | |
| --- | --- | --- | --- | --- | --- | --- | --- | --- | --- | --- |
| **[min]** | rep 1 | rep 2 | rep 3 | mean | SD | rep 1 | rep 2 | rep 3 | mean | SD |
| **0** | 1.000 | 1.000 | 1.000 | 1.000 | 0.000 | 1.000 | 1.000 | 1.000 | 1.000 | 0.000 |
| **1** | 0.581 | 0.691 | 0.690 | 0.654 | 0.061 | 0.445 | 0.297 | 0.360 | 0.367 | 0.061 |
| **2** | 0.354 | 0.364 | 0.482 | 0.400 | 0.009 | 0.178 | 0.158 | 0.161 | 0.166 | 0.009 |
| **3** | 0.283 | 0.259 | 0.433 | 0.325 | 0.041 | 0.197 | 0.097 | 0.132 | 0.142 | 0.041 |
| **4** | 0.177 | 0.183 | 0.281 | 0.214 | 0.006 | 0.102 | 0.097 | 0.112 | 0.104 | 0.006 |
