# Supplementary material for: Gene autoregulation by 3’ UTR-derived bacterial small RNAs
Source: eLife. 2020 Aug 3;9:e58836. doi: 10.7554/eLife.58836 (PMC7398697; doi:10.7554/eLife.58836)
Supplement: Figure 8—source data 1. [file elife-58836-fig8-data1.docx]

# Figure 8A

kDa 70


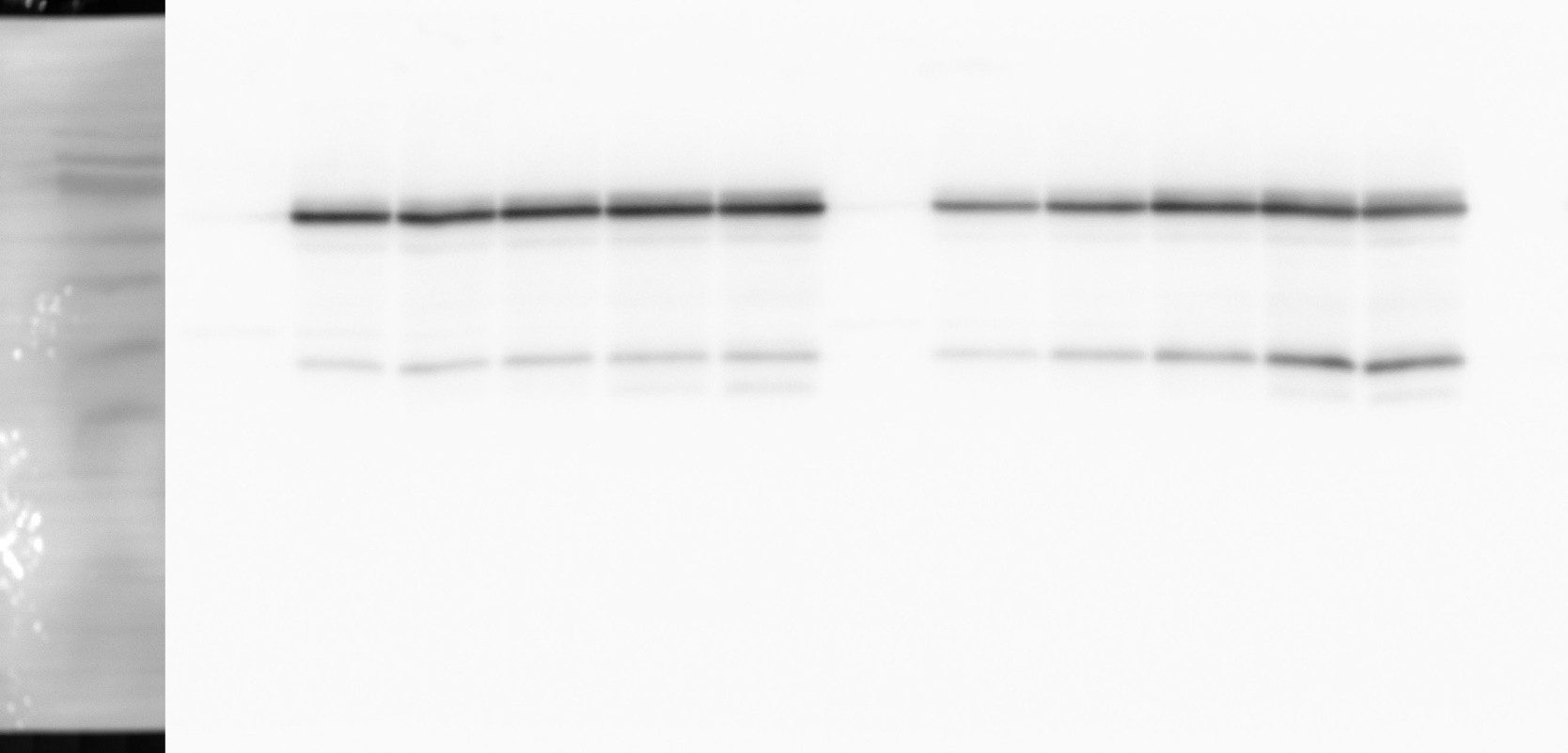

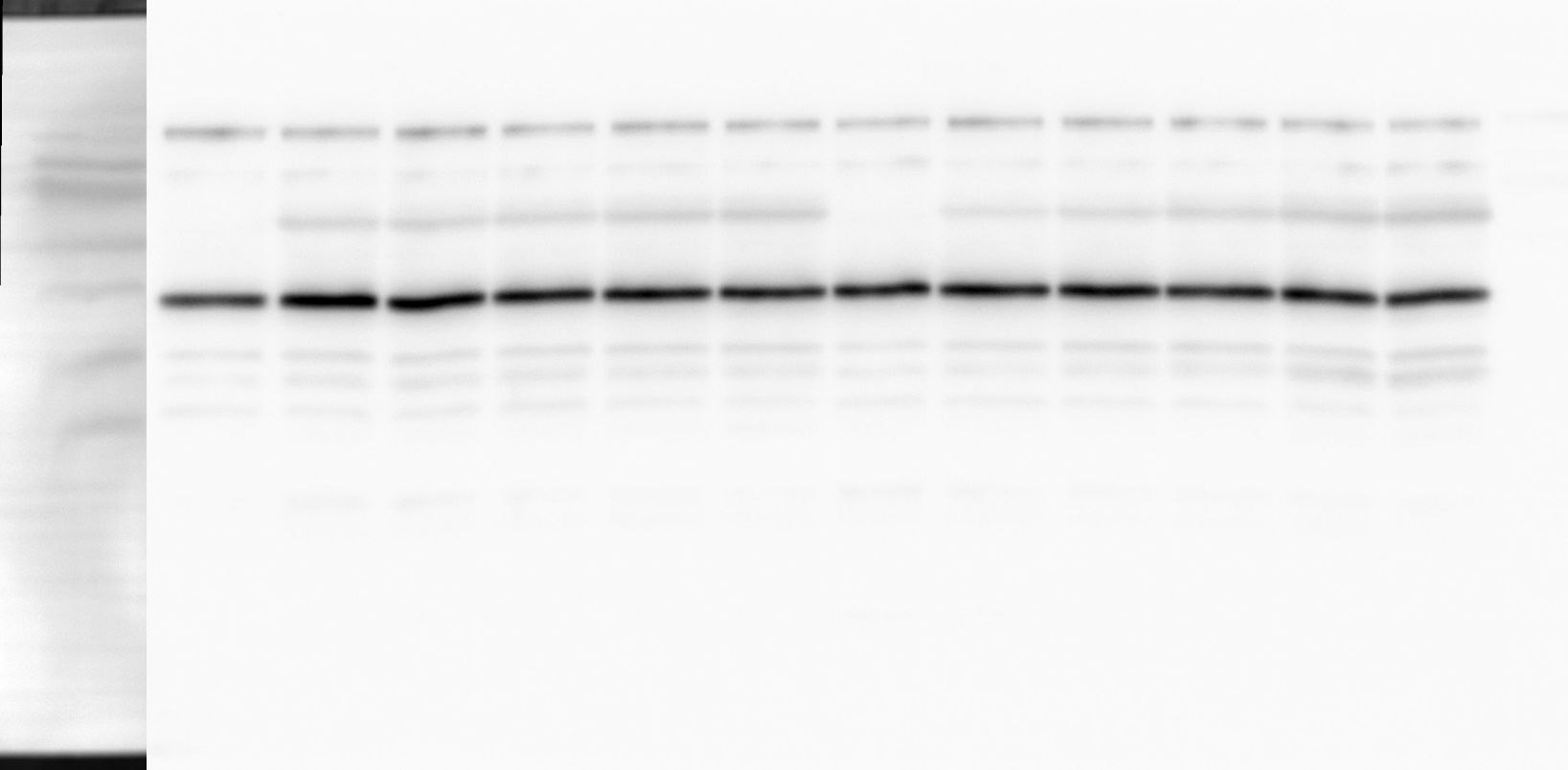


55

40

35

25

1 2 3 4 5 6 7 8 9 10 11 12 [lane]

OppA OppB

α-FLAG

kDa 70

55

40

35

25

RNAP

1 2 3 4 5 6 7 8 9 10 11 12 [lane]

α-RNAP

1 2 3 4 5 6 7 8 9 10 11 12 [lane]


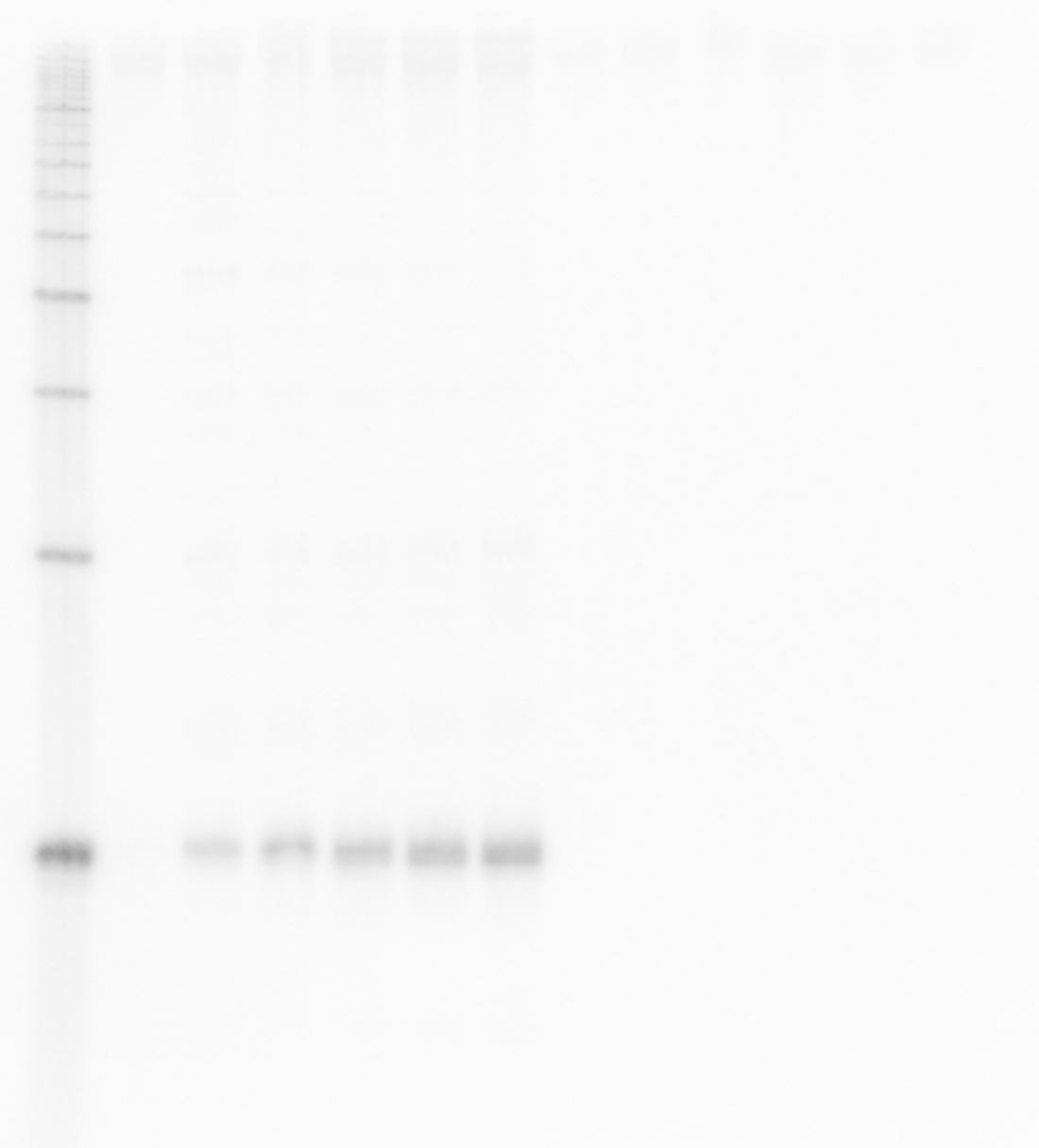


1 2 3 4 5 6 7 8 9 10 11 12


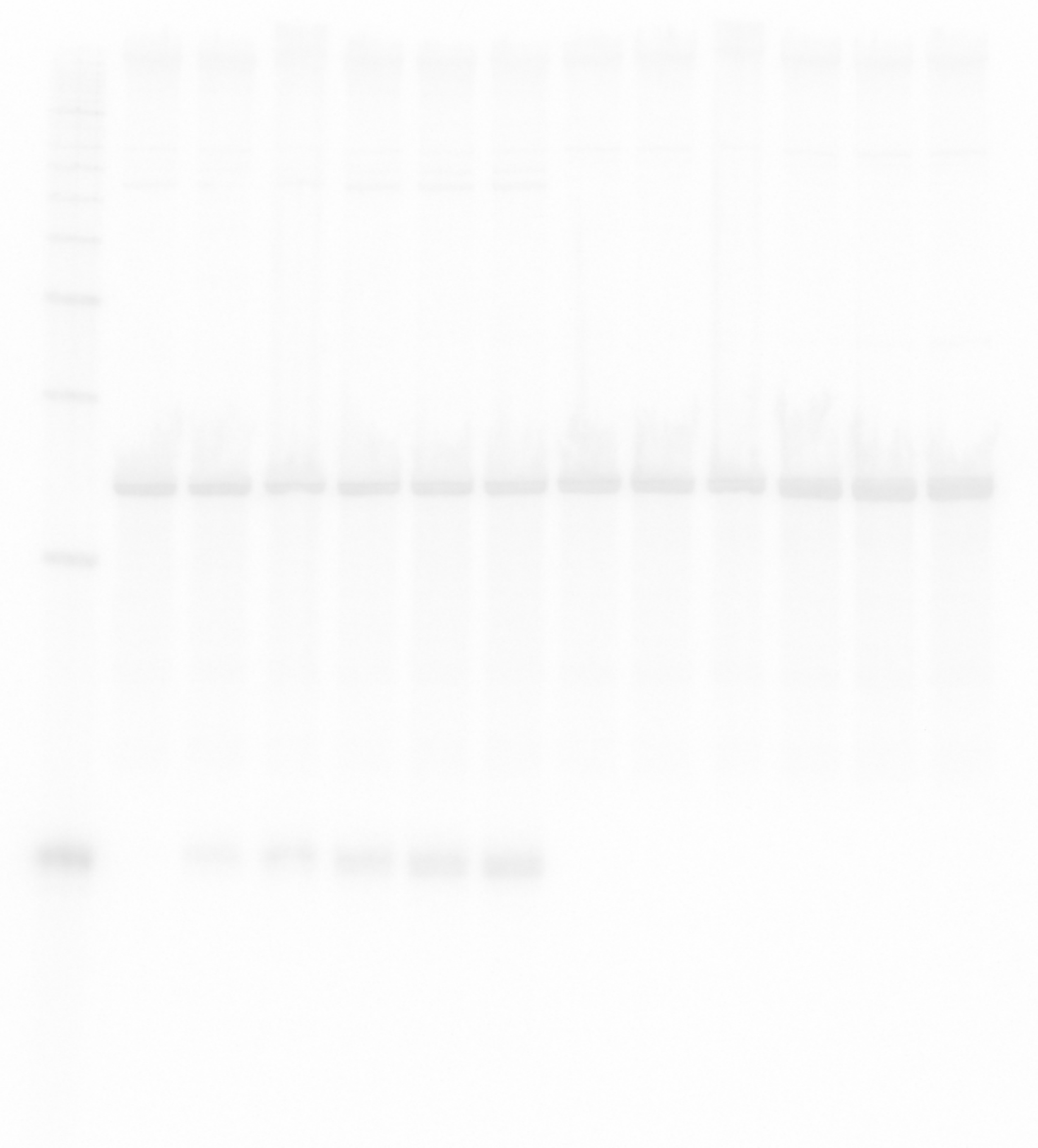


[lane]

OppZ (KPO-0845) 5S (KPO-0243)

Data:

- OppA and RNAP protein levels quantified from 3 biological replicates on Western blot
- OppA normalized to RNAP
- fold change relative to t = 180 min in the wild-type

|  | **wild-type** | | | | | **ΔoppZ** | | | | |
| --- | --- | --- | --- | --- | --- | --- | --- | --- | --- | --- |
| **[min]** | rep 1 | rep 2 | rep 3 | mean | SD | rep 1 | rep 2 | rep 3 | mean | SD |
| **0** | 0.0011 | 0.0087 | 0.0114 | 0.0070 | 0.0131 | 0.0090 | 0.0127 | 0.0385 | 0.0201 | 0.0131 |
| **15** | 0.3183 | 0.4963 | 0.4379 | 0.4175 | 0.1319 | 0.1914 | 0.2795 | 0.5048 | 0.3252 | 0.1319 |
| **30** | 0.3649 | 0.5460 | 0.9571 | 0.6227 | 0.2310 | 0.2538 | 0.4504 | 0.8115 | 0.5052 | 0.2310 |
| **60** | 0.4143 | 0.8055 | 1.0480 | 0.7559 | 0.1971 | 0.5028 | 0.8228 | 0.9760 | 0.7672 | 0.1971 |
| **120** | 0.6499 | 0.8521 | 1.1490 | 0.8837 | 0.2178 | 0.9257 | 0.7025 | 1.2337 | 0.9540 | 0.2178 |
| **180** | 1.0000 | 1.0000 | 1.0000 | 1.0000 | 0.3215 | 1.2720 | 0.5812 | 1.2541 | 1.0358 | 0.3215 |

# Figure 8C

Data:

- OppB and RNAP protein levels quantified from 3 biological replicates on Western blot
- OppB normalized to RNAP
- fold change relative to t = 180 min in the wild-type

|  | **wild-type** | | | | | **ΔoppZ** | | | | |
| --- | --- | --- | --- | --- | --- | --- | --- | --- | --- | --- |
| **[min]** | rep 1 | rep 2 | rep 3 | mean | SD | rep 1 | rep 2 | rep 3 | mean | SD |
| **0** | 0.0050 | 0.0050 | 0.0050 | 0.0050 | 0.0006 | 0.0117 | 0.0131 | 0.0122 | 0.0123 | 0.0006 |
| **15** | 0.0821 | 0.2557 | 0.1842 | 0.1740 | 0.0120 | 0.2476 | 0.2765 | 0.2671 | 0.2637 | 0.0120 |
| **30** | 0.1451 | 0.4694 | 0.4680 | 0.3608 | 0.1037 | 0.4994 | 0.7493 | 0.6642 | 0.6376 | 0.1037 |
| **60** | 0.3246 | 0.6871 | 0.6299 | 0.5472 | 0.3920 | 1.2270 | 2.1251 | 1.3819 | 1.5780 | 0.3920 |
| **120** | 0.6700 | 0.7542 | 0.8692 | 0.7645 | 1.4678 | 2.5939 | 5.2683 | 1.8502 | 3.2375 | 1.4678 |
| **180** | 1.0000 | 1.0000 | 1.0000 | 1.0000 | 1.1140 | 3.9203 | 5.6435 | 2.9495 | 4.1711 | 1.1140 |
