# Supplementary material for: Gene autoregulation by 3’ UTR-derived bacterial small RNAs
Source: eLife. 2020 Aug 3;9:e58836. doi: 10.7554/eLife.58836 (PMC7398697; doi:10.7554/eLife.58836)
Supplement: Figure 8—figure supplement 1—source data 1. [file elife-58836-fig8-figsupp1-data1.docx]

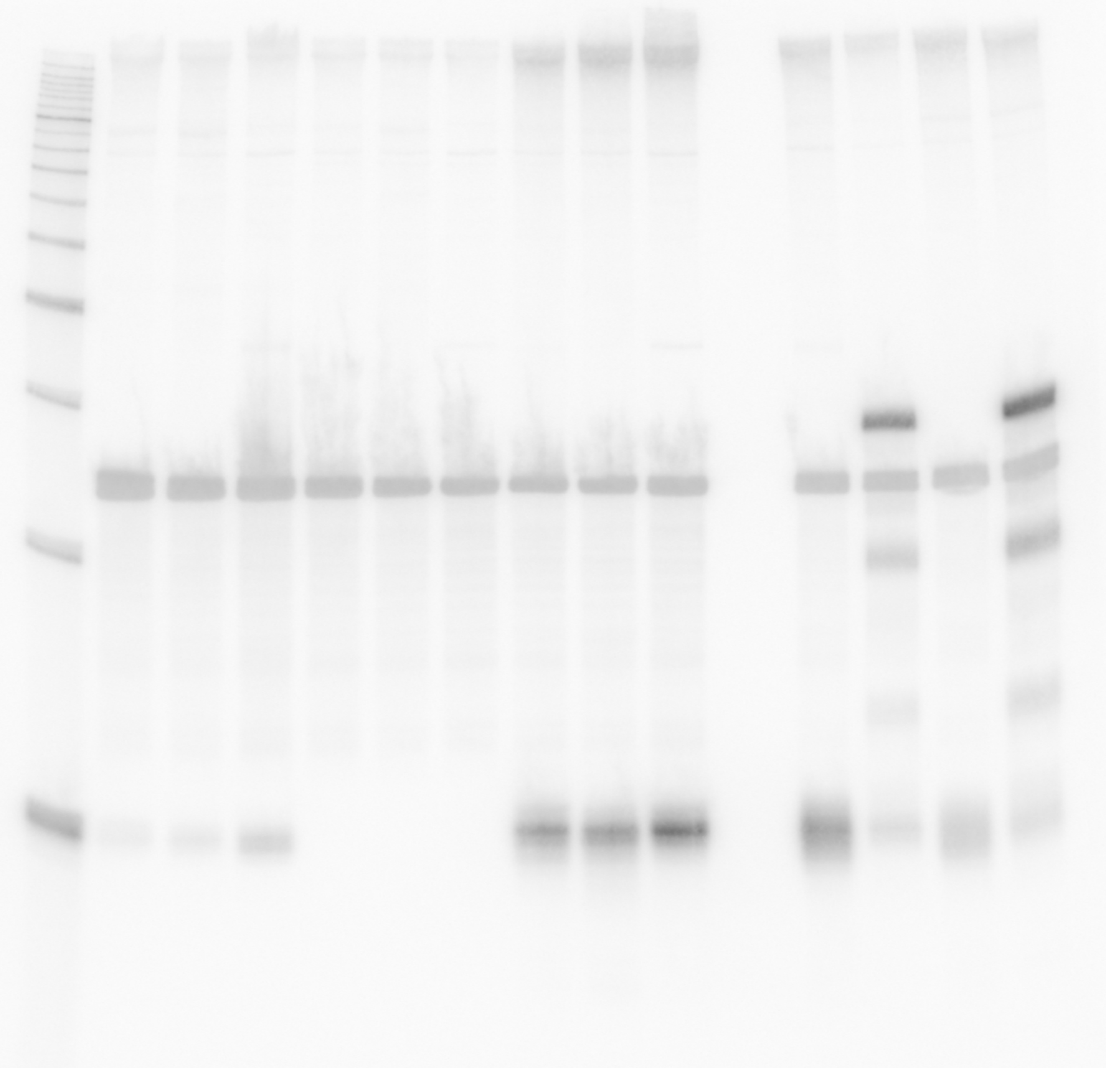


1 2 3 4 5 6 7 8 9 [lane]

# Figure 8 – figure supplement 1A


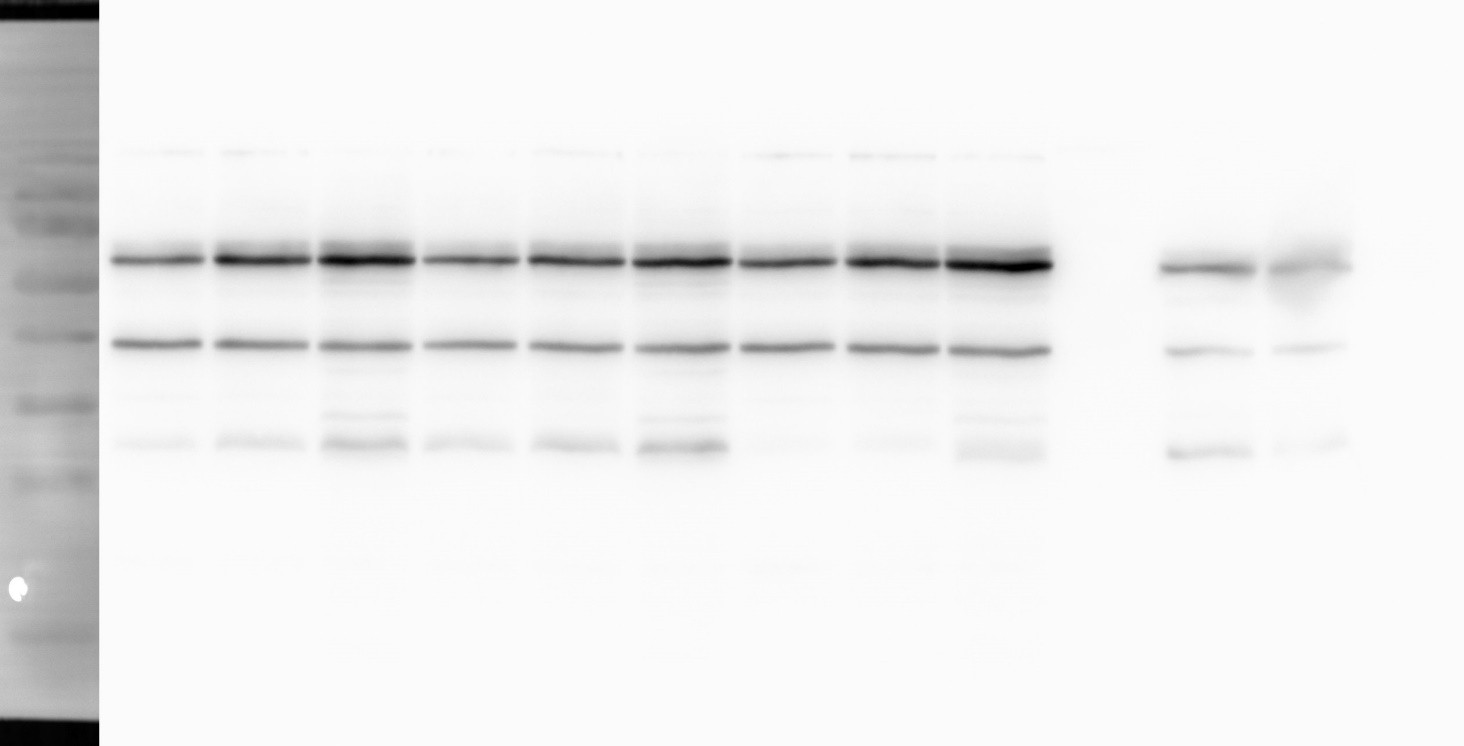


kDa 70

55

40

1 2 3 4 5 6 7 8 9 [lane]

OppA

35

25

OppB

α-FLAG


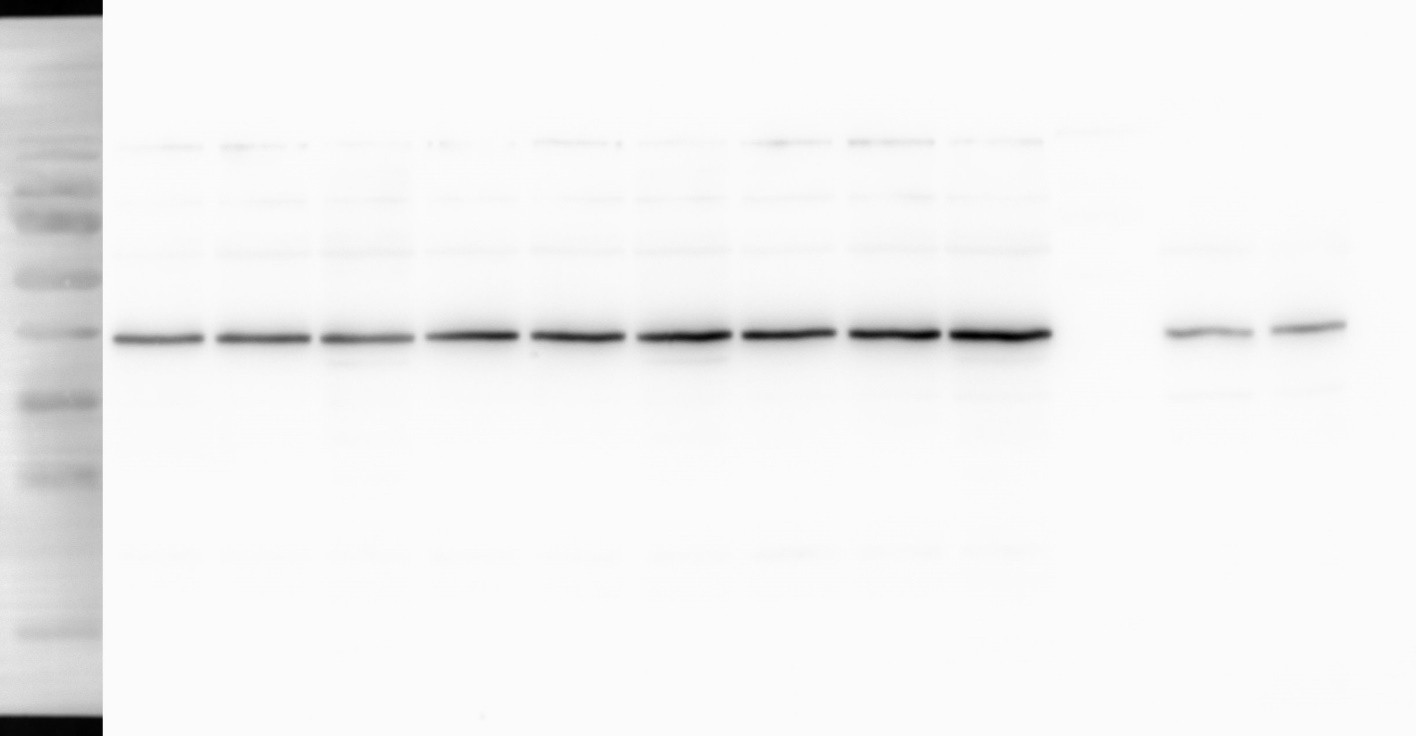


kDa 70

55

40

1 2 3 4 5 6 7 8 9 [lane]

RNAP

35

25

α-RNAP


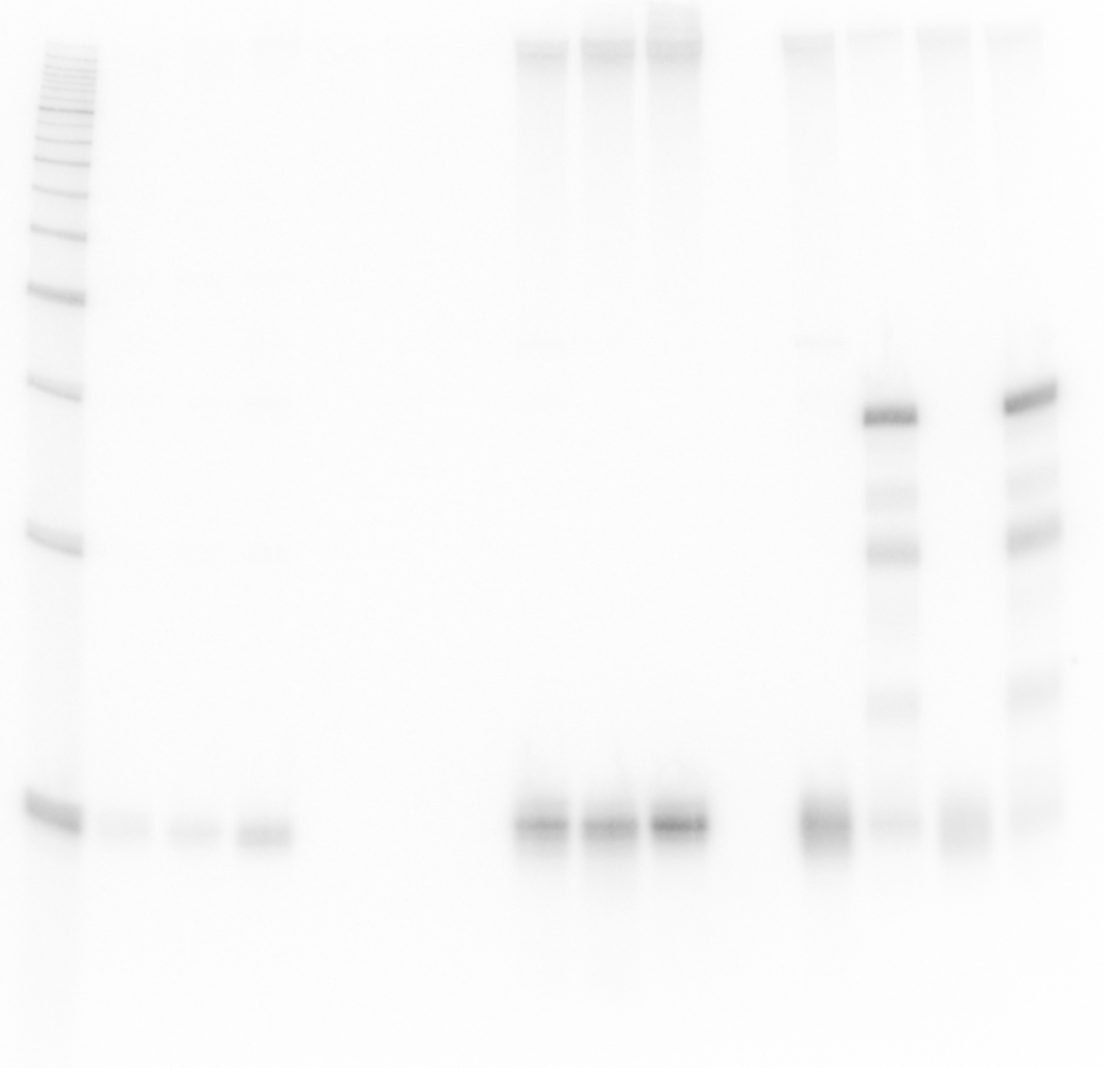


1 2 3 4 5 6 7 8 9 [lane]

OppZ (KPO-0845) 5S (KPO-0243)

Data:

- OppA. OppB and RNAP protein levels quantified from 4 biological replicates on Western blot
- OppA and OppB normalized to RNAP
- fold change in ΔoppZ pCtrl vs. wild-type pCtrl

|  | **OppA** | | | | | | **OppB** | | | | | |
| --- | --- | --- | --- | --- | --- | --- | --- | --- | --- | --- | --- | --- |
| **OD600** | rep 1 | rep 2 | rep 3 | rep 4 | mean | SD | rep 1 | rep 2 | rep 3 | rep 4 | mean | SD |
| **10** | 1.213 | 1.302 | 1.460 | 1.521 | 1.374 | 0.122 | 1.253 | 2.165 | 2.565 | 1.325 | 1.827 | 0.557 |
| **20** | 0.938 | 0.966 | 0.984 | 1.214 | 1.025 | 0.110 | 1.327 | 1.719 | 1.889 | 2.149 | 1.771 | 0.299 |
| **2+3h** | 1.082 | 0.837 | 1.009 | 0.683 | 0.902 | 0.155 | 1.395 | 1.195 | 1.382 | 1.056 | 1.257 | 0.140 |

# Figure 8 – figure supplement 1C

Data:

- OppA. OppB and RNAP protein levels quantified from 4 biological replicates on Western blot
- OppA and OppB normalized to RNAP
- fold change in ΔoppZ pOppZ vs. wild-type pCtrl

|  | **OppA** | | | | | | **OppB** | | | | | |
| --- | --- | --- | --- | --- | --- | --- | --- | --- | --- | --- | --- | --- |
| **OD600** | rep 1 | rep 2 | rep 3 | rep 4 | mean | SD | rep 1 | rep 2 | rep 3 | rep 4 | mean | SD |
| **10** | 1.234 | 1.301 | 1.295 | 1.184 | 1.253 | 0.048 | 0.158 | 0.328 | 0.188 | 0.209 | 0.220 | 0.064 |
| **20** | 0.949 | 0.951 | 0.852 | 1.403 | 1.039 | 0.214 | 0.154 | 0.303 | 0.149 | 0.087 | 0.173 | 0.080 |
| **2+3h** | 1.197 | 1.127 | 1.126 | 1.060 | 1.127 | 0.048 | 0.327 | 0.447 | 0.254 | 0.096 | 0.281 | 0.127 |
