# Supplementary material for: Gene autoregulation by 3’ UTR-derived bacterial small RNAs
Source: eLife. 2020 Aug 3;9:e58836. doi: 10.7554/eLife.58836 (PMC7398697; doi:10.7554/eLife.58836)
Supplement: Supplementary file 4. [file elife-58836-supp4.docx]

**Supplementary File 4A:** Bacterial strains used in this study.

| **Strain** | **Relevant markers/ genotype** | **Reference/ source** |
| --- | --- | --- |
| ***V. cholerae*** |  |  |
| KPS-0014 | C6706 wild-type | (Thelin and Taylor, 1996) |
| KPS-0054 | C6706 Δ*hfq* | (Svenningsen et al., 2009) |
| KPS-0995 | C6706 *hfq*::*hfq*-3XFlag | (Peschek et al., 2019) |
| KPVC10141 | C6706 *rne*-3071 | This study |
| KPVC11302 | C6706 Δ*oppZ* | This study |
| KPVC11709 | C6706 *oppA*::3xFlag *oppB*::3xFlag | This study |
| KPVC11711 | C6706 Δ*oppZ oppA*::3xFlag *oppB*::3xFlag | This study |
| KPVC12422 | C6706 P*oppABCDF*::P*BAD oppA*::3xFlag *oppB*::3xFlag | This study |
| KPVC12424 | C6706 Δ*oppZ* P*oppABCDF*::P*BAD oppA*::3xFlag *oppB*::3xFlag | This study |
| KPVC12898 | C6706 *oppA*::3xFlag *oppB*::3xFlag *oppB* ATC | This study |
| KPVC13205 | C6706 *oppA*::3xFlag *oppB*::3xFlag *oppB* 2nd stop | This study |
| KPVC13207 | C6706 *oppA*::3xFlag *oppB*::3xFlag *oppB* 15th stop | This study |
| KPVC13313 | C6706 *oppA*::3xFlag *oppB*::3xFlag *oppB* 65th stop | This study |
| KPVC13401 | C6706 *oppA*::3xFlag *oppB*::3xFlag *oppB* 115th stop | This study |
| KPVC13403 | C6706 *oppA*::3xFlag *oppB*::3xFlag *oppB* 215th stop | This study |
| KPVC13209 | C6706 *oppA*::3xFlag *oppB*::3xFlag *oppC* ATC | This study |
| KPVC13230 | C6706 *oppA*::3xFlag *oppB*::3xFlag *oppD* ATC | This study |
| KPVC13232 | C6706 *oppA*::3xFlag *oppB*::3xFlag *oppF* CTG | This study |
| KPVC12368 | C6706 *oppA*::3xFlag *oppB*::3xFlag *oppF*::3xFlag | This study |
| KPVC13251 | C6706 *oppA*::3xFlag *oppB*::3xFlag *oppF*::3xFlag *oppB* ATC | This study |
| KPVC13098 | C6706 Δ*hfq oppA*::3xFlag *oppB*::3xFlag | This study |
| KPVC13100 | C6706 Δ*hfq* Δ*oppZ oppA*::3xFlag *oppB*::3xFlag | This study |
| KPVC12872 | C6706 *carA*::3xFlag *carB*::3xFlag | This study |
| KPVC13096 | C6706 P*carAB*::P*BAD carA*::3xFlag *carB*::3xFlag | This study |
| KPVC13447 | C6706 *carA*::3xFlag *carB*::3xFlag *carA* 2nd stop | This study |
| KPVC13449 | C6706 *carA*::3xFlag *carB*::3xFlag *carB* 2nd stop | This study |
| KPVC13243 | C6706 Δ*carZ* | This study |
|  |  |  |
| ***E. coli*** |  |  |
| TOP10 | F- *mcrA* Δ(*mrr-hsdRMS-mcrBC*) φ80*lacZ*ΔM15 Δ*lacX*74 *nupG recA*1  *araD*139 Δ(*ara-leu*)7697 *galE*15 *galK*16 *rpsL*(StrR) *endA1* λ- | Invitrogen |
| KPEC53467 | Top10 Δ*hfq* | This study |
| S17λpir | Δ*lacU*169 (Φ*lacZ*ΔM15), *recA*1, *endA*1, *hsdR*17, *thi*-1, *gyrA*96, *relA*1,  λ*pir* | (Simon et al., 1983) |

**Supplementary File 4B:** Plasmids used in this study.

| **Plasmid trivial name** | **Plasmid**  **stock name** | **Comment** | **Origin, marker** | **Reference** |
| --- | --- | --- | --- | --- |
| pEVS143 | pEVS143 | Constitutive over-expression plasmid | p15A, KanR | (Dunn et al.,  2006) |
| pKAS32 | pKAS32 | Suicide plasmid for allelic exchange | R6K, AmpR | (Skorupski and  Taylor, 1996) |
| pXG10-SF | pXG10-SF | Control plasmid | pSC101*, CmR | (Corcoran et al.,  2012) |
| pCMW-1 | pCMW-1 | Control plasmid | p15A, KanR | (Waters and  Bassler, 2006) |
| pCMW-2 | pLH001 | Control plasmid | p15A, KanR | This study |
| pBAD1K-ctrl | pMD004 | Control plasmid | p15A, KanR | This study |
| pBAD1K-*oppZ* | pMD089 | *oppZ* expression plasmid | p15A, KanR | This study |
| pBAD1K-*oppB*::3xFlag | pMD373 | *oppB* expression plasmid | p15A, KanR | This study |
| pEVS143-*oppZ* | pMD090 | *oppZ* expression plasmid | p15A, KanR | This study |
| pEVS143-*oppZ M1* | pMD118 | *oppZ M1* expression plasmid | p15A, KanR | This study |
| pEVS143-*oppZ mutated*  *loop* | pMD194 | *oppZ mutated loop* expression plasmid | p15A, KanR | This study |
| pEVS143-*oppZ M1*  *mutated loop* | pMD195 | *oppZ M1 mutated loop* expression  plasmid | p15A, KanR | This study |
| pEVS102-ctrl | pMD397 | Control plasmid (BCM-compatible) | pSC101 *repA*  E93K, KanR | This study |
| pEVS102-*oppZ mutated*  *loop* | pMD398 | *oppZ mutated loop* expression plasmid  (BCM-compatible) | pSC101 *repA*  E93K, KanR | This study |
| pCMW-pBR | pMD173 | Control plasmid | pBR322, KanR | This study |
| pEVS143-pBR-*oppZ* | pMD174 | *oppZ* expression plasmid | pBR322, KanR | This study |
| pEVS143-pBR-*oppZ*  *precursor* | pMD197 | *oppZ precursor* expression plasmid | pBR322, KanR | This study |
| pEVS143-*carZ* | pNP015 | *carZ* expression plasmid | p15A, KanR | This study |
| pEVS143-*carZ M1* | pMH013 | *carZ M1* expression plasmid | p15A, KanR | This study |
| pEVS143-*carZ mutated*  *loop* | pMD361 | *carZ mutated loop* expression plasmid | p15A, KanR | This study |
| pEVS143-*carZ mutated*  *loop M1* | pMD362 | *carZ M1 mutated loop* expression  plasmid | p15A, KanR | This study |
| pXG10-*oppB::gfp* | pMD093 | Translational reporter for *oppB* | pSC101*, CmR | This study |
| pXG10-*oppB M1::gfp* | pMD125 | Translational reporter for *oppB M1* | pSC101*, CmR | This study |
| pXG10-*carA* | pMH010 | Translational reporter for *carA* | pSC101*, CmR | This study |
| pXG10-*carA M1* | pMH012 | Translational reporter for *carA M1* | pSC101*, CmR | This study |
| pXG10-*carAB* | pMD374 | Translational reporter for *carAB* | pSC101*, CmR | This study |
| pXG10-*carAB M1* | pMD375 | Translational reporter for *carAB M1* | pSC101*, CmR | This study |
| pXG10-  *mKate::oppB::gfp* | pMD120 | Discoordinate translational reporter for  *oppB* | pSC101*, CmR | This study |
| pXG10-*mKate::oppB*  *M1::gfp* | pMD129 | Discoordinate translational reporter for  *oppB M1* | pSC101*, CmR | This study |
| pXG10-  *mKate::oppC::gfp* | pMD352 | Discoordinate translational reporter for  *oppBC* | pSC101*, CmR | This study |

| pXG10-  *mKate::oppD::gfp* | pMD353 | Discoordinate translational reporter for  *oppBCD* | pSC101*, CmR | This study |
| --- | --- | --- | --- | --- |
| pXG10-  *mKate::oppF::gfp* | pMD354 | Discoordinate translational reporter for  *oppBCDF* | pSC101*, CmR | This study |
| pXG10-*mKate::oppF*  *M1::gfp* | pMD355 | Discoordinate translational reporter for  *oppBCDF M1* | pSC101*, CmR | This study |
| pXG10-*oppZ* | pMD091 | Processed *oppZ* expression | pSC101*, CmR | This study |
| pXG10-*oppZ M2* | pMD124 | Processed *oppZ* expression | pSC101*, CmR | This study |
| pXG10-*oppB::gfp-oppZ* | pMD112 | Translational reporter for *oppB* with  *oppZ* at 3’end | pSC101*, CmR | This study |
| pXG10-*oppB::gfp-oppZ*  *M1* | pMD117 | Translational reporter for *oppB* with  *oppZ M1* at 3’end | pSC101*, CmR | This study |
| pXG10-*oppB::gfp-oppZ*  *M2* | pMD126 | Translational reporter for *oppB* with  *oppZ M2* at 3’end | pSC101*, CmR | This study |
| pXG10-*oppB M1::gfp-*  *oppZ* | pMD127 | Translational reporter for *oppB M1* with  *oppZ* at 3’end | pSC101*, CmR | This study |
| pXG10-*oppB M1::gfp-*  *oppZ M1* | pMD128 | Translational reporter for *oppB M1* with  *oppZ M1* at 3’end | pSC101*, CmR | This study |
| pXG10-*carA::gfp-carZ* | pMD294 | Translational reporter for *carA* with *carZ*  at 3’end | pSC101*, CmR | This study |
| pXG10-*carA::gfp-carZ*  *M1* | pMD295 | Translational reporter for *carA* with *carZ*  *M1* at 3’end | pSC101*, CmR | This study |
| pXG10-*carA M1::gfp-*  *carZ* | pMD297 | Translational reporter for *carA M1* with  *carZ* at 3’end | pSC101*, CmR | This study |
| pXG10-*carA M1::gfp-*  *carZ M1* | pMD298 | Translational reporter for *carA M1* with  *carZ M1* at 3’end | pSC101*, CmR | This study |
| pKAS32-rne3071 | pMD003 | Suicide plasmid for temperature-  sensitive *rne* allele | R6K, AmpR | This study |
| pKAS32-∆*oppZ* | pMD160 | Suicide plasmid for *oppZ* knockout | R6K, AmpR | This study |
| pKAS32-*oppB*::ATC | pMD349 | *oppB* start codon mutation by allelic  replacement | R6K, AmpR | This study |
| pKAS32-*oppB*::2nd stop | pMD357 | *oppB* 2nd codon mutated to stop codon  by allelic replacement | R6K, AmpR | This study |
| pKAS32-*oppB*::15th stop | pMD358 | *oppB* 15th codon mutated to stop codon  by allelic replacement | R6K, AmpR | This study |
| pKAS32-*oppB*::65th stop | pMD370 | *oppB* 65th codon mutated to stop codon  by allelic replacement | R6K, AmpR | This study |
| pKAS32-*oppB*::115th  stop | pMD371 | *oppB* 115th codon mutated to stop  codon by allelic replacement | R6K, AmpR | This study |
| pKAS32-*oppB*::215th  stop | pMD372 | *oppB* 215th codon mutated to stop  codon by allelic replacement | R6K, AmpR | This study |
| pKAS32-*oppC*::ATC | pMD356 | *oppC* start codon mutation by allelic  replacement | R6K, AmpR | This study |
| pKAS32-*oppD*::ATC | pMD367 | *oppD* start codon mutation by allelic  replacement | R6K, AmpR | This study |
| pKAS32-*oppF*::CTG | pMD369 | *oppF* start codon mutation by allelic  replacement | R6K, AmpR | This study |
| pKAS32-*oppA*::3xFlag | pMD199 | *oppA*::3xFlag allelic replacement | R6K, AmpR | This study |
| pKAS32-*oppB*::3xFlag | pMD200 | *oppB*::3xFlag allelic replacement | R6K, AmpR | This study |
| pKAS32-*oppF*::3xFlag | pMD269 | *oppF*::3xFlag allelic replacement | R6K, AmpR | This study |
| pKAS32-∆*carZ* | pMD350 | Suicide plasmid for *carZ* knockout | R6K, AmpR | This study |

| pKAS32-*carA*::3xFlag | pMD346 | *carA*::3xFlag allelic replacement | R6K, AmpR | This study |
| --- | --- | --- | --- | --- |
| pKAS32-*carB*::3xFlag | pMD347 | *carB*::3xFlag allelic replacement | R6K, AmpR | This study |
| pKAS32-  P*oppABCDF*::pBAD | pMD280 | P*oppABCDF*::pBAD allelic replacement | R6K, AmpR | This study |
| pKAS32-P*carAB*::pBAD | pMD351 | P*carAB*::pBAD allelic replacement | R6K, AmpR | This study |
| pKAS32-*carA*::2nd stop | pMD385 | *carA* 2nd codon mutated to stop codon  by allelic replacement | R6K, AmpR | This study |
| pKAS32-*carB*::2nd stop | pMD386 | *carB* 2nd codon mutated to stop codon  by allelic replacement | R6K, AmpR | This study |

**Supplementary File 4C:** DNA oligonucleotides used in this study.

| **ID** | **Sequence (5’→3’)** | **Description** |
| --- | --- | --- |
| KPO-1484 | GCTCAATCAATCACCGGATCAAGGCCCAGTCTTTCGAC | pMD004 |
| KPO-1485 | CGCAACTCTCTACTGTTTCTCCTTTTTTCTAGATTAAATCAGAACGCAG | pMD004 |
| KPO-0196 | GGAGAAACAGTAGAGAGTTGCG | pMD004, pMD089, pMD280, pMD351 |
| KPO-1397 | GATCCGGTGATTGATTGAGC | pMD004, pMD089, pMD090 |
| KPO-5878 | GCTAACAGGAGGAATTAACCATGCTTAAATTCATAGCAAAAAGG | pMD373 |
| KPO-5879 | CTACTGCCGCCAGGCATTAtttatcgtcatctttgtagtc | pMD373 |
| KPO-2789 | TGCCTGGCGGCAGTAGC | pMD373 |
| pBAD-  ATGrev | GGTTAATTCCTCCTGTTAGC | pMD373 |
| KPO-2757 | TGAGGATCCGGTGATTGATTGAGCA | pLH001 |
| KPO-5421 | TGCTCAATCAATCACCGGATCCTCAGCATGCaAAAAGACCCTTC | pLH001 |
| KPO-2552 | CGCAACTCTCTACTGTTTCTCCATTCAGCCTGTGACGGG | pMD089 |
| KPO-2553 | GCTCAATCAATCACCGGATCAGGCGATTGGTCGTGTTG | pMD089, pMD090, pMD197 |
| KPO-2568 | TCGGCTCGTATAATGTGTGGATTCAGCCTGTGACGGG | pMD090 |
| KPO-0092 | CCACACATTATACGAGCCGA | pMD090, pNP015 |
| KPO-2619 | GTATAATGTGTGGATTCAGCCTGTcACGGGCCAAGCGCGATTC | pMD118 |
| KPO-2620 | GAATCGCGCTTGGCCCGTgACAGGCTGAATCCACACATTATAC | pMD118 |
| KPO-1013 | TATTACAACAAGAGAGGCTCAA | pNP015 |
| KPO-1014 | gtttttTCTAGACAGACGCTACATCAAACTGAA | pNP015 |
| KPO-1023 | gtttttTCTAGAGGATCCGGTGATTGATTGAG | pNP015 |
| KPO-1782 | GCGGGCAAAGAGGTTcTCAGAGAGGCCTTGAGC | pMH013, pMD296, pMD298 |
| KPO-1783 | GCTCAAGGCCTCTCTGAgAACCTCTTTGCCCGC | pMH013, pMD296, pMD298 |
| KPO-3190 | CGATTgCagcaagGcATCGCGCTTTTTTTGTATTCG | pMD194, pMD195 |
| KPO-3191 | GCGATgCcttgctGcAATCGCGCTTGGCCCG | pMD194, pMD195 |
| KPO-6490 | AGATGCCAGGAAGATACTTAAC GACAGTAAGACGGGTAAGCC | pMD397, pMD398 |
| KPO-6491 | GATCTTATAAAAAAGGCGCATTG ATGATTTTTTCCCCACGGGAG | pMD397, pMD398 |
| KPO-6492 | ACCCA cAaCTCaaa GGAAAAGGACTAGTAATTATCATTG | pMD397, pMD398 |
| KPO-6493 | CTTTTCC tttGAGtTg TGGGTATCTGTAAATTCTGCTAG | pMD397, pMD398 |
| KPO-6494 | GTTAAAGGCTTTa AGATTTTCCAGTGGACAAACTATG | pMD397, pMD398 |
| KPO-6495 | GGAAAATCTt AAAGCCTTTAACCAAAGGATTCC | pMD397, pMD398 |
| KPO-2041 | CAATGCGCCTTTTTTATAAGATC | pMD173, pMD174, pMD397, pMD398 |
| KPO-2042 | TGCCAGGAAGATACTTAACCAGACCCCGTAGAAAAGATCAAAG | pMD173, pMD174 |
| KPO-2043 | TCTTATAAAAAAGGCGCATTGGAACATGTGAGCAAAAGGCCAG | pMD173, pMD174 |
| KPO-2049 | GTTAAGTATCTTCCTGGCATCT | pMD173, pMD174, pMD397, pMD398 |
| KPO-3197 | TCGGCTCGTATAATGTGTGGTTGCCCGCAAGCCACG | pMD197 |
| KPO-5686 | GAGAccgagttccggTCTCTGACAACCTCTTTGCC | pMD361, pMD362 |
| KPO-5687 | GAccggaactcggTCTCTTGTTGTAATACCACAC | pMD361, pMD362 |
| KPO-1702 | ATGCATGTGCTCAGTATCTCTATC | pXG10 derivates |

| KPO-1703 | GCTAGCGGATCCGCTGG | pXG10 derivates |
| --- | --- | --- |
| KPO-2580 | GAGATACTGAGCACATGCATTAGAGCCATATTCCTAACGAG | pMD093, pMD112 |
| KPO-2583 | GAGCCAGCGGATCCGCTAGCTATGAATTTAAGCATAAGCTCACTC | pMD093, pMD112, pMD120 |
| KPO-2615 | CAGGGTCTTTGCATTTCCTTGTgACAGATTGAAAGAGTGAGCTTATG | pMD125, pMD127,  pMD129, pMD355 |
| KPO-2616 | CATAAGCTCACTCTTTCAATCTGTcACAAGGAAATGCAAAGACCCTG | pMD125, pMD127, pMD129, pMD355 |
| KPO-1674 | TGATAGAGATACTGAGCACATGCATAATTTGTCTATAGCATCCTAATG | pMH010, pMD374 |
| KPO-1675 | CCAGCGGATCCGCTAGCGATGGACACTCCGCGG | pMH010 |
| KPO-5874 | CCAGCGGATCCGCTAGCGATAACAATTGGACCCGCAC | pMD374 |
| KPO-1778 | GCATTTTTATTCTGGAGGTTcTCTTGAGTAAATCAGCAC | pMH012, pMD375 |
| KPO-1779 | GTGCTGATTTACTCAAGAgAACCTCCAGAATAAAAATGC | pMH012, pMD375 |
| KPO-2577 | gtttttGCTAGCAATCTCAAGGTTTTGTGAGAAG | pMD352 |
| KPO-2578 | gtttttGCTAGCAATACCGTCTTGGGTAGAG | pMD353 |
| KPO-2579 | gtttttGCTAGCAAGATCTTTAACGTCCAGTAATA | pMD354 |
| KPO-2511 | AGGCCTAGTTGCGTACAGG | pMD120, pMD352- 354 |
| KPO-2625 | TTATCTGTGCCCCAGTTTGC | pMD120, pMD352- 354 |
| KPO-2621 | CCTGTACGCAACTAGGCCTATGCATGTGCTCAGTATCTCTATC | pMD120, pMD352- 354 |
| KPO-2622 | GCAAACTGGGGCACAGATAATAGAGCCATATTCCTAACGAG | pMD120, pMD352-  354 |
| KPO-2585 | GCATGGATGAGCTCTACAAATAAATTCAGCCTGTGACGGG | pMD091, pMD112 |
| KPO-2586 | TCGTTTTATTTGATGCCTCTAGAAGGCGATTGGTCGTGTTG | pMD091, pMD112 |
| KPO-2584 | TTTGTAGAGCTCATCCATGC | pMD091, pMD112, pMD294, pMD297 |
| KPO-2508 | TCTAGAGGCATCAAATAAAACGAAAG | pMD091, pMD112, pMD294, pMD297 |
| KPO-2617 | GCTCTACAAATAAATTCAGCCTGTcACGGGCCAAGCGCGATTC | pMD117, pMD128 |
| KPO-2618 | GAATCGCGCTTGGCCCGTgACAGGCTGAATTTATTTGTAGAGC | pMD117, pMD128 |
| KPO-2665 | ACAAATAAAggCAGCCTGTGACGGGCCAAG | pMD124, pMD126 |
| KPO-2666 | CACAGGCTGccTTTATTTGTAGAGCTCATCCATGC | pMD124, pMD126 |
| KPO-4815 | CATGGATGAGCTCTACAAATAATTGGTATTACAACAAGAGAGGC | pMD294, pMD297 |
| KPO-4817 | GTTTTATTTGATGCCTCTAGACAGACGCTACATCAAACTGAAC | pMD294, pMD297 |
| KPO-0267 | TAATAGGCCTAGGATGCATATG | pKAS32 derivates |
| KPO-0268 | CGTTAACAACCGGTACCTCTA | pKAS32 derivates |
| KPO-1440 | TAGAGGTACCGGTTGTTAACGCTTCGAGCTTGTATTCTGC | pMD003 |
| KPO-1441 | CATATGCATCCTAGGCCTATTACAGAGAAGGGCTCAAACG | pMD003 |
| KPO-1442 | GCTGAAAGACACGGTTTCtTCCCTCTCAAAGAAATCGC | pMD003 |
| KPO-1443 | GCGATTTCTTTGAGAGGGAaGAAACCGTGTCTTTCAGC | pMD003 |
| KPO-2753 | TAGAGGTACCGGTTGTTAACGGAAATCGGCATGGCCATGG | pMD160 |
| KPO-1199 | TTATGCCTCAGCGTATAAACAAG | pMD160 |
| KPO-1200 | GTTTATACGCTGAGGCATAAGCGCGATTCCTCGTTCGG | pMD160 |
| KPO-2754 | CATATGCATCCTAGGCCTATTACTTAAATAACCGCTCAGATGTGG | pMD160 |
| KPO-1429 | TAGAGGTACCGGTTGTTAACGCTGAAGACCGTGAACGTTTCC | pMD350 |
| KPO-1289 | GCTGACGAGCGGGCAAAGCAATTAAGCTTGGTTCGCTTTTAC | pMD350 |
| KPO-1290 | CTTTGCCCGCTCGTCAGC | pMD350 |

| KPO-1430 | CATATGCATCCTAGGCCTATTACAATGTGACAATATAAGACCAGC | pMD350 |
| --- | --- | --- |
| KPO-5243 | TAGAGGTACCGGTTGTTAACGGATGAACCGTTTCCTATCGG | pMD349, pMD357, pMD358 |
| KPO-5244 | GAATTTAAGgATAAGCTCACTCTTTCAATCTGTG | pMD349 |
| KPO-5245 | GTGAGCTTATcCTTAAATTCATAGCAAAAAGGATATTTG | pMD349 |
| KPO-5246 | CATATGCATCCTAGGCCTATTATGCGACCTTGAATCTCAAGG | pMD349, pMD357, pMD358 |
| KPO-3183 | TAGAGGTACCGGTTGTTAACGCTATAAATAACGTAGACGCTGC | pMD356 |
| KPO-5670 | TTTTTCGTCAAgATTATCTAAGTCCTATTAtttatcgtc | pMD356 |
| KPO-5671 | GGACTTAGATAatcTTGACGAAAAAAGAAAATTTAGCGG | pMD356 |
| KPO-3186 | CATATGCATCCTAGGCCTATTACAATACCGTCTTGGGTAGAG | pMD356 |
| KPO-5672 | GCTATGAATTTttaCATAAGCTCACTCTTTCAATCTG | pMD357 |
| KPO-5673 | GAGTGAGCTTATGtaaAAATTCATAGCAAAAAGGATATTTG | pMD357 |
| KPO-5674 | CACCAACATttaTGGAATTGCTTCAAATATCCTTTTTG | pMD358 |
| KPO-5675 | GCAATTCCAtaaATGTTGGTGTTGATTACTATATC | pMD358 |
| KPO-5880 | TAGAGGTACCGGTTGTTAACGCGAAGAAATTGCTGGAAGAAG | pMD370, pMD371 |
| KPO-5881 | CATATGCATCCTAGGCCTATTAGAGGTCGCGACCAAGAG | pMD370, pMD371 |
| KPO-5882 | TAGAGGTACCGGTTGTTAACGGTACCACAGTGCTGAATACG | pMD372 |
| KPO-5883 | CATATGCATCCTAGGCCTATTATTGCTACGTAGGCTCAAGG | pMD372 |
| KPO-5884 | GTTGGTCAAATAttaTAAATACTGTTCAGAAACGGGTT | pMD370 |
| KPO-5885 | GAACAGTATTTAtaaTATTTGACCAACATTGTTCAGG | pMD370 |
| KPO-5886 | CAATGGTTCCttaCGTTACCCCCATGATCACAG | pMD371 |
| KPO-5887 | GGGTAACGtaaGGAACCATTGCCGCCCTG | pMD371 |
| KPO-5890 | ATAGCCATAttaCAAACCTTTCGCACGTGCG | pMD372 |
| KPO-5891 | CGAAAGGTTTGtaaTATGGCTATATCGTGGTCAAGC | pMD372 |
| KPO-4395 | TAGAGGTACCGGTTGTTAACGGTTTAACGCGATTGTCGATATTC | pMD367 |
| KPO-5824 | AATAAACTCATgATTGCTTCCTTTTTATTCTTCTTGG | pMD367 |
| KPO-5823 | AAGGAAGCAATcATGAGTTTATTAGATGTCAAAGATC | pMD367 |
| KPO-4400 | CATATGCATCCTAGGCCTATTAAAGATCTTTAACGTCCAGTAATAATTT | pMD367 |
| KPO-4379 | TAGAGGTACCGGTTGTTAACGTAAACCAAGAAGAATAAAAAGGAAGC | pMD369 |
| KPO-5828 | CAAGCCTCCCAgTCAGAAAAACACGCACGTTGAC | pMD369 |
| KPO-5827 | GTGTTTTTCTGAcTGGGAGGCTTGGACAAAATG | pMD369 |
| KPO-4384 | CATATGCATCCTAGGCCTATTAGAATTTATGCCTCAGCGTATAAAC | pMD369 |
| KPO-3179 | TAGAGGTACCGGTTGTTAACGAAAACTTTGTCGGTAACGGTG | pMD199 |
| KPO-3180 | cgtcatggtctttgtagtcTTGAGCTTTGATGTAAAGATCTTTG | pMD199 |
| KPO-3181 | gactacaaagatgacgataaaTAATAGAGCCATATTCCTAACG | pMD199 |
| KPO-3182 | CATATGCATCCTAGGCCTATTAACCGATCAGGATCGTTACC | pMD199 |
| KPO-3183 | TAGAGGTACCGGTTGTTAACGCTATAAATAACGTAGACGCTGC | pMD200 |
| KPO-3184 | cgtcatggtctttgtagtcGTAGCGAATCTTCGGATCG | pMD200 |
| KPO-3185 | gactacaaagatgacgataaaTAATAGGACTTAGATAATGTTGACG | pMD200 |
| KPO-3186 | CATATGCATCCTAGGCCTATTACAATACCGTCTTGGGTAGAG | pMD200 |
| KPO-3157 | gactacaaagaccatgacggtgattataaagatcatgatatcgactacaaagatgacgataaa | pMD199, pMD200 |

| KPO-3158 | tttatcgtcatctttgtagtcgatatcatgatctttataatcaccgtcatggtctttgtagtc | pMD199, pMD200 |
| --- | --- | --- |
| KPO-4385 | TAGAGGTACCGGTTGTTAACGTGAGCGTAGAGAAAAAATTATTACTG | pMD269 |
| KPO-4386 | TGCCTCAGCGTATAAACAAGAG | pMD269 |
| KPO-4387 | CTCTTGTTTATACGCTGAGGCAgactacaaagaccatgacgg | pMD269 |
| KPO-4388 | GCCCGTCACAGGCTGAATTTAtttatcgtcatctttgtagtcgat | pMD269 |
| KPO-4389 | TAAATTCAGCCTGTGACGGG | pMD269 |
| KPO-4390 | CATATGCATCCTAGGCCTATTAATCGACCGTCTCTTGGATGC | pMD269 |
| KPO-5223 | TAGAGGTACCGGTTGTTAACGCCTTCTTATTCCCAGCAAATC | pMD346, pMD386 |
| KPO-5224 | AGCGCGGAATTGTTTAATCAG | pMD346 |
| KPO-5225 | TAATTTGGAGTAGTAGATAATGCC | pMD346 |
| KPO-5226 | CATATGCATCCTAGGCCTATTAGAAACCCACTGCCAGTTTG | pMD346, pMD386 |
| KPO-5231 | CTGATTAAACAATTCCGCGCTgactacaaagaccatgacgg | pMD346 |
| KPO-5232 | GCATTATCTACTACTCCAAATTAtttatcgtcatctttgtagtcgat | pMD346 |
| KPO-5227 | TAGAGGTACCGGTTGTTAACGGTCGAATGAATCACCAGTTCTG | pMD347 |
| KPO-5228 | AGCTTGGTTCGCTTTTACACG | pMD347 |
| KPO-5229 | TAATTGGTATTACAACAAGAGAGG | pMD347 |
| KPO-5230 | CATATGCATCCTAGGCCTATTACAACGGCAACTTTGATCTTAAG | pMD347 |
| KPO-5233 | CGTGTAAAAGCGAACCAAGCTgactacaaagaccatgacgg | pMD347 |
| KPO-5234 | CTCTCTTGTTGTAATACCAATTAtttatcgtcatctttgtagtcgat | pMD347 |
| KPO-4529 | TATAAGATCATAAAAGACCCTTCATTTATG | pMD280, pMD351 |
| KPO-4527 | TAGAGGTACCGGTTGTTAACGGGACACCTGATCTGAGTGC | pMD280 |
| KPO-4528 | GAAGGGTCTTTTATGATCTTATACAATGTGCCAGATTATCTAGTG | pMD280 |
| KPO-4530 | CGCAACTCTCTACTGTTTCTCCAATCCCTGTCAGGTGTAAGTTG | pMD280 |
| KPO-4531 | CATATGCATCCTAGGCCTATTACTTGCACTTGAACAGATTCTGG | pMD280 |
| KPO-5235 | TAGAGGTACCGGTTGTTAACGGCAATCTGGTAAAAGCAACAC | pMD351, pMD385 |
| KPO-5236 | GAAGGGTCTTTTATGATCTTATAGAAAGAACATTTTAAAAATGGAAATAG | pMD351 |
| KPO-5237 | CGCAACTCTCTACTGTTTCTCCAATTTGTCTATAGCATCCTAATGC | pMD351 |
| KPO-5238 | CATATGCATCCTAGGCCTATTATCCAAATCTTTCACTGGATGG | pMD351, pMD385 |
| KPO-6029 | GTGCTGATTTttaCAAGACAACCTCCAGAATAAAAATG | pMD385 |
| KPO-6030 | GTTGTCTTGtaaAAATCAGCACTGTTAGTCCTAG | pMD385 |
| KPO-6031 | TCAGTACGTTTttaCATTATCTACTACTCCAAATTAtttatc | pMD386 |
| KPO-6032 | GTAGATAATGtaaAAACGTACTGACATTCAAAGCATC | pMD386 |
| KPO-0243 | TTCGTTTCACTTCTGAGTTCGG | 5S rRNA oligoprobe |
| KPO-0145 | GGTTACAAAAACTGTTAGTCGTG | MicX oligoprobe |
| KPO-2025 | ATTAGGCGATGAGATAATACG | Vcr041 oligoprobe |
| KPO-0842 | GTAAAGCAATTAACTTACGCCAATTG | Vcr043 oligoprobe |
| KPO-2026 | GTGATTTTCTTGTTGGTTAGC | Vcr044 oligoprobe |
| KPO-0845 | TTGGCCCGTCACAGGCTGAA | OppZ oligoprobe |
| KPO-0852 | TTGAATCTGCAGGTTGGCTATAG | Vcr053 oligoprobe |
| KPO-0860 | GGAACGAAATAGGAAAGGAACGA | Vcr064 oligoprobe |

| KPO-0861 | CAAAGAGCCTGCGCTAATGGA | Vcr065 oligoprobe |
| --- | --- | --- |
| KPO-0873 | CTCTCCATGGGACAGAGTCT | FarS oligoprobe |
| KPO-0875 | AGGGTTACAACGCACCATTTCA | Vcr079 oligoprobe |
| KPO-2040 | GTGAATCATATCGACCAAATTTG | Vcr082 oligoprobe |
| KPO-0821 | AGGTTGTCAGAGAGGCCTTGA | CarZ oligoprobe |
| KPO-3990 | TTCCCTGTAGGTTATCCCCTTA | Vcr016 riboprobe |
| KPO-3991 | gtttttttttTAATACGACTCACTATAGGGAGGAAAGAAAGCGCCCTAGATTGG | Vcr016 riboprobe |
| KPO-2687 | GAATCGCGCTTGGCCCG | OppZ oligoprobe |
| KPO-2688 | GATCCGAACGAGGAATCG | OppZ native loop oligoprobe |
| KPO-3192 | GATgCcttgctGcAATC | OppZ mutated loop  oligoprobe |
| KPO-2482 | AAAAAAGCCCGCTGACGAG | CarZ oligoprobe |
| KPO-5822 | AccggaactcggTCTCTTGTTGTA | CarZ mutated loop oligoprobe |
| KPO-3201 | CAACAATCAGCCGTTCTGAATAC | *oppA* qRT-PCR |
| KPO-3202 | AAAGAGGTGAATGACGTGGTAG | *oppA* qRT-PCR |
| KPO-3203 | GTGCAGGGTCTTTGCATTTC | *oppB* qRT-PCR |
| KPO-3204 | TCGTTGGAATTGCTTCAAATATCC | *oppB* qRT-PCR |
| KPO-3205 | CGCGCGTATTCGCTTTATG | *oppC* qRT-PCR |
| KPO-3206 | GGCATACCAGTCCGTATCTTC | *oppC* qRT-PCR |
| KPO-3207 | TTAGGTATTGTGGGCGAATCC | *oppD* qRT-PCR |
| KPO-3208 | CTGGCAAGTTGAGGATTTCTTTAC | *oppD* qRT-PCR |
| KPO-3209 | TAACGTGCGTCTGTATGAAGG | *oppF* qRT-PCR |
| KPO-3210 | CTGACCTAACCACACCACTTC | *oppF* qRT-PCR |
| KPO-5870 | TGGTATTTCCGTCGGTGAAG | *carA* qRT-PCR |
| KPO-5871 | GAGTAACGATTTGCTGGGAATAAG | *carA* qRT-PCR |
| KPO-5872 | GAAGAGGGTTACCGCGTTATT | *carB* qRT-PCR |
| KPO-5873 | CAGTGGATAGGCTCGATGTAAG | *carB* qRT-PCR |
| KPO-2378 | GGTAACCCAGAAACTACCACTG | *recA* qRT-PCR |
| KPO-2379 | CACCACTTCTTCGCCTTCTT | *recA* qRT-PCR |
| KPO-3751 | ACCTGATTCCATCCCGAA | 5S rRNA qRT-PCR |
| KPO-3752 | TGGCGATGTTCTACTCTCA | 5S rRNA qRT-PCR |
